# Periductal Fibrosis and Cholangiocarcinoma-Related Outcomes in Liver Fluke-Endemic Regions: A Systematic Review and Meta-Analysis

**DOI:** 10.3390/medsci14030380

**Published:** 2026-07-09

**Authors:** Wanlaya Naowaratwattana, Hasaya Dokduang, Wipavadee Daiponmak, Darunee Puangpronpitag, Araya Supawat, Issarapong Phosuk, Jurairat Jongthawin

**Affiliations:** 1 Faculty of Medicine, Mahasarakham University, Maha Sarakham 44000, Thailand; wanlaya.n@msu.ac.th (W.N.); hasaya.d@msu.ac.th (H.D.); wipavadee.d@msu.ac.th (W.D.); darunee.p@msu.ac.th (D.P.); araya.su@msu.ac.th (A.S.); 2 Biomedical Science Research Unit, Mahasarakham University, Maha Sarakham 44000, Thailand; 3 International and National Collaborative Network and Innovation for Community Health Development Research Unit, Mahasarakham University, Maha Sarakham 44000, Thailand; 4 Department of Public Health, Amnatcharoen Campus, Mahidol University, Amnat Charoen 37000, Thailand; issarapong.pho@mahidol.ac.th

**Keywords:** periductal fibrosis, cholangiocarcinoma, *Opisthorchis viverrini*, ultrasonography, meta-analysis, endemic regions, cancer

## Abstract

**Background/Objectives:** Periductal fibrosis (PDF) is a common hepatobiliary abnormality associated with chronic *Opisthorchis viverrini* infection and may represent an ultrasonographic marker of chronic biliary injury in liver fluke-endemic regions. This systematic review and meta-analysis evaluated the association between ultrasound-defined PDF and cholangiocarcinoma (CCA)-related outcomes and summarized the prevalence of overall PDF and advanced periductal fibrosis (APF) in endemic populations. **Methods:** PubMed, Embase, and Scopus were searched from database inception to April 2026, supplemented by manual screening of reference lists. Observational studies reporting PDF prevalence and/or the association between PDF and CCA-related outcomes were included. Pooled odds ratios (ORs) and prevalence estimates were calculated using random-effects models. Five studies met the inclusion criteria. **Results**: Three studies involving 758,686 participants were included in the pooled association analysis. Ultrasound-defined PDF was associated with higher odds of CCA-related outcomes, including confirmed CCA, incident CCA, and clinically suspected CCA, with a pooled OR of 2.77 (95% CI: 2.24–3.44; I^2^ = 0%). Because outcome definitions, effect measures, adjustment status, and PDF exposure categories varied across studies, this estimate should be interpreted as a summary association rather than as a precise estimate of confirmed CCA risk. Four studies involving 759,117 participants contributed to the overall PDF prevalence analysis, whereas three studies reported APF prevalence data. Prevalence estimates varied substantially across endemic settings, with extreme between-study heterogeneity. **Conclusions:** Overall, ultrasound-defined PDF may help identify individuals who warrant closer surveillance or further evaluation; however, it should not be interpreted as a definitive histopathological diagnosis or a proven causal precursor of CCA. Further prospective studies using standardized fibrosis definitions, blinded ultrasonographic assessment, confirmed or incident CCA outcomes, and adjustment for *O. viverrini* infection-related factors and other key confounders are needed to validate the role of PDF in CCA risk stratification.

## 1. Introduction

Cholangiocarcinoma (CCA) is a highly aggressive malignancy arising from the biliary epithelium and remains a major hepatobiliary cancer burden in Southeast Asia, particularly in regions endemic for *Opisthorchis viverrini* infection [1,2]. Despite advances in diagnostic imaging and clinical management, CCA is frequently diagnosed at advanced stages and is associated with poor prognosis, limited therapeutic options, and high mortality rates [1]. The persistently high incidence of CCA in endemic areas highlights the need for practical markers that may help identify individuals who warrant closer surveillance in high-risk populations.

Chronic *O. viverrini* infection induces persistent biliary inflammation, oxidative stress, epithelial injury, and progressive fibrotic remodeling of the bile ducts [2]. Among the hepatobiliary abnormalities associated with chronic liver fluke infection, periductal fibrosis (PDF) is an important ultrasonographic finding [3,4]. Ultrasound-defined PDF may reflect chronic periductal or periportal fibro-inflammatory change resulting from repeated cycles of inflammation and tissue repair, although it should not be interpreted as direct histopathological confirmation of periductal fibrosis. Large population-based screening initiatives, including the Cholangiocarcinoma Screening and Care Program (CASCAP), have demonstrated the feasibility of ultrasonographic assessment for detecting hepatobiliary abnormalities in high-risk populations [5,6,7,8,9]. Consequently, ultrasound-defined PDF has been considered a marker of chronic biliary injury and a potential imaging-based indicator associated with CCA-related outcomes in endemic settings [9,10,11].

Several epidemiological studies have reported associations between ultrasound-defined PDF and CCA-related outcomes; however, the magnitude and consistency of this relationship remain incompletely understood [9,10,11]. Considerable heterogeneity exists across studies regarding study design, population characteristics, outcome definitions, fibrosis classification systems, and diagnostic criteria [3,4,9,10,11]. Some studies classify PDF using binary criteria [10,11], whereas others use graded ultrasonographic severity systems based on anatomical bile duct involvement, fibrosis extent, or echogenic patterns [3,4,9]. These differences limit direct comparability across the literature and create uncertainty regarding whether study-specific effect estimates reflect any PDF, advanced periductal fibrosis (APF), or the most severe fibrosis category available within individual studies. In addition, one study reported stronger associations in more advanced fibrosis categories, but evidence remains limited and has not been systematically synthesized [9].

In addition to evaluating the association between PDF and CCA-related outcomes, understanding the distribution of PDF across endemic settings may help contextualize the burden of chronic hepatobiliary abnormalities in populations affected by liver fluke infection. Population-based screening studies have reported varying prevalence estimates of overall PDF and APF according to geographic setting, infection intensity, study population characteristics, ultrasound protocols, and ultrasonographic classification systems [3,4,9,10,11]. Because prevalence estimates differ substantially across studies, synthesis of available evidence may help describe the extent of epidemiological and methodological variability across endemic populations.

Therefore, this systematic review and meta-analysis primarily aimed to evaluate the association between ultrasound-defined PDF and CCA-related outcomes in liver fluke-endemic populations and secondarily to summarize the prevalence of overall PDF and APF across endemic settings. In addition, this study sought to describe study-specific fibrosis classification systems and harmonized analytical categories to clarify how PDF and APF were defined across the available evidence.

## 2. Materials and Methods

### 2.1. Protocol and Registration

The protocol for this systematic review and meta-analysis was prospectively registered in the International Prospective Register of Systematic Reviews (PROSPERO; registration number CRD420261373426). This review was conducted and reported in accordance with the Preferred Reporting Items for Systematic Reviews and Meta-Analyses (PRISMA) guidelines [12].

### 2.2. Search Strategy

A comprehensive literature search was conducted across three electronic databases: PubMed, Scopus, and Embase, from database inception to April 2026. The search strategy combined controlled vocabulary terms, including Medical Subject Headings (MeSH) and Embase Subject Headings (Emtree), with free-text terms related to periductal fibrosis, cholangiocarcinoma, and *Opisthorchis viverrini* infection using Boolean operators. The core search terms included (“periductal fibrosis” OR “PDF” OR “periportal fibrosis”) AND (“cholangiocarcinoma” OR “CCA” OR “bile duct cancer”) AND (“*Opisthorchis viverrini*” OR “liver fluke infection”). Additional manual screening of reference lists from relevant studies and review articles was performed to identify potentially eligible studies not captured through the electronic database search. No restrictions were applied regarding publication year or language in order to maximize retrieval sensitivity. The full search strategy for each database is provided in Table S1.

### 2.3. Eligibility Criteria

Study selection was performed according to predefined inclusion and exclusion criteria based on the PECO framework [13]. The population (P) comprised human participants residing in cholangiocarcinoma-endemic regions, particularly areas with a high prevalence of liver fluke infection in Southeast Asia. The exposure (E) was ultrasound-defined periductal fibrosis (PDF). The comparator (C), where applicable, included individuals without PDF. The outcomes (O) included: (i) the association between PDF and CCA-related outcomes, reported as effect estimates such as odds ratios (ORs) or relative risks (RRs), and/or (ii) the prevalence of overall PDF or advanced periductal fibrosis (APF). Studies were eligible if they: (i) were observational studies conducted in endemic populations; (ii) assessed PDF using ultrasonography; (iii) reported effect estimates related to CCA-related outcomes and/or PDF prevalence data; and (iv) provided sufficient data for quantitative synthesis. Reviews, editorials, conference abstracts, case reports, animal studies, and studies lacking extractable outcome data were excluded. When overlapping study populations were identified, only the most comprehensive or most relevant dataset for the planned analysis was included.

### 2.4. Exposure Definition

Periductal fibrosis (PDF) was assessed using abdominal ultrasonography in all included studies. The original PDF definitions and grading systems varied across studies, including binary PDF classification, anatomical bile duct-based grading, extent-based grading, and echogenic pattern-based grading. Therefore, PDF categories were harmonized separately according to the planned analyses. For the prevalence synthesis, overall PDF was defined as any ultrasonography-defined PDF-positive category where available. Advanced periductal fibrosis (APF) was defined according to the advanced fibrosis threshold reported in each original study. For the CCA-related association analysis, the exposure category followed the effect estimate reported or extractable from each original study, rather than a single standardized PDF threshold. Study-specific PDF definitions, extracted categories, harmonized classifications, and analytical roles were recorded for each included study. 

### 2.5. Study Selection and Data Extraction

All retrieved records were imported into EndNote version 21.0 (Clarivate Analytics, Philadelphia, PA, USA) for duplicate removal. Titles and abstracts were independently screened by two reviewers, followed by full-text assessment against the predefined eligibility criteria. Reasons for exclusion were recorded for all reports excluded during full-text assessment. Relevant data were extracted into Microsoft Excel 2021 (Microsoft Corporation, Redmond, WA, USA) using a standardized, pilot-tested data extraction form. Extracted variables included first author, publication year, country, study design, participant characteristics, PDF assessment method, fibrosis classification system, prevalence estimates, outcome definitions, and effect measures for CCA-related outcomes. Any disagreements were resolved through discussion and consensus, or by consultation with a third reviewer when required. Potentially overlapping datasets were evaluated according to study period, geographic region, screening program, study objectives, outcome definitions, and available extractable data. When multiple reports appeared to use overlapping or related screening populations, the dataset with the largest sample size, most complete outcome data, and greatest relevance to the specific analysis was retained. Moungthard et al. (2023) [9] was retained because it represented the largest and most recent CASCAP-based dataset from northeastern Thailand, with the most complete extractable data for both the CCA-related association and prevalence analyses. Mairiang et al. (2012) [3] was included only in the APF prevalence analysis, as it represented an earlier independent community-based survey conducted before the CASCAP program and did not provide extractable CCA association data. Thanakijsombat et al. (2024) [10] was retained because it was conducted in Nan Province, a geographically distinct northern Thai population, and was considered non-overlapping with the northeastern CASCAP cohort. The Lao PDR studies were also considered non-overlapping because they differed in study period, geographic setting, and available outcome data.

### 2.6. Risk of Bias Assessment

Methodological quality assessment was performed independently by two reviewers according to study design. Studies evaluating the association between PDF and CCA-related outcomes were assessed using the Newcastle–Ottawa Scale (NOS) for observational studies. The NOS evaluates methodological quality across three domains: selection of study groups, comparability of groups, and ascertainment of exposure or outcome. Studies scoring 7–9 points were considered low risk of bias, scores of 4–6 indicated moderate risk, and scores of 0–3 indicated high risk of bias [14]. For prevalence studies, methodological quality was assessed using the Joanna Briggs Institute (JBI) critical appraisal checklist for prevalence studies [15]. This tool evaluates methodological domains including sampling methodology, adequacy of sample size, reliability of outcome measurement, and appropriateness of statistical analyses. Studies with >70% “Yes” responses were considered low risk of bias, 50–69% were considered moderate risk, and <50% were considered high risk.

### 2.7. Statistical Analysis

Pooled effect sizes evaluating the association between PDF and CCA-related outcomes were calculated using random-effects models based on the DerSimonian–Laird method to account for anticipated between-study heterogeneity [16]. The included studies differed in outcome definitions, effect-measure types, adjustment status, and PDF exposure categories. Therefore, the association analysis was summarized across CCA-related outcomes, including confirmed CCA, incident CCA, and clinically suspected CCA. One included cohort study reported a relative risk (RR), whereas the other studies reported or allowed calculation of odds ratios (ORs). The RR was treated as an approximation of the OR under the rare disease assumption because CCA is a relatively uncommon outcome in population-based screening settings and because the number of eligible studies was limited. For the CCA-related association analysis, the exposure category followed the effect estimate reported or extractable from each original study. In Moungthard et al. (2023) [9], adjusted ORs were reported separately for PDF1, PDF2, and PDF3. The PDF3 versus no-PDF estimate was used because it represented the highest fibrosis category available from the original adjusted model, whereas comparable grade-specific estimates were not available across the other included studies. When effect estimates were not directly reported, crude ORs were calculated using standard 2 × 2 contingency tables reconstructed from the available raw data.

For prevalence meta-analyses, pooled prevalence estimates were calculated using generalized linear mixed models (GLMMs) with a logit link to account for the binomial distribution of prevalence data [17]. Random-effects models were applied to account for anticipated between-study heterogeneity, and pooled estimates were back-transformed and presented as proportions with 95% confidence intervals. Statistical heterogeneity was assessed using Cochran’s Q test and quantified using the I^2^ statistic and tau^2^ (between-study variance) [18]. I^2^ values of 25%, 50%, and 75% were interpreted as low, moderate, and high heterogeneity, respectively. A *p*-value < 0.10 for Cochran’s Q test was considered indicative of statistically significant heterogeneity.

Subgroup analyses were stratified according to predefined study characteristics, including geographic region and fibrosis classification systems, when sufficient data were available. Sensitivity analyses were performed using a leave-one-out approach to evaluate the stability of the pooled estimates. For the association analysis, additional sensitivity analyses were conducted to examine the influence of outcome definition and effect-measure type. Specifically, we performed a sensitivity analysis excluding the study that used clinically suspected CCA as the outcome and another sensitivity analysis excluding the study that reported an RR. Assessment of publication bias using funnel plots or Egger’s regression test was not performed because statistical methods for publication bias assessment are considered unreliable when fewer than 10 studies are available for pooled analyses. All statistical analyses were conducted using RStudio (Version 2024.04.2+764) [19].

## 3. Results

### 3.1. Search Results

**Figure 1:**
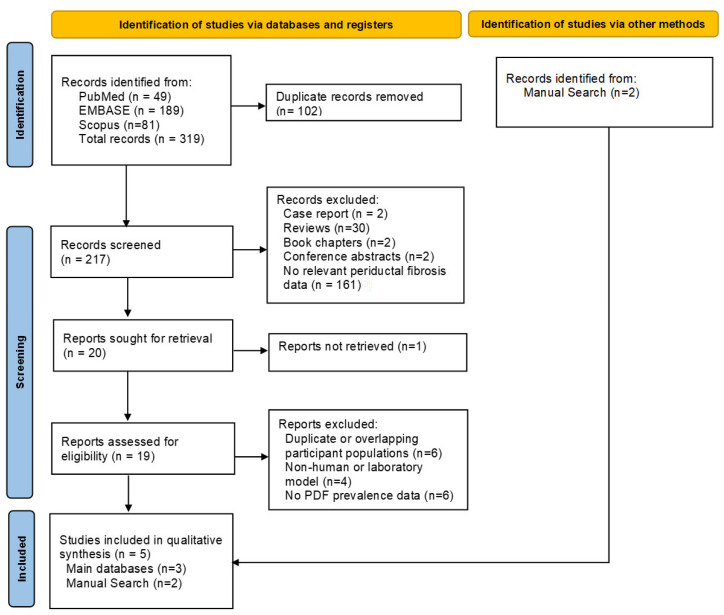
The PRISMA 2020 flow diagram outlines the process of study selection for the review.

A total of 319 records were identified from PubMed, Embase, and Scopus. After removing 102 duplicates, 217 records were screened, and 20 full-text reports were sought for retrieval. One report could not be retrieved, leaving 19 full-text reports assessed for eligibility. Of these, 16 reports were excluded for duplicate or overlapping populations, non-human or laboratory-based designs, or insufficient PDF prevalence data. Three studies were eligible from database searches, and two additional studies were identified through manual searching. Ultimately, five studies were included in the qualitative synthesis and meta-analysis (Figure 1).

### 3.2. Characteristics of Included Studies

Five studies from liver fluke-endemic regions of Southeast Asia were included, comprising four cross-sectional studies and one cohort study conducted in Thailand and Lao PDR. All studies assessed PDF using ultrasonography, although PDF classification differed across studies, including binary PDF classification and graded ultrasonographic severity classifications. Three studies evaluated the association between PDF and CCA-related outcomes, whereas the remaining studies contributed data for overall PDF and/or APF prevalence. The main study characteristics, outcome definitions, and effect estimates, are presented in Table 1. Study-specific PDF definitions, extracted fibrosis categories, harmonized prevalence categories, exposure categories used in the CCA-related association analysis, and analytical roles in the synthesis are presented in Table 2.

### 3.3. Quality of Included Studies

The methodological quality of the included studies is summarized in Table S2. All three studies included in the pooled analysis evaluating the association between PDF and CCA-related outcomes were classified as high quality, with NOS scores ranging from 7 to 9 stars. Similarly, all five prevalence studies were considered high quality according to the JBI critical appraisal checklist. No study was judged to be at high risk of bias, and all studies were included in the quantitative synthesis.

### 3.4. Association Between Periductal Fibrosis and Cholangiocarcinoma-Related Outcomes

**Figure 2:**
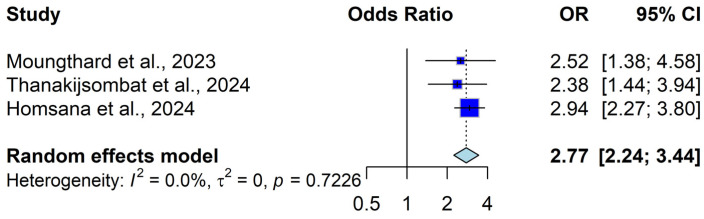
Forest plot showing the association between ultrasound-defined PDF and CCA-related outcomes using a random-effects model. The squares represent the study-specific odds ratios, with the size of each square proportional to the study weight. The vertical solid line represents the line of no effect (OR = 1), while the vertical dashed line indicates the pooled effect estimate. The diamond represents the pooled odds ratio with its 95% confidence interval [9,10,11].

Three studies involving 758,686 participants were included in the meta-analysis evaluating the association between ultrasound-defined PDF and CCA-related outcomes. The included studies reported heterogeneous outcome definitions, including confirmed CCA, incident CCA, and clinically suspected CCA, as summarized in Table 1. The pooled analysis showed that PDF was associated with higher odds of CCA-related outcomes, with a pooled OR of 2.77 (95% CI: 2.24–3.44; Figure 2). No significant statistical heterogeneity was observed among the included studies (I^2^ = 0.0%, *p* = 0.722). The study-specific PDF exposure categories used in the association analysis are summarized in Table 2.

### 3.5. Prevalence of Overall Periductal Fibrosis

**Figure 3:**
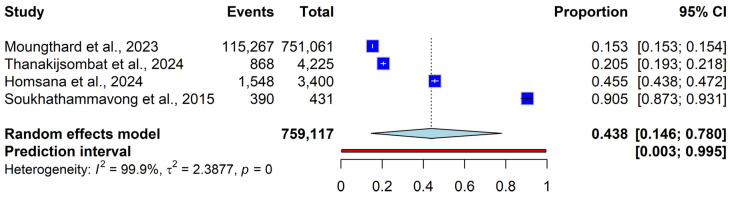
Forest plot showing the prevalence of overall PDF across included studies using a random-effects model. The squares represent study-specific prevalence estimates and are displayed with equal size for visual clarity. The diamond represents the pooled prevalence estimate with its 95% confidence interval. The vertical dashed line indicates the pooled prevalence estimate, and the red horizontal line represents the 95% prediction interval [4,9,10,11].

Four studies involving 759,117 participants were included in the meta-analysis of overall PDF prevalence. One additional study (Mairiang et al., 2012) [3] reported only APF prevalence data and was therefore not included in this analysis. Reported prevalence estimates varied widely across studies, ranging from 15.3% to 90.5% (Figure 3). Under a random-effects model, the pooled prevalence of overall PDF was 43.8% (95% CI: 14.6–78.0%). Extreme between-study heterogeneity was observed (I^2^ = 99.9%, τ^2^ = 2.3877, *p* < 0.0001). The 95% prediction interval was very wide (0.0–99.0%), indicating that overall PDF prevalence in future endemic populations may vary substantially. Therefore, the pooled prevalence estimate should be interpreted as a descriptive summary rather than a precise population-level estimate.

**Figure 4:**
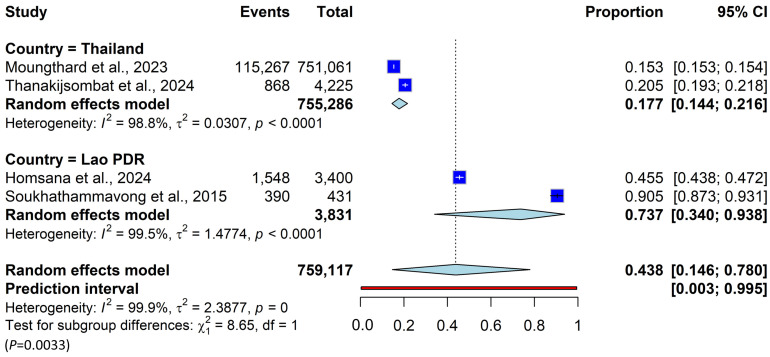
Forest plot showing the country-stratified prevalence of overall PDF across included studies using a random-effects model. The squares represent study-specific prevalence estimates and are displayed with equal size for visual clarity. The diamonds represent the subgroup-specific and overall pooled prevalence estimates with their 95% confidence intervals. The vertical dashed line indicates the overall pooled prevalence estimate, and the red horizontal line represents the 95% prediction interval [4,9,10,11].

In the country-stratified analysis, the pooled prevalence of overall PDF was 17.7% (95% CI: 14.4–21.6%) in Thailand and 73.7% (95% CI: 34.0–93.8%) in Lao PDR (Figure 4). Substantial heterogeneity was present within both country subgroups. These subgroup estimates were retained as descriptive summaries because each subgroup included only a small number of studies.

### 3.6. Prevalence of Advanced Periductal Fibrosis (APF)

**Figure 5:**
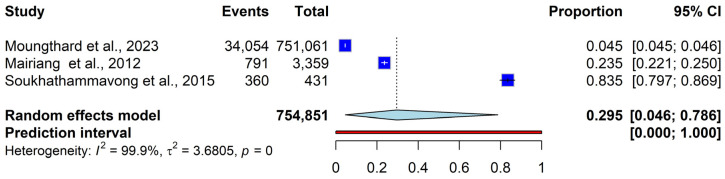
Forest plot showing the prevalence of APF across included studies using a random-effects model. The squares represent study-specific prevalence estimates and are displayed with equal size for visual clarity. The diamond represents the pooled prevalence estimate with its 95% confidence interval. The vertical dashed line indicates the pooled prevalence estimate, and the red horizontal line represents the 95% prediction interval [3,4,9].

Three studies involving 754,851 participants were included in the meta-analysis of APF prevalence. Reported APF prevalence estimates varied widely across studies, ranging from 4.5% to 83.5% (Figure 5). Under a random-effects model, the pooled prevalence of APF was 29.5% (95% CI: 4.6–78.6%). Extreme between-study heterogeneity was observed (I^2^ = 99.9%, τ^2^ = 3.6805, *p* < 0.001). The 95% prediction interval was extremely wide (0.0–100.0%), indicating that APF prevalence in future endemic populations may vary substantially. Therefore, the pooled APF prevalence estimate should be interpreted as a descriptive summary rather than a precise population-level estimate.

**Figure 6:**
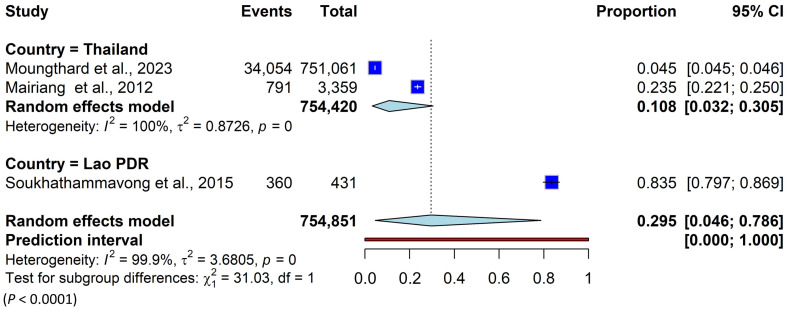
Forest plot showing the country-stratified prevalence of ADF across included studies using a random-effects model. The squares represent study-specific prevalence estimates and are displayed with equal size for visual clarity. The diamonds represent the subgroup-specific and overall pooled prevalence estimates with their 95% confidence intervals. The vertical dashed line indicates the overall pooled prevalence estimate, and the red horizontal line represents the 95% prediction interval [3,4,9].

In the country-stratified analysis, the pooled APF prevalence was 10.8% (95% CI: 3.2–30.5%) in Thailand, based on two studies. The Lao PDR estimate was derived from a single study reporting an APF prevalence of 83.5% (95% CI: 79.7–86.9%) (Figure 6). These subgroup findings were retained as descriptive summaries because of the small number of studies and the single-study estimate for Lao PDR.

### 3.7. Sensitivity Analysis

Leave-one-out sensitivity analysis for the association between PDF and CCA-related outcomes showed that the direction of association remained consistent across iterations, with pooled ORs remaining above 1.0 and statistical heterogeneity remaining low (I^2^ = 0.0%; Supplementary Figure S1). In addition to the leave-one-out analysis, further sensitivity analyses were conducted to evaluate the influence of outcome definition and effect-measure type. When Homsana et al. (2024) [11], the study using clinically suspected CCA as the outcome, was excluded, the pooled estimate among studies reporting confirmed or incident CCA remained positive (OR = 2.44, 95% CI: 1.66–3.58) (Supplementary Figure S2). When Thanakijsombat et al. (2024) [10], the study reporting an RR, was excluded, the OR-based pooled estimate also remained positive (OR = 2.87, 95% CI: 2.27–3.64) (Supplementary Figure S3). These sensitivity analyses should be interpreted cautiously because each analysis included only two studies and should therefore be considered supportive rather than definitive.

In contrast, leave-one-out sensitivity analyses for overall PDF prevalence and APF prevalence showed substantial variability in pooled estimates, while extreme heterogeneity persisted across iterations (I^2^ > 99%). Exclusion of Soukhathammavong et al. (2015) [4] markedly reduced the pooled prevalence estimates for both overall PDF and APF (Supplementary Figures S4 and S5), indicating that prevalence estimates were strongly influenced by individual studies. Therefore, pooled prevalence estimates, particularly for APF, should be interpreted as descriptive summaries rather than precise population-level estimates.

## 4. Discussion

This systematic review and meta-analysis found that ultrasound-defined PDF was associated with higher odds of CCA-related outcomes in liver fluke-endemic populations. However, the pooled estimate should be interpreted with caution because the included studies differed in outcome definitions, effect-measure types, adjustment status, and PDF exposure categories. The association analysis combined confirmed CCA, incident CCA, and clinically suspected CCA, and included adjusted ORs, one crude OR reconstructed from available data, and one RR treated as an approximation of an OR. Therefore, the pooled result should be viewed as a summary association across heterogeneously defined CCA-related outcomes rather than as a precise quantitative estimate of confirmed CCA risk.

The observed association is biologically plausible and is consistent with the established role of chronic *O. viverrini* infection in biliary inflammation and hepatobiliary morbidity. Chronic liver fluke infection can induce persistent biliary inflammation, oxidative and nitrative stress, epithelial injury, and fibrotic remodeling of the bile ducts [2]. These processes may contribute to a biliary microenvironment associated with epithelial injury and carcinogenesis [20]. In this context, ultrasound-defined PDF may serve as a marker of cumulative biliary injury or chronic hepatobiliary exposure. However, PDF should not be interpreted as a direct causal precursor, a proven premalignant lesion, or evidence of a biologically advanced carcinogenic stage based on the current evidence alone. Although one included study used a cohort design, most available studies were cross-sectional, and the overall evidence remains insufficient to firmly establish temporal or causal relationships between PDF and CCA-related outcomes. PDF may also represent a correlate of coexisting hepatobiliary abnormalities or a finding detected more frequently within intensive ultrasonographic surveillance settings. Therefore, the present findings should be interpreted as evidence of association rather than causation.

The interpretation of ultrasound-defined PDF also requires caution because it may not always correspond to histologically confirmed periductal fibrosis. Although all included studies assessed PDF using ultrasonography, direct histopathological validation of ultrasonographic PDF in liver fluke-endemic screening populations remains limited. Histopathological confirmation would require invasive tissue sampling, such as biopsy or surgical specimens, which is not routinely feasible or ethically justified in asymptomatic individuals undergoing community-based screening. Moreover, histopathological data, when available, are generally derived from clinically indicated biopsy or resection specimens and may not be representative of the broader screened population. Therefore, ultrasound-defined PDF should be regarded as a non-invasive screening or epidemiological marker of chronic periductal or periportal fibro-inflammatory change rather than direct pathological confirmation of periductal fibrosis.

The exposure definition also requires cautious interpretation. Although all included studies assessed PDF using ultrasonography, the original classification systems were not identical. Some studies used binary PDF definitions [10,11], whereas others applied anatomical bile duct-based grading, extent-based grading, or echogenic pattern-based grading [3,4,9]. For the CCA-related association analysis, the exposure category followed the effect estimate reported or extractable from each original study. In Moungthard et al. (2023) [9], adjusted ORs were reported separately for PDF1, PDF2, and PDF3; the PDF3 versus no-PDF estimate was used because it represented the highest fibrosis category available from the original adjusted model, whereas comparable grade-specific estimates were not available across the other included studies. Therefore, the pooled association should not be interpreted as a fully standardized estimate for overall PDF or APF. Rather, it summarizes the available evidence across study-specific ultrasound-defined PDF categories.

Limited evidence from studies using graded ultrasonographic classifications suggests that more advanced fibrosis categories may be associated with higher CCA-related outcomes. In Moungthard et al. (2023) [9], PDF2 and PDF3, but not PDF1, were associated with CCA, with the strongest association observed for PDF3. Similarly, studies reporting APF suggest that advanced fibrosis may represent more extensive hepatobiliary abnormality in endemic populations. Nevertheless, the present meta-analysis was not designed to formally evaluate a dose–response relationship by fibrosis severity because comparable grade-specific estimates were not available across studies. Future longitudinal studies using standardized fibrosis grading systems are needed to clarify whether increasing PDF severity independently predicts subsequent CCA development.

Prevalence analyses were included as a secondary descriptive component of this review. Reported prevalence estimates of overall PDF and APF varied substantially across endemic settings, and extreme between-study heterogeneity was observed. This variability likely reflects differences in geographic setting, transmission intensity, study population, participant selection, ultrasound protocols, diagnostic criteria, and fibrosis classification systems. Therefore, pooled prevalence estimates should not be interpreted as precise summary estimates for any specific endemic population. Instead, they should be considered descriptive indicators of broad epidemiological and methodological variability across the available studies. Country-stratified subgroup findings should also be interpreted cautiously because each subgroup included only a small number of studies, and the Lao PDR APF estimate was derived from a single high-prevalence study.

Despite these limitations, the findings may still have practical relevance for endemic settings. Ultrasonography is widely used in liver fluke-endemic areas because it is non-invasive, accessible, relatively low-cost, and feasible for large-scale population-based screening. In Thailand, regional screening initiatives such as the Cholangiocarcinoma Screening and Care Program (CASCAP) illustrate how ultrasonography can be incorporated into public health surveillance frameworks for high-risk populations [5,6,7,8,9]. Within such settings, ultrasound-defined PDF may help identify individuals with chronic hepatobiliary abnormalities who may warrant closer clinical follow-up or additional evaluation. However, the current evidence is not sufficient to establish PDF as a definitive independent risk-stratification tool for CCA. Its clinical utility should be validated in prospective studies using standardized ultrasound protocols, blinded imaging assessment, confirmed or incident CCA outcomes, appropriate adjustment for key confounders, and, where feasible, integration with molecular biomarkers. Inflammatory, tumor-associated, and fibrosis-related biomarkers, such as IL-6, CA19-9, and microRNAs, may reflect biological changes associated with liver fluke–related hepatobiliary injury [21,22,23,24,25] and could potentially complement imaging-based fibrosis assessment. Nevertheless, biomarker-integrated surveillance strategies require prospective validation before they can be recommended for routine clinical or public health implementation.

Several limitations should be acknowledged. First, only a small number of eligible studies were available, and most were observational or cross-sectional, limiting causal inference. Second, residual confounding cannot be excluded. Active *O. viverrini* infection status, infection intensity based on egg counts, cumulative exposure, reinfection, and previous praziquantel or other anthelmintic treatment may influence both PDF development and CCA-related outcomes. However, adjustment for these variables was inconsistent across included studies. Moungthard et al. (2023) [9] reported adjusted ORs after controlling for gender, age at enrollment, education level, history of *O. viverrini* infection, smoking history, and alcohol consumption history. Nevertheless, current infection status, infection intensity, reinfection, previous anthelmintic treatment, dietary exposures, and coexisting hepatobiliary comorbidities were not consistently adjusted across the available studies. The PDF-related estimate from Homsana et al. (2024) [11] was reconstructed as a crude OR despite the availability of *O. viverrini* infection and intensity data, and Thanakijsombat et al. (2024) [10] did not report adjustment for *O. viverrini*–related variables for the PDF estimate used in the synthesis. Therefore, residual confounding remains possible and supports cautious interpretation of the pooled association.

Third, the pooled association combined heterogeneous outcome definitions, including clinically suspected CCA, which remains distinct from histologically confirmed or incident CCA and may introduce outcome misclassification [11]. Fourth, the pooled association combined adjusted, crude, and RR-based estimates. Although sensitivity analyses excluding the suspected CCA study and excluding the RR study showed directionally consistent positive associations, these restricted analyses included only two studies and should be considered supportive rather than definitive. Fifth, substantial heterogeneity existed in PDF and APF definitions, including binary, anatomical bile duct-based, extent-based, and echogenic pattern-based ultrasonographic classifications, which limited direct comparability across studies [3,4,9,10,11].

Sixth, all included studies assessed PDF using ultrasonography. Ultrasonographic assessment may be influenced by operator expertise, image quality, equipment characteristics, probe selection, and the severity of fibrotic or inflammatory changes. Across the included studies, published data on the sensitivity, specificity, and inter-observer reproducibility of ultrasonographic PDF assessment were limited, partly because histopathological confirmation is rarely feasible in asymptomatic community-based screening populations [3,4,9,10,11]. Ultrasonographic features used to define PDF, including periductal thickening and increased periportal echogenicity, are not entirely specific to liver fluke–associated fibrosis and may overlap with cholangiocarcinoma, primary sclerosing cholangitis, recurrent pyogenic cholangitis, or other chronic inflammatory biliary disorders [26,27]. Thus, ultrasound-defined PDF should be considered a screening or epidemiological marker rather than a definitive disease-specific or histopathological diagnosis. In established screening settings, individuals with advanced, atypical, or progressive periductal changes may require further evaluation using higher-resolution imaging modalities such as CT, MRI, or MRCP where clinically indicated [28]. Finally, reverse causation remains possible, particularly in cross-sectional studies, and publication bias could not be formally assessed because fewer than 10 studies were available for pooled analyses.

Despite these limitations, this review provides a structured synthesis of the available evidence on ultrasound-defined PDF, APF prevalence, and their association with CCA-related outcomes in liver fluke-endemic populations. The findings suggest that PDF may be a marker of chronic biliary injury associated with CCA-related outcomes, but the strength and precision of this association remain limited by heterogeneous definitions, outcome misclassification, residual confounding, and the small number of studies. Future prospective studies using standardized PDF and APF definitions, blinded ultrasonographic assessment, formal inter-observer reproducibility testing, validation with advanced imaging modalities where feasible, and confirmed or incident CCA outcomes are needed. Such studies should also adjust for current *O. viverrini* infection status, infection intensity, reinfection, previous praziquantel or other anthelmintic treatment, age, sex, dietary exposures, hepatobiliary comorbidities, and other established CCA risk factors to clarify the role of PDF in CCA risk stratification and surveillance.

## 5. Conclusions

Ultrasound-defined PDF was associated with higher odds of CCA-related outcomes in liver fluke-endemic populations. However, because the available evidence was limited by heterogeneous outcome definitions, effect measures, adjustment status, and PDF exposure categories, the pooled estimate should be interpreted as a summary association rather than as a precise estimate of confirmed CCA risk. Prevalence estimates of overall PDF and advanced periductal fibrosis varied substantially across endemic settings, reflecting considerable epidemiological and methodological heterogeneity. Overall, ultrasound-defined PDF may serve as a non-invasive marker of chronic biliary injury and may help identify individuals who warrant closer surveillance or further evaluation, but it should not be interpreted as a definitive histopathological diagnosis, a proven causal precursor, or evidence of a biologically advanced carcinogenic stage of CCA. Further prospective studies with standardized fibrosis assessment, confirmed or incident CCA outcomes, and appropriate adjustment for key confounders are needed to clarify the role of PDF in CCA risk stratification.

## Figures and Tables

**Table 1:** Characteristics of studies included in the systematic review and meta-analysis.

Study	Country	Study Design	Sample Size	Mean Age, Years	Male, %	PDF Assessment	Outcome	Effect Estimates
Moungthard et al., 2023 [9]	Thailand	Cross-sectional	751,061	54.9	36.8	US; PDF grading	Confirmed CCA	Adjusted OR for PDF3 vs. no-PDF = 2.52 (95% CI: 1.38–4.58)
Thanakijsombat et al., 2024 [10]	Thailand	Cohort	4225	45.5	45.4	US; binary PDF	Incident CCA	RR 2.38 (95% CI: 1.44–3.94)
Homsana et al., 2024 [11]	Lao PDR	Cross-sectional	3400	50.0	40.2	US; binary PDF	Suspected CCA *	Crude OR 2.94 (95% CI: 2.27–3.80) **
Mairiang et al., 2012 [3]	Thailand	Cross-sectional	3359	46.1	50.1	US; PDF grading	APF prevalence	Not reported
Soukhathammavong et al., 2015 [4]	Lao PDR	Cross-sectional	431	43.0	42.2	US; PDF grading	PDF/APF prevalence	Not reported

Abbreviations: APF, advanced periductal fibrosis; CCA, cholangiocarcinoma; CI, confidence interval; OR, odds ratio; PDF, periductal fibrosis; RR, relative risk; US, ultrasonography. Footnote: * Suspected CCA in Homsana et al. (2024) [9] was defined by prespecified ultrasonographic findings, including liver mass, intrahepatic/extrahepatic bile duct dilatation, or shotgun sign, while excluding typical benign lesions and stone-related bile duct dilatation. ** The crude OR for Homsana et al. (2024) [9]. was calculated from available raw data using a 2 × 2 contingency table.

**Table 2:** Study-specific definitions and harmonized analytical classification of periductal fibrosis.

Study	Original US Classification	Extracted Data	Harmonized Prevalence Category	Exposure for Association	Role in Synthesis
Moungthard et al., 2023 [9]	Anatomical bile duct-based grading: PDF1, peripheral small bile ducts; PDF2, segmental bile ducts; PDF3, main bile duct involvement.	PDF1: 81,213 PDF2: 30,048 PDF3: 4006 Overall PDF: 115,267 APF: 34,054	Overall PDF = PDF1–PDF3 APF = PDF2–PDF3	PDF3 vs. no-PDF	Included in CCA- related association analysis, overall PDF prevalence analysis, and APF prevalence analysis
Thanakijsombat et al., 2024 [10]	Binary PDF classification based on increased periportal echogenicity caused by bile duct wall thickening parallel to the portal vein.	PDF positive: 868	Overall PDF = PDF positive	PDF positive vs. PDF negative	Included in CCA- related association analysis and overall PDF prevalence analysis
Homsana et al., 2024 [11]	Binary/IPE-based PDF definition based on increased periportal echo reflecting bile duct wall thickening along the portal vein distribution.	PDF positive: 1548	Overall PDF = PDF positive	PDF positive vs. PDF negative	Included in CCA- related association analysis and overall PDF prevalence analysis
Mairiang et al., 2012 [3]	Extent-based grading according to the number of liver segments with periportal echoes: grade 0, normal; grade I, <2 segments; grade II, 2–3 segments; grade III, >3 segments; APF = grade ≥ II.	Grade II: 742 Grade III: 49 APF: 791	APF = grade ≥ II	Not applicable	Included only in APF prevalence analysis
Soukhathammavong et al., 2015 [4]	Echogenic pattern-based grading: grade 0, normal/no echoes; grade 1+, starry-sky; grade 2+, rings-and-pipe-stems; grade 3+, highly echogenic peripheral patches; APF = grade ≥ 2+.	Grade 1+: 30 Grade 2+: 287 Grade 3+: 73 Overall PDF: 390 APF: 360	Overall PDF = grade 1+–3+ APF = grade ≥ 2+	Not applicable	Included in overall PDF prevalence analysis and APF prevalence analysis

Abbreviations: APF, advanced periductal fibrosis; CCA, cholangiocarcinoma; IPE, increased periportal echo; PDF, periductal fibrosis. Footnote: Sample sizes for each study are presented in Table 1. The extracted data shown in this table represent the number of participants in each PDF or APF category used for harmonized prevalence synthesis. Overall PDF was harmonized as any ultrasonography-defined PDF-positive category where available, whereas APF was harmonized according to the advanced fibrosis threshold reported in each original study. Because grading systems were not identical across studies, harmonized categories were used for analytical transparency and should not be considered fully equivalent. For the CCA-related association analysis, the exposure category followed the effect estimate reported or extractable from each original study.

## Data Availability

The original contributions presented in this study are included in the article/Supplementary Material. Further inquiries can be directed to the corresponding author.

## Supplementary Materials

The following supporting information can be downloaded at: https://www.mdpi.com/article/10.3390/medsci14030380/s1. Table S1. Search strategies used for each database. Table S2. Methodological quality assessment of included studies. Supplementary Figure S1. Leave-one-out sensitivity analysis of the pooled OR for the association between PDF and CCA-related outcomes. Supplementary Figure S2. Sensitivity analysis excluding the study using clinically suspected CCA as the outcome. Supplementary Figure S3. Sensitivity analysis excluding the study reporting RR as the effect measure. Supplementary Figure S4. Leave-one-out sensitivity analysis of pooled overall PDF prevalence. Supplementary Figure S5. Leave-one-out sensitivity analysis of pooled advanced PDF prevalence. Supplementary File S1. PRISMA_2020_abstract_checklist. Supplementary File S2. PRISMA Checklist.
