# Supplementary material for: Periductal Fibrosis and Cholangiocarcinoma-Related Outcomes in Liver Fluke-Endemic Regions: A Systematic Review and Meta-Analysis
Source: Med Sci (Basel). 2026 Jul 9;14(3):380. doi: 10.3390/medsci14030380 (PMC13414246; doi:10.3390/medsci14030380)
Supplement: Supplementary file 1 [file medsci-14-00380-s001.zip › included papers/Homsana et al., 2024.pdf]

RESEARCH ARTICLE

Burden and risk factors of suspected cholangiocarcinoma in high *Opisthorchis viverrini* endemic rural communities in southern Lao PDR

Anousin Homsana[1,2,3], **Phonesavanh Southisavath**[1,2,3,4], **Kerstin Kling**[5], **Jan Hattendorf**[2,3], **Savina Vorasane**[4], **Daniel Henry Paris**[2,3], **Nicole Probst-Hensch**[2,3], **Somphou Sayasone**[1], **Peter Odermatt**[2,3]*

1 Lao Tropical and Public Health Institute, Ministry of Health, Vientiane, Lao PDR, **2** Swiss Tropical and Public Health Institute, Allschwil, Switzerland, **3** University of Basel, Basel, Switzerland, **4** Department of Radiology, Mahosot Hospital, Ministry of Health, Vientiane, Lao PDR, **5** Immunization Unit, Robert Koch Institute, Berlin, Germany

* peter.odermatt@swisstph.ch

OPEN ACCESS

Citation: Homsana A, Southisavath P, Kling K, Hattendorf J, Vorasane S, Paris DH, et al. (2024) Burden and risk factors of suspected cholangiocarcinoma in high *Opisthorchis viverrini* endemic rural communities in southern Lao PDR. PLoS Negl Trop Dis 18(11): e0012617. https://doi.org/10.1371/journal.pntd.0012617

Editor: Aysegul Taylan Ozkan, TOBB Economics and Technology University Faculty of Medicine: TOBB Ekonomi ve Teknoloji Universitesi Tip Fakultesi, TÜRKIYE

Received: February 18, 2024

Accepted: October 8, 2024

Published: November 27, 2024

Copyright: © 2024 Homsana et al. This is an open access article distributed under the terms of the Creative Commons Attribution License, which permits unrestricted use, distribution, and reproduction in any medium, provided the original author and source are credited.

Data Availability Statement: The clinical data for this study contain potentially identifying and sensitive patient information, including baseline disease characteristics, ongoing clinical follow-up and treatment, and biosamples collected from patients in specific geographical areas. These biosamples are preserved for future research, which could potentially lead to patient identification. Due to these ethical concerns, as

Abstract

Introduction

Cholangiocarcinoma (CCA) is a major contributor to hepatobiliary mortality in the Lao People's Democratic Republic (Lao PDR). Infection with the carcinogenic trematode *Opisthorchis viverrini* (OV), acquired through consumption of insufficiently-cooked river fish, is a known risk factor for the development of CCA. Together with OV, other risk factors contribute to the pathogenesis of CCA. We conducted this study to identify the burden of CCA and identify risk factors in high-risk communities in Lao PDR.

Method

A cross-sectional study was performed in Champasack and Savannakhet provinces, southern Lao PDR, where OV infection is highly endemic. We assessed hepatobiliary morbidity with abdominal ultrasound (US). In addition, multiple risk factors known or suspected to be associated with CCA were assessed such as OV infection (examined by Kato-Katz technique for stool examination), lifestyle risks (e.g. smoking and alcohol consumption by face-to-face questionnaire), co-morbidity (e.g. diabetes mellitus) and hepatitis B infection status, both serologically tested.

Results

In 3,400 participants, the overall prevalence of suspected CCA was 7.2% (95% confidence interval [95% CI] 5.4−9.6). The suspected CCA prevalence increased with age, and was higher in men at all ages. Almost all participants (88.3%) were infected with OV. In the multivariate regression analysis, suspected CCA was positively associated with OV infection (adjusted odds ratio [aOR] 3.4, 95% CI 1.7−6.5), and a history of cholecystectomy (aOR 2.7, 95% CI 1.5−4.9).

imposed by the Institutional Review Board of the National Ethics Committee for Health Research (NECHR), Ministry of Health, Lao PDR, and Lao TPHI, the data cannot be shared publicly. However, de-identified data can be made available upon reasonable request. Requests for access to the data should be directed to the Data Access Committee at NECHR, Samsenthai Road, Ban Kaognot, Sisattanak District, Vientiane Capital, Lao PDR. Tel: +856 21 250670; Fax: +856 21 214012; PO: 01000; email: nechr2021@gmail.com.

Funding: The Swiss Agency for Development and Cooperation (SDC) and the Swiss National Science Foundation (SNSF) funded our study (project #IZ07Z0-160930, R4D to PO). Die Freiwillige Akademische Gesellschaft Basel (FAG Basel, www.fag-basel.ch) provided funding for mobile ultrasound machines (KK and PO). AH and PS received scholarship allowances for PhD study supported by Amt für Ausbildungsbeiträge (AFA, www.hochschulen.bs.ch) and SNF NEWTONIAN (project #IZJFZ3_185644). The funders had no role in study design, data collection and analysis, decision to publish, or preparation of the manuscript.

Competing interests: The authors have declared that no competing interests exist.

Conclusion

Our CCA screening in high OV prevalence rural areas of Lao PDR uncovers a high public health burden, primarily driven by elevated OV infection rates. Urgent interventions are needed to curb OV infection in these communities. Age and gender disparities in suspected CCA prevalence highlight the need for targeted efforts. Beyond OV, notable factors like a history of cholecystectomy offer valuable insights for preventive strategies. This research enhances our understanding of hepatobiliary morbidity and informs public health initiatives in Lao PDR.

Author summary

Evidence has long pointed to a significant burden of liver diseases in certain regions of the Lao People's Democratic Republic (Lao PDR), primarily attributed to the *Opisthorchis viverrini* (OV) liver fluke. This parasite is transmitted to humans through the consumption of raw or undercooked fish and fish products. Chronic OV infection can lead to cholangiocarcinoma (CCA), a fatal bile duct cancer. The objective of this research was to assess the prevalence of suspected CCA in rural OV-endemic communities in the southern part of Lao PDR. We conducted a cross-sectional study in selected communities within Savannakhet and Champasack provinces.

Adults aged 35 years and older were enrolled and underwent liver disease assessment through abdominal ultrasound. OV infection was diagnosed via fecal examination, while lifestyle risk factors like smoking and alcohol consumption were evaluated through face-to-face interviews. Additionally, diabetes mellitus and hepatitis B infection status were determined serologically.

We found a high prevalence of suspected bile duct cancer (CCA) at 7.2% overall. This rate was found higher in men compared to women, and to increase with age. Most participants (88.3%) were infected with OV, posing the highest risk for suspected CCA (adjusted odds ratio [aOR] 3.4). Additionally, a history of cholecystectomy (aOR 2.7) was a significant predictor of bile duct cancer. These results provide crucial insights for controlling liver morbidity in Lao PDR, emphasizing the urgent need to address OV infection and associated risk factors for CCA in affected communities.

1. Introduction

Cholangiocarcinoma (CCA) is a primary tumor of the extra- and intrahepatic bile ducts with a high fatality rate. Its incidence rates vary around the world, with a much higher incidence in parts of the eastern world than in the west [1]. For example, the annual incidence of intrahepatic CCA (between 1990 and 2000) in the United States of America has been reported at an average of 0.85/100,000 person-years [2]; and, in most European countries, over the recent years, the mortality rate due to intrahepatic CCA has elevated to 1.1/100,000 in men and 0.75/100,000 in women [3]. However, the world's highest CCA incidence is reported in the north-eastern provinces of Thailand where the incidence among individuals 35 years and older varied from 93.8/100,000 to 317.6/100,000 person-years (data collected between 1999 and 2001) [4,5].

CCA is classified in intrahepatic CCA (iCCA), perihilar CCA (pCCA) or distal CCA (dCCA)) [6]. Typically, CCA is diagnosed only at a late stage when a fatal outcome cannot be prevented. The five-year survival rate is less than 10% [7]. Patients diagnosed with an early stage may undergo surgical treatment and have a higher rate of survival [8].

In regions endemic for OV, numerous studies have established a link between OV infection and CCA [1,2,4,5,9–16]. Furthermore, the International Agency for Research on Cancer (IARC) has classified OV as a carcinogenic agent [4,5,17]. In Southeast Asia and particularly in Northeast Thailand, OV infection is highly prevalent [9]. In Lao PDR, approximately 2.5 million people are estimated to carry the infection [17]. Despite interventions aimed at reducing OV related liver morbidity, such as yearly mass-drug administration (MDA) using Praziquantel (PZQ) in Champasack (CPS) province of Lao PDR [18], the reinfection rate remains high due to the Laotians' raw fish consumption habits.

Chronic inflammation is proposed as the key OV-induced pathway to CCA [17,19,20], which occurs from both fluke's mechanical damage (feeding and migration) and immunological reactions (parasite secretory-excretory products and mediated immune response) [21]. Such mechanisms have resulted in ulceration and remodeling of the periductal epithelium tissues leading to periductal fibrosis (PDF) and finally CCA [19].

Despite the high endemicity of OV in Lao PDR, there is limited evidence regarding the extent of CCA in the rural communities and OV contributes to the prevalence of hepatic morbidity, such as CCA, across different regions of the country. This is partly due to the absence of collective data collection systems for hepatic morbidity and a clear definitive standard for diagnosing suspected CCA using ultrasound, such as determining the degree of bile duct dilatation. There are, however, some effort revealing the burden, for instance, a retrospective study examining Lao tertiary hospital records from 2006 to 2011 found that 97.4% of 274 patients examined with US and CT scan imaging showed bile duct dilation [22].

This study addresses two evidence gaps. First, there is a lack of population-based and US-based data on the prevalence of suspected CCA in rural parts of Lao PDR with high OV infection and re-infection rates. Second, even though OV infection is a strong CCA risk factor, the likelihood of CCA development is not uniformly distributed among those infected with OV [23]. Additional risk factors may contribute to the pathogenesis of CCA [1]. Consequently, this study aims to explore the extent of suspected CCA in an OV endemic setting of Lao PDR and explore the associations to OV infection and additional risk factors with CCA.

2. Materials and methods

2.1. Ethics statement

This study was approved by the National Ethics Committee for Health Research, Ministry of Health (MoH), Vientiane, Lao PDR (Ref.no.113/2018 NECHR) and the Ethikkommission der Nordwest- und Zentralschweiz (EKNZ, Ref.no. R-2017-00869), Basel, Switzerland. The permission for the fieldwork was obtained from the MoH, the Provincial Health Offices of Champasack (CPS) and Savannakhet (SVK) provinces, the District Health Offices, and the District Office of Education and Sports of Champhone and Khong districts.

Education and Sports authorities of Champhone and Khong districts collaborated in this effort. The village authorities and residents received comprehensive explanations about the study's objectives, procedures, potential risks, and benefits. A consent form, presented in the Lao language, was carefully read with the participants, addressing any questions they had. Signed informed consent was obtained from all study participants and kept confidential. Furthermore, participants were given the choice to receive the results of their examinations. Those found infected with OV received treatment with PZQ in accordance with the Lao national

treatment guidelines [24]. Participants suspected of having CCA were referred for further radiological follow-up. In cases where CCA was confirmed, surgical operations were conducted at Mahosot Hospital in Vientiane, the capital of Lao PDR. All treatments and associated travel expenses to Vientiane were provided to the participants at no cost.

2.2. Study design and sample size

This study is a community-based, cross-sectional study in selected *O. viverrini* endemic villages in CPS and SVK provinces including adults of 35 years and older. To determine the sample size, we assumed a proportion of exposed participants of 33% and a prevalence of advanced PDF (APF+) in exposed participants of 15% [25]. We estimated that a sample size of 2,100 participants would be sufficient to detect a difference of 5 percentage points with 80% power at the 95% confidence level. Participation in the 21 villages was higher than expected, resulting in a realized sample size of 3,400.

2.3. Sampling population and study sites

The eligibility criteria encompassed (i) all residents of the selected villages and (ii) individuals aged 35 years and above. Exclusion criteria included (i) pregnancy and (ii) residents who had moved to the study villages within the last 5 years.

We utilized cluster sampling of villages based on logistical feasibility and the available data on known OV infection intensity, in collaboration with district health authorities, with the goal of including all eligible individuals in the selected villages. In total, we selected 21 villages comprising 12 from Khong district (CPS) and 9 from Champhone district (SVK). These villages were strategically situated along major natural water bodies, such as the Mekong River in CPS, and the "Sue" water reservoir, a substantial water body with aquaculture ponds in SVK (Fig 1). The field study was conducted in the villages of Khong and Champhone districts from December 2017 to February 2019.

2.4. Fieldwork procedure

The process was divided into three sequential days: (i) consenting participants were registered and provided with plastic containers labeled with participant IDs for stool collection. (ii) Participants brought their filled fecal containers to the designated study point for processing and examination, which took place on the same day. Additionally, face-to-face interviews were conducted. Participants were also given a second stool collection container along with instructions to fast in preparation for the third day's examination. (iii) Participants arrived in the morning to undergo all remaining examinations, including abdominal ultrasound, physical examination, and blood drawing. The second fecal sample was also processed on this same day. Around 25–50 participants were recruited daily until all participants in the village had been included.

A 5 ml venous blood sample was collected from each participant and stored in EDTA containers. Serum was separated from the whole blood samples through centrifugation at 1,500 rpm for 10 minutes at room temperature. These blood aliquots were promptly stored at -20˚C at the study sites for a maximum of 4 weeks before being transported to the laboratory at the Lao Tropical and Public Health Institute (Lao TPHI) in Vientiane. To ensure proper storage conditions, we maintained the cold chain using portable sample freezers designed for vehicle use. Upon arrival at Lao TPHI, both serum and ETDA blood were preserved at -80˚C until further testing.

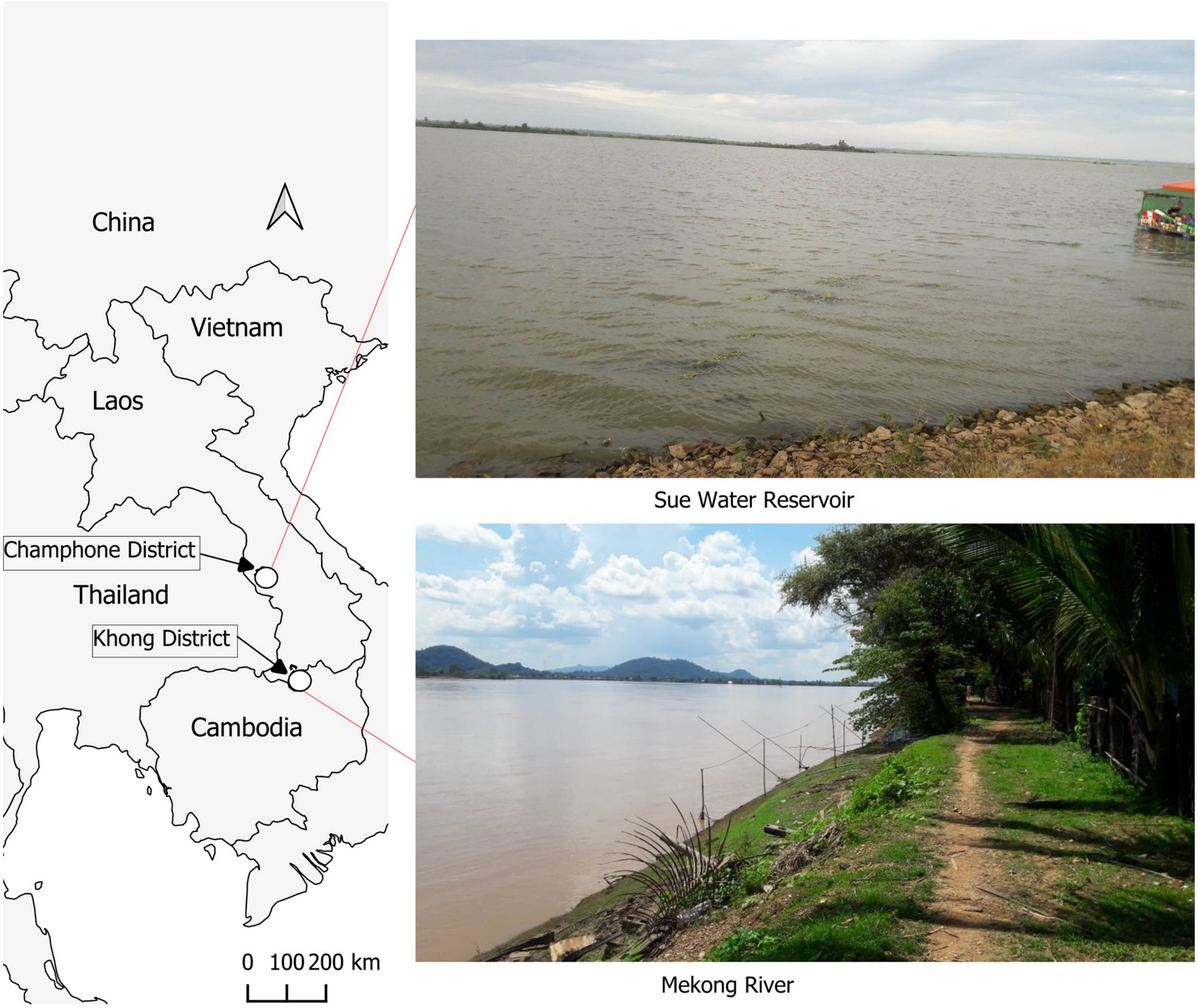

Fig 1. Map of study area. The country boundary base layer is taken from the World Bank Official Boundary (https://datacatalog.worldbank.org/search/dataset/0038272/World-Bank-Official-Boundaries) under the Creative Commons Attribution 4.0 (CC-BY 4.0) license. The photos from the field were taken by one of our research members.

https://doi.org/10.1371/journal.pntd.0012617.g001

2.5. Research tools and measurements

Abdominal ultrasound examination. Six radiologists, each with at least three years of clinical experience following residency at Lao PDR tertiary hospitals under the Lao PDR University of Health Sciences curriculum, conducted abdominal US. Additionally, an international specialist with six years of experience in US in infectious diseases also performed the US examination. To minimize errors, radiologists constantly exchanged working procedures and result assessments.

A mobile US device (Mindray Z6, Shenzhen Mindray Bio-Medical Electronics, Shenzhen, China), equipped with a convex (model 3C5P) and a linear (model 7L4P) probe, was used.

The abdominal ultrasound assessment tools used to evaluate liver pathology associated with OV infection were developed by independent research groups: Mairiang et al. [23] and

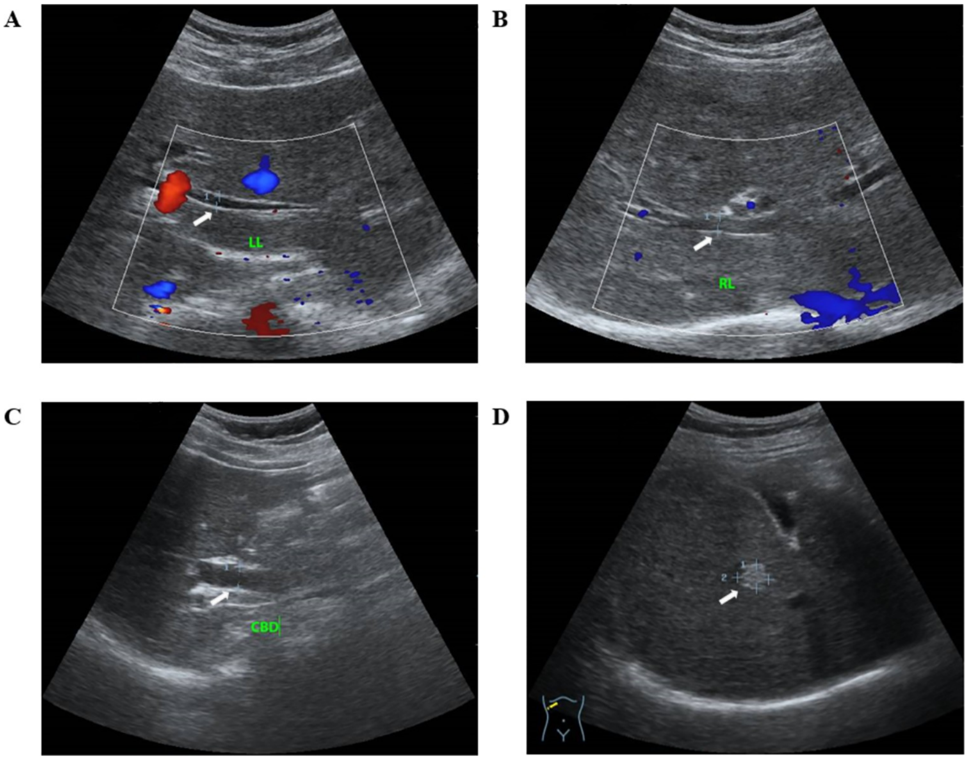

Fig 2. Ultrasound images for bile duct dilatation and liver masses. These ultrasound images, obtained from two different study participants (A-C from a 60-year-old male and D from a 63-year-old male), were classified into four categories for suspected CCA diagnosis: (A) left liver lobe (LL) dilatation (3.7 mm), (B) right liver lobe (RL) dilatation (4.8 mm), (C) common bile duct (CBD) dilatation (9.9 mm), and (D) a single hyperechoic liver mass with an ill-defined margin (9.3*11.9 mm). White arrows indicate areas of pathology, while red areas in A indicate blood flow toward the probe, and blue areas in A and B indicate blood flow away from the probe.

https://doi.org/10.1371/journal.pntd.0012617.g002

Chamadol et al. [20] both from Khon Kaen University, Thailand. Intrahepatic bile ducts diameter were measured for dilatation (at the left (LLL) and/or right liver lobes (RLL) > 2 millimeters (mm) was considered as present) [20]. Intrahepatic bile duct dilatation was also considered present when the bile duct diameter exceeds 40% of the diameter of the corresponding portal vein, also known as the "shotgun sign" [20,23]. Common bile duct (CBD) dilatation was defined as a measured diameter exceeding 6 mm (Fig 2). For participants over 60 years of age, this threshold was adjusted upward by 1 mm per decade. Additionally, in patients who had undergone cholecystectomy, only a CBD diameter of more than 10 mm was considered to indicate dilatation [26,27]. Fibrosis of the bile duct walls (PDF) was assessed using the adapted increased periportal echo protocol used in schistosomiasis proposed by Chamadol and colleagues [20].

Liver parenchyma was screened for masses [23]. The liver was examined with regard to shape, size and surface, and the liver parenchyma with regard to structure. A fatty liver was graded according to Strauss and colleagues [28], defining fatty liver into three categories: (i) a slight increase in liver echogenicity, (ii) intrahepatic vessels and diaphragm slightly impaired and increased echogenicity, and (iii) a marked increase in intra-hepatic echogenicity with poor penetration to the posterior segment of the right liver lobe, and poor or no visualization of hepatic vessels and diaphragm.

Gallbladder sludge referred to hyperechoic mobile-like images with or without posterior acoustic shadow, and gallbladder stones defined as hyperechoic structure with an acoustic

shadow [16]. Post cholecystectomy was noted when the gallbladder did not appear on the ultrasound imaging and if the patient had a history of cholecystectomy.

Questionnaires. The participants were interviewed using a standardized questionnaire with details on socio-demographic characteristics, household assets, and livestock (such as fish pond, chicken, cow, pig, water buffalo, rice cooker, radio, fan, refrigerator, television, bicycle, motorbike, farm vehicle, place to grow vegetables, cement floor, and tap water). Smoking (current smoker and ever smoker) and alcohol consumption were also recorded as consumption frequency in days per week. Additionally, food consumption behaviors such as eating raw fish and fish products (i.e., koi-pa, pickled fish) were also asked. Medical history information included self-reported diagnoses or medication intake for type 2 diabetes mellitus (T2DM), and anthelminthic treatment (e.g. PZQ). The interviews lasted between 15 and 20 minutes.

Recording the helminth infection status. The Kato-Katz technique was used for parasitological examination [29]. Participants provided two separate samples collected on different days. For each participant's sample, two glass smears were prepared according to the producer's instructions and allowed to clear for 30 to 60 minutes before examination under light microscopes by experienced microscopists from Lao TPHI. All OV and other helminth eggs were counted separately by species.

Blood examinations. All laboratory examinations were conducted at Lao TPHI. The hemoglobin A1c (HbA1c %) values were measured in EDTA blood samples, liver biochemistry parameters were measured in serum (Liver biochemical included alanine aminotransferase—ALT IU/L, aspartate aminotransferase—AST IU/L, gamma-glutamyl transferase—GGT (IU/L), alkaline phosphatase—ALP (IU/L), direct bilirubin—D-bil (mg/dL), total bilirubin—T-bil (mg/dL)). An enzymatic assay method on an automated commercially available clinical chemistry analyzer (Mindray, model: BS-240, Mindray Corporation, Shenzhen, China), following the manufacturer's instructions was used for the analysis. The testing process employed ready-to-use reagents, calibrators, and control sets for each parameter, all of which were sourced from the same manufacturer as the analyzer. Hepatitis B (HBV) infection was determined by the HBV rapid diagnostic test (Vikia HbsAg, bioMérieux, France) using serum sample.

Physical examination. The examination performed on each participant encompassed measurements of weight (rounded to the nearest 0.1 kg; Seca weighting scale, model: M 877, Hammer Steindamm 3–2522089 Hamburg, Germany) and height (rounded to the nearest 0.5 cm; Seca, model: 206, Hammer Steindamm 3–2522089 Hamburg, Germany). Furthermore, physical examinations were performed by our doctors to assess signs and symptoms associated with liver disease (S4 Appendix). This included measurement of body temperature, inspection of the skin (jaundice, spider angiomas, and abdominal collateral veins), and palpation (ascites, right upper quadrant tenderness of the abdomen, liver edge, and peripheral edema).

2.6. Data management, variables management and statistical analysis

A Commcare ODK mobile database was developed and installed on tablets utilizing Commcare server (www.commcarehq.org, version 3.4). Recoded forms were downloaded to Microsoft Excel where data cleaning and checking was performed. All data analysis was performed using STATA software, version 16.0 (StataCorp, College Stata, TX, USA).

The social economic status (SES) was calculated using assets (see section 1.5) and deriving a component scores formed by principle factor analysis. The SES score was categorized into tertile as poor, middle, and wealthy [30]. The prevalence of OV was calculated using the OV's egg seen in any microscopic slides, and the egg per 1g of stool (EPG) was calculated by multiplying the mean of OV egg counts (total OV eggs divided by 4 slides) with 24 (as single fecal sample applied in a slide is equal to 41.7mg). Four categories of OV infection intensity were calculated

(no-infection; light, EPG 1 to 999; moderate, EPG 1.000 and 9,999; and heavy, EPG $\geq$ 10,000) [31]. Body mass index (BMI) was calculated using the formula weight in kg/m^2, and classified into lean (BMI $<$ 23) and not lean (BMI $\geq$ 23) [32]. Participants were categorized as having "present" clinical symptoms for liver disease if they exhibited at least one symptom detailed in Section 1.5 (refer to S4 Appendix for a comprehensive examination).

Suspected CCA was defined based on the presence of at least one of the following US findings: (i) liver mass, (ii) dilated intrahepatic and/or extrahepatic (i.e. CBD) bile ducts, and (iii) shotgun sign [20,23]. Typical benign liver lesions, such as cysts or hemangioma, or bile duct dilation due to identified stones were not classified as suspicious for CCA (details regarding the US examination protocol described in section 2.5).

Frequencies, percentages (%), and mean (standard deviation [SD]) were used to describe categorical and numerical variables, respectively. Pearson's chi-squared (x^2) test was used to assess the independence of categorical variables. Confidence intervals of the estimated prevalence were adjusted for potential within-village correlation using random-effects models, as described below.

Logistic regression random-effects model (incorporating the variable "village" as a random effect to account for potential clustering within villages) was used to calculate crude (cOR), and adjusted odds ratio (aOR), and 95% confidence intervals (95% CI) of being suspicious for a CCA. Based on the literature on the presumed pathway to developing CCA, the primary predictors of interest (OV and HBV infection, T2DM, cholecystectomy, smoking and heavy alcohol consumption) [1,2,4,5,9–16] and a priori selected confounders (socio-economic and -demographic status) were included in the models. Models building included (i) bivariate analysis, (ii) all predictors model adjusted for age and gender, (iii) all predictors model adjusted for socio-economic and demographic confounders, (iv) all predictors model adjusted for co-morbidity (fatty liver disease, and obesity), and (v) fully adjusted model. Gallbladder co-morbidities, including gallbladder stones, gallbladder sludge, and periductal fibrosis, were excluded from the adjusted models because they are thought to be intermediate effects in the pathway between OV and suspected CCA [33,34].

Sensitivity analysis was performed to assess the robustness of the findings by excluding individuals with the following categories: (i) with T2DM (excluded due to potential ambiguity regarding its direct link to CCA pathogenesis), (ii) had high OV infection intensity (potential intrahepatic bile obstruction caused by physical blockage from adult worms), and (iii) had undergone cholecystectomy (to minimize potential bias from altered CBD physiology after surgery).

3. Results

3.1. Demographic characteristics of the participants

Out of 6,450 residents from 21 villages (aged 35 years old and older), 3,583 participants took part in the US examination, resulting in an average participation rate of 55.6% for the US examination (see S1 Appendix). In total, 3,400 study participants were included in the final analysis. 133 individuals had to be excluded because they did not provide a blood sample, and 55 individuals because they did not undergo physical examination and/or did not provide a complete set of stool samples (Fig 3).

An almost equal proportion of participants originated from CPS and SVK province (50.3% vs. 49.7%, respectively). About 60% of study participants were women. The mean age of the participants was 50.0 (SD 10.7) years. More than half of the participants (52.5%) were aged between 35 and 49 years. Almost all participants (98.8%) belonged to Lao-Thai ethnic group.

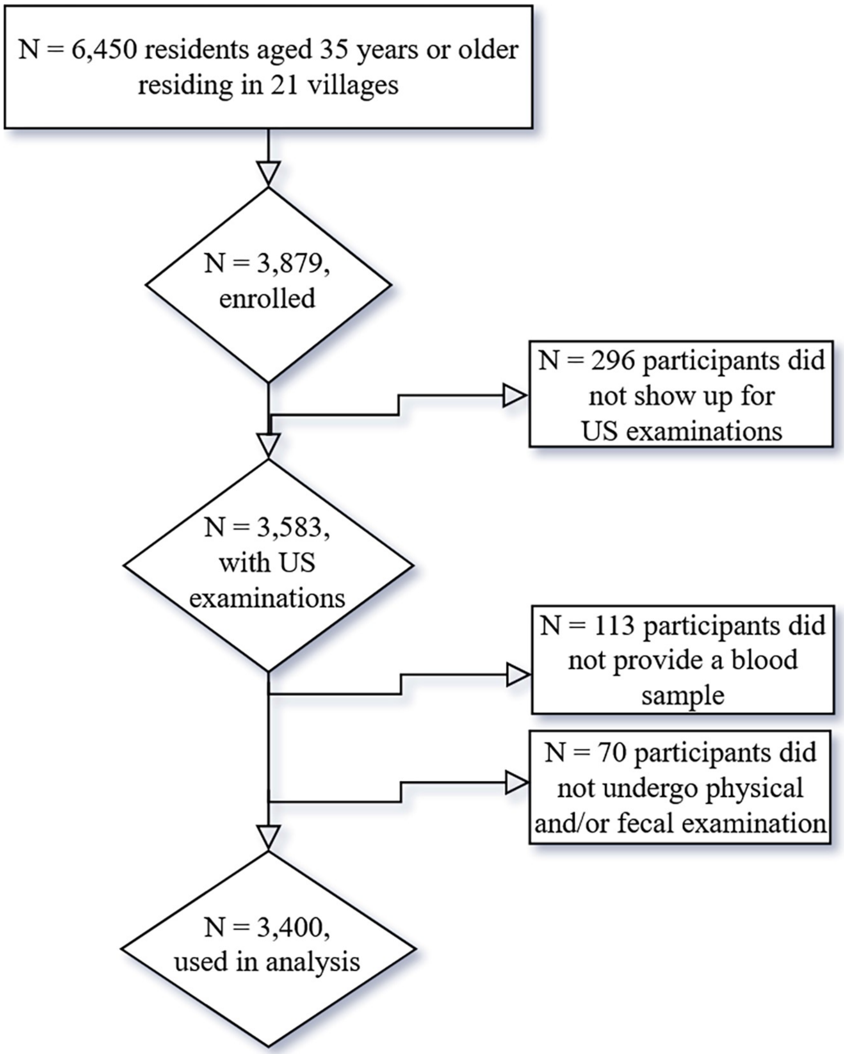

Fig 3. Study diagram.

https://doi.org/10.1371/journal.pntd.0012617.g003

About half (47.2%) of participants had completed primary school education. More than two-thirds (79.4%) were farmers and/or laborer and/or fishermen (Table 1).

3.2. Prevalence of liver morbidity according to demographic characteristics and health risks

Ultrasound examination showed bile duct dilatation in the left liver lobe (LLL), right liver lobe (RLL), and common bile duct (CBD) at rates of 4.5%, 4.6%, and 3.3%, respectively. The shotgun sign was observed in 5.9% of participants and atypical liver masses found in 1.5% of them. When all the criteria for suspected CCA were considered, the overall prevalence of suspected CCA was determined to be 7.2% (95% CI 5.4–9.6) (Table 2). Suspected CCA prevalence in CPS was 7.4% (127 out of 1,711), and in Savannakhet, it was 8.7% (147 out of 1,689). The prevalence of suspected CCA was significantly higher in men (9.6%, 131/1,368) compared to women (7.0%, 143/2,032).

Table 1. Socio-demographic and -economic characteristics of the study participants stratified by suspected cholangiocarcinoma (data presented as row percentage, except in the overall column).

Variables		Overall (N = 3,400)	Suspected CCA		No suspected CCA		Pearson χ^2
		N (%)	N = 274	(%)	N = 3,126	(%)	
**Province**	Champasack	1,711 (50.3)	127	7.4	1,584	92.6	1.88
	Savannakhet	1,689 (49.7)	147	8.7	1,542	91.3	
**Gender**	male	1,368 (40.2)	131	9.6	1,237	90.4	7.11**
	female	2,032 (59.8)	143	7.0	1,889	93.0	
**Age in years**	mean age (±SD)	50.02 (±10.73)	52.1 (±10.7)		49.8 (±10.7)		
**Age groups**	35–49 year	1,784 (52.5)	120	6.7	1,664	93.3	-3.35***
	50–59 year	909 (26.7)	79	8.7	830	91.3	
	≥ 60 year	707 (20.8)	75	10.6	632	89.4	
**Ethnicity**	Lao-Thai	3,357 (98.8)	272	8.1	3,085	91.9	0.58
	Mon-Khmer/Mong-Mein	43 (1.3)	2	4.7	41	95.4	
**Education**	illiterate	591 (17.4)	56	9.5	535	90.5	4.94
	up to primary school	1,606 (47.2)	137	8.5	1,469	91.5	
	secondary school & above	1,203 (35.4)	81	6.7	1,112	93.3	
**Profession**	housewife/elderly/retired	306 (9.0)	27	8.8	279	91.2	3.83
	farmer/laborer/fisherman	2,699 (79.4)	225	8.3	2,474	91.7	
	civil servant/trader	395 (11.6)	22	5.6	373	94.4	
**SES**	poor tertile	1,079 (31.7)	73	6.8	1,006	93.3	5.39
	middle tertile	1,072 (31.5)	84	7.8	988	92.2	
	wealthy tertile	1,249 (36.7)	117	9.4	1,132	90.6	

Note. CCA–cholangiocarcinoma; ±SD: ± standard deviation; SES–social-economic status. Pearson x2 compares the independence between suspected CCA prevalence and each sociodemographic and–economic variables. *p-value < 0.05, **p-value < 0.01, ***p-value < 0.001

https://doi.org/10.1371/journal.pntd.0012617.t001

The prevalence of suspected CCA increased significantly with age, reaching a high of 10.6% among participants 60 years or older. The age-related increase was observed in both genders, with a higher overall mean prevalence among men across all age groups (Fig 4).

Upon examining the prevalence distributions across various risk factors, it appears that a significantly higher prevalence of suspected CCA is likely to be found in the group that exhibited a higher prevalence of OV infection (8.8% vs 2.5%) and in higher OV infection intensity

Table 2. Prevalence of liver morbidities.

Variables	Overall (N = 3,400)	
	N	%
**Bile duct dilatation**		
At left liver lobe (LLL)	153	4.5
At right liver lobe (RLL)	157	4.6
At common bile duct (CBD)	113	3.3
Double-barrel shotgun sign	200	5.9
Any combined	249	7.3
**Liver mass**	52	1.5
**Suspected CCA**	274	7.2 (5.4–9.6)*

*Prevalence rate and 95% confidence interval were derived from logistic regression random-effect model (village as a random effect); N–number of observation.

https://doi.org/10.1371/journal.pntd.0012617.t002

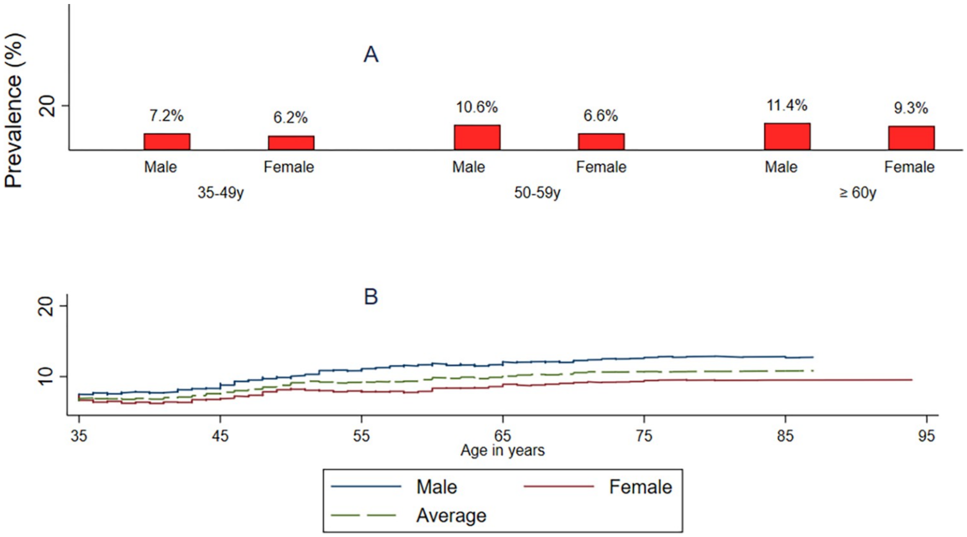

Fig 4. A indicates average prevalence by age group and gender; B indicates average prevalence by ages and gender.

https://doi.org/10.1371/journal.pntd.0012617.g004

group (heavy: 21.9% vs moderate: 11.6% vs non/light: 4.8%). It was significantly higher in individuals with periductal fibrosis (12.3% vs 4.5%), absence/mild fatty liver (8.5% vs 3.9%), gallbladder stones (11.1% vs 7.7%), gallbladder sludge (26.2% vs 6.6%), and a history of cholecystectomy (16.7% vs 7.8%), compared with the group without suspected CCA (Table 3). The distribution of other factors did not differ between those two groups.

3.3. Regression analysis

By comparing the results of the analysis of the bivariate model with different multivariate models, we observed only slight variations of the results (Table 4). In the fully adjusted model, only two predictors showed statistically significant associations with suspected CCA, including OV infection (aOR 3.4, 95% CI 1.7–6.5), and a history of cholecystectomy (aOR 2.7, 95% CI 1.5–4.9). HBV infection (aOR 1.1, 95% CI 0.6–2.2) and regular alcohol consumption (aOR 1.0, 95% CI 0.6–1.8) were associated with a statistically non-significant increased risk of suspected CCA, while T2DM (aOR 0.7, 95% CI 0.4–1.2) and smoking (aOR 0.9, 95% CI 0.7–1.3) were associated with a statistically non-significant decreased risk of suspected CCA (Table 4).

In the sensitivity analysis, all associated variables retained their association with only some changes in the estimated odds ratios. When all individuals with T2DM were excluded, no relevant changes were observed (OV infection: aOR 3.4, 95% CI 1.7–6.8; cholecystectomy: aOR 2.5, 95% CI 1.3–4.9). When individuals with heavy OV infection intensity were excluded, no relevant changes were observed (OV infection: aOR 3.0, 95% CI 1.5–5.8; cholecystectomy: aOR 2.8, 95% CI 1.4–5.3). Exclusion of all individuals who had cholecystectomy done slightly increased the magnitudes of associations between OV infection and the likelihood of having suspected CCA (OV infection: aOR 3.9, 95% CI 1.9–8.1) (Table 5).

4. Discussion

Due to the high burden of helminth infection like OV in Lao PDR, and the significant role of it in causing bile duct disease in humans, we hypothesize that the burden of liver disease in Lao populations is likely underestimated. Furthermore, focusing on OV infection alone might not capture the whole picture. Additionally, the complex interplay of multiple risk factors for

Table 3. OV infection status and other covariates stratified by suspected cholangiocarcinoma status (data presented as row percentage, except in the overall column).

Covariates		Overall (N = 3,400)		Suspected CCA		No suspected CCA		Pearson x2
		N (%)		N = 274	(%)	N = 3,126	(%)	
		Infection						
Opisthorchis viverrini	Negative	397 (11.7)		10	2.5	387	97.5	18.62***
	Positive	3,003 (88.3)		264	8.8	2,739	91.2	
**OV infection intensity**	Non to light intensity	2,130 (62.7)		102	4.8	2,028	95.2	110.48***
	Moderate intensity	1,028 (30.2)		119	11.6	909	88.4	
	Heavy intensity	242 (7.1)		53	21.9	189	78.1	
**Hepatitis B status**	HBsAg-	3,295 (96.9)		264	8.0	3,031	92.0	0.31
	HBsAg+	105 (3.1)		10	9.5	95	90.5	
		Health risk						
**Obesity**	lean	1,873 (55.1)		163	8.7	1,710	91.3	2.33
	not lean	1,527 (44.9)		111	7.3	1,416	91.7	
**T2DM**	no	3,093 (91.0)		256	8.3	2,837	91.7	2.20
	yes	307 (9.0)		18	5.9	289	94.1	
**No. of PZQ cycles**	Never	2,029 (59.7)		177	8.7	1,852	91.3	6.37*
	1–2 times	682 (20.1)		39	5.7	643	94.3	
	≥ 3 times	689 (20.3)		58	8.4	631	91.6	
		Lifestyle						
**Smoking**	Never smokers	2,210 (65.0)		171	7.7	2,039	92.3	0.88
	Past or current	1,190 (35.0)		103	8.7	1,087	91.3	
**Alcohol consumption**	Less	3,181 (93.6)		258	8.1	2,923	91.9	0.18
	≥ 3 times per week	219 (6.4)		16	7.3	203	92.7	
**Ever eaten raw fish**	No	409 (12.0)		31	7.6	378	92.4	0.14
	Yes	2,991 (88.0)		243	8.1	2,748	91.9	
		Co-morbidity/Intermediate symptoms						
**Liver cirrhosis**	Absence	3,343 (98.3)		268	8.0	3,075	92.0	0.48
	Presence	57 (1.7)		6	10.5	51	89.5	
**Periductal fibrosis**	Absence	1,852 (54.5)		84	4.5	1,768	95.5	68.15***
	Presence (all stages)	1,548 (45.5)		190	12.3	1,358	87.7	
**Fatty liver**	Absence or mild	3,045 (89.6)		260	8.5	2,785	91.5	9.06***
	Moderate or severe	355 (10.4)		14	3.9	341	96.1	
**Gallbladder stones**	Absence	3,067 (90.2)		237	7.7	2,830	92.3	4.64*
	Presence	333 (9.8)		37	11.1	296	88.9	
**Gallbladder sludge**	Absence	3,148 (92.6)		208	6.6	2,940	93.4	120.8***
	Presence	252 (7.4)		66	26.2	186	73.8	
**Cholecystectomy**	No	3,310 (97.4)		259	7.8	3,051	92.2	9.24**
	Yes	90 (2.7)		15	16.7	75	83.3	
**Clinical symptoms***	No	3,068 (90.2)		247	8.1	2,821	92.0	0.01
	Yes	332 (9.8)		27	8.1	305	92.0	

Notes. N indicates the number of participants; HBsAg–hepatitis B surface virus antigen; T2DM–type 2 diabetes mellitus; *clinical symptoms mentioned in method section; CCA: cholangiocarcinoma; PZQ: Praziquantel. OV–*Opisthorchis viverrini;* HBsAg–hepatitis B virus surface antigen; light OV infection intensity, EPG between 0 and 999; moderate, EPG between 999 and 10,000; and heavy, EPG>10,000

*p-value < 0.05

**p-value < 0.01

***p-value < 0.001

https://doi.org/10.1371/journal.pntd.0012617.t003

Table 4. Multivariate analysis of covariates associated with suspected cholangiocarcinoma (N = 3,400).

Covariates	Bivariate model	Model adjusted for gender and age	Model adjusted for all socioeconomic & demographic	Model adjusted for co-morbidity	Fully adjusted model
	cOR (95% CI)	aOR (95% CI)	aOR (95% CI)	aOR (95% CI)	aOR (95% CI)
Opisthorchis viverrini					
Negative	Ref	Ref	Ref	Ref	Ref
Positive	3.53 (1.84–6.77)***	3.60 (1.87–6.92)***	3.39 (1.76–6.53)***	3.47 (1.81–6.66)***	3.35 (1.74–6.45)***
**Hepatitis B status**					
HBsAg-	Ref	Ref	Ref	Ref	Ref
HBsAg+	1.07 (0.54–2.12)	1.11 (0.56–2.21)	1.10 (0.55–2.20)	1.08 (0.55–2.15)	1.10 (0.55–2.19)
**T2DM**					
Non-DM	Ref	Ref	Ref	Ref	Ref
DM	0.69 (0.42–1.14)	0.63 (0.38–1.05)	0.65 (0.34–1.08)	0.80 (0.48–1.32)	0.73 (0.43–1.22)
**Post cholecystectomy**					
No	Ref	Ref	Ref	Ref	Ref
Yes	2.46 (1.37–4.41)***	2.60 (1.43–4.74)***	2.70 (1.48–4.93)***	2.66 (1.47–4.83)***	2.67 (1.46–4.89)***
**Smoking**					
Never smokers	Ref	Ref	Ref	Ref	Ref
Past & current	1.18 (0.90–1.55)	0.99 (0.73–1.35)	0.94 (0.69–1.30)	1.14 (0.86–1.51)	0.93 (0.68–1.28)
**Alcohol consumption**					
Less	Ref	Ref	Ref	Ref	Ref
≥ 3 times per week	1.13 (0.66–1.95)	1.01 (0.57–1.77)	1.02 (0.58–1.80)	1.06 (0.61–1.84)	1.03 (0.58–1.82)

Notes. CI: confidence intervals; cOR: crude odds ratio; aOR: adjusted odds ratio; DM diabetes mellitus; HBsAg hepatitis B virus surface antigen; PZQ Praziquantel
*p-value < 0.05
**p-value < 0.01
***p-value < 0.001.

https://doi.org/10.1371/journal.pntd.0012617.t004

hepatobiliary diseases raises the possibility that other factors besides OV infection might also significantly contribute to the disease. To address this hypothesis, we conducted a population-based investigation in southern Lao PDR to assess liver morbidity and its determinants, including both infectious and non-infectious risk factors. Our most important finding revealed an alarmingly high burden of deadly CCA in Lao PDR.

This US screening survey revealed a high overall prevalence of US-derived suspected CCA at 7.2% in the OV-endemic rural southern region of Lao PDR. Males and older individuals showed a higher prevalence, highlighting a critical public health concern for these groups within the already high disease burden. Furthermore, this rate was observed no significant difference in the distribution of suspected CCA between areas that received PZQ treatment and those that did not (7.4% in Champasack vs. 8.7% in Savannakhet), indicating the diminished role of PZQ treatment in the situation of repeated OV infection. Although our study focused on two provinces in southern Lao PDR, by considering the similarity of OV endemic regions characterized by high OV endemicity, shared raw-fish eating habits, and ecological features such as the main Mekong River, common to most southern provinces of Lao PDR, we can speculate that the rate of hepatic morbidity due to suspected CCA might be similarly high in other areas.

Table 5. Sensitivity analysis.

Covariates	Exclude individuals with T2DM	Exclude individuals with heavy OV intensity	Exclude individuals who have undergone cholecystectomy
	aOR (95% CI)	aOR (95% CI)	aOR (95% CI)
	Risk factors		
***Opisthorchis viverrini* infection**			
Negative	Ref	Ref	Ref
Positive	3.41 (1.71–6.80)***	3.00 (1.54–5.78)***	3.91 (1.89–8.10)***
**Hepatitis B status**			
HBsAg-	Ref	Ref	Ref
HBsAg+	1.15 (0.57–2.31)	0.99 (0.46–2.13)	1.13 (0.56–2.26)
**T2DM**			
Non-DM	-	Ref	Ref
DM	-	0.78 (0.45–1.35)	0.71 (0.41–1.22)
**Post cholecystectomy**			
No	Ref	Ref	-
Yes	2.54 (1.33–4.86)***	2.76 (1.44–5.27)***	-
**Smoking**			
Never smokers	Ref	Ref	Ref
Past & current	0.91 (0.66–1.27)	1.01 (0.71–1.43)	0.95 (0.69–1.30)
**Alcohol consumption**			
Less	Ref	Ref	Ref
≥ 3 times per week	0.85 (0.46–1.58)	1.03 (0.54–1.96)	1.00 (0.57–1.77)

Notes. CI: confidence intervals; aOR: adjusted odds ratio; OV *Opisthorchis viverrini;* DM diabetes mellitus; HBsAg hepatitis B virus surface antigen; PZQ-Praziquantel

*p-value < 0.05

**p-value < 0.01

***p-value < 0.001

https://doi.org/10.1371/journal.pntd.0012617.t005

In comparison, two earlier studies conducted in Lao PDR reported lower US-derived suspected CCA rates of only 1.2% [25], and a similar rate documented in a previous US screening from 2007 to 2011 in several Lao PDR provinces, where 9.7% of 6,113 participants exhibited intrahepatic bile duct dilatation [35]. Additionally, data from an ongoing CCA screening and treatment program in Thailand (2015) revealed a prevalence of 5.6%. While not directly comparable due to geographical differences, this study from Thailand identified 2,661 cases of CCA among 47,285 individuals screened via US [8]. The notable discrepancy in suspected CCA rates could be attributed to various factors, including differences in population demographics or OV infection intensities under the population of studies. Furthermore, as the standard practice for CCA screening in Lao PDR has not been available, there might be some discrepancy of diagnostic definition among studies. For instance, in our study, all individuals with bile duct dilatation (> 2mm) were classified as suspected CCA, while other studies might considered using other criteria (e.g., Aye et al. reported a bile duct dilatation of 1.6%, but suspected CCA rate was at 1.2%) [25]. In the study by Kim et al., a suspected case classification based on the numbers of dilated intrahepatic branches termed "dilated intrahepatic bile duct grading" was used [35].

The results of this study focus on the pre-clinical manifestation of CCA examined within the community using ultrasound (US) examination. Extending this into a long-term follow-up study, preliminary results revealed that out of 274 initially suspected CCA cases (from this

baseline US screening), a two-year follow-up study involving further radiological examinations and surgical treatment was conducted. During this period, 174 patients underwent a CT scan at Mahosot Hospital, Vientiane, Lao PDR. Among these individuals, 22 were highly suspicious for CCA based on imaging. Of these confirmed cases, 5 declined surgery, 5 underwent successful surgical procedures, and 12 died during the follow-up period. Importantly, all 5 patients who received surgery had histologically confirmed CCA from the surgical resection samples.

OV infection in this study was strongly associated with suspected CCA, reaffirming its role in pathogenesis. This cause-effect relationship has been extensively documented, particularly in the northeastern region of Thailand, where OV prevalence is moderately high and has been corroborated by both experimental and epidemiological investigations [9–11]. In southern Lao PDR, OV infection rates remain alarmingly elevated. Several key factors contribute to this situation: (i) the persistent consumption of raw or insufficiently cooked fish dishes and a lack of awareness about OV infection [36], akin to observations in northeastern Thailand [37]; (ii) the absence of regular information, education, and communication (IEC) initiatives, including mass drug administrations (MDA) currently limited to Champasack; and (iii) environmental factors conducive to OV's life cycle, such as the Mekong River, various rivers, large man-made reservoirs, poor hygienic practices in rural communities, and the presence of animal reservoirs like dogs and cats [38].

OV infection is not only linked directly to CCA but also contributes to various bile duct morbidities. This is because of the parasite ability to interfere with bile stasis, the ability of the parasite eggs of stones formation, multiple hepatobiliary morbidities are associated including gallbladder sludge, gallbladder stone, hepatomegaly, cholangitis, periductal fibrosis, cholecystitis, and gallstones [33,34]. Our study identified a high prevalence of some of these morbidities, confirming the link between OV infection and morbidities throughout the bile systems. Periductal fibrosis (PDF) was the most prevalent morbidity observed in our study, affecting 45.5% of participants. PDF is a known intermediate step in the pathway to CCA development, caused by chronic irritation and inflammation from long-term OV infection [9,16,17,19,20]. This finding highlights the potential impact of chronic OV infection on our study population.

This study suggest a possible link between cholecystectomy and suspected CCA, however, interpretation of this association is challenging. This is because of a potential false positive result by defining CBD at >10mm, where this threshold might be questionable because the CBD can naturally widen after a cholecystectomy [26,27]. Therefore, the observed association and the interpretation of US screening results for CCA diagnosis require further investigation with additional follow-up and more sensitive techniques. Importantly, in areas with a high prevalence of OV infection, individuals with past OV infection remain at risk for developing CCA regardless of cholecystectomy status. This is supported by evidence from other studies, such as a meta-analysis of mixed study types showing an increased risk of confirmed CCA in patients who underwent cholecystectomy (pooled OR 1.5, 95% CI 1.2–1.9 [39], outside of OV endemic areas). These findings emphasize the need for careful clinical judgment when diagnosing CCA in patients with a history of cholecystectomy, especially in OV-endemic areas. Here, a comprehensive evaluation should consider (i) natural CBD dilatation after cholecystectomy, (ii) potential additional risk from cholecystectomy itself, and (iii) presence of OV infection.

In areas endemic for chronic HBV or HCV infection, such viruses are known as the main risk factors for common liver malignancies, such as HCC [40]. For CCA, there have been some links between viral hepatitis and CCA. For instance, Palmer *et al.*, reported an increased combined OR for both viral HBV infection (OR 5.1, 95% CI 2.9–8.9) and HCV infection (OR 4.8, 95% CI 2.4–9.7) with increased risk for iCCA [41]. Furthermore, a cohort study conducted in Korea also reported HBV infection and an increased risk of CCA (HR 2.7, 95% CI 1.6–4.6), but not for HCV infection [42]. Despite the high prevalence of HBV among Lao adult

population, such as 8.7% in blood adult donors according to a survey conducted in 2008 [13], this study found a lower prevalence of viral infection (<3%) and did not show a significant association with suspected CCA among the studied individuals. Although strong evidence from other studies support the link between HBV infection and CCA, our results suggest that HBV infection does not play a role in the pathogenesis of suspected CCA in Lao PDR.

An alcohol dose-response relationship has been observed elsewhere with the occurrence of CCA (e.g. consuming ≥3 alcoholic drinks/day) [43]. In a meta-analysis by Palmer *et al.*, a combined OR of 2.8 (95% CI, 1.5–5.2) for the risk of iCCA was reported for heavy alcohol consumption (>80 g/day) [41], and, in a cohort study conducted in Korea, heavy alcohol consumption (≥40 g/day) was associated with an increased risk of CCA (HR 1.5, 95% CI 1.3–1.9) [42]. Furthermore, a large meta-analysis of 26 prospective studies revealed that high frequency of alcohol consumption, such as those who consumed five or more drinks a day, was associated with iCCA (HR 2.4, 95% CI 1.5–3.8) [44]. In our study, we did not find a significant association with high alcohol consumption and suspected CCA, likely due to a low prevalence of heavy drinker among our participants.

The association between smoking and CCA has been inconclusive in the literature. Different observational studies have reported no significant associations as example given in a matched case-control study from Thailand, by Poomphakwaen *et al.* reported no significant association (OR 0.9, 95% CI 0.5–2.8) between the two terms [10,14], and, similarly, in a case-control study by Songsorm *et al.*, (aOR 1.2, 95% CI 0.5–2.8) [15]. In contrast, in a Korean cohort study, smoking was associated with an increased risk of CCA showing that those who currently smoke (less than 1 pack/day) had an increased risk for CCA, with a HR of 1.3 (95% CI 1.1–1.5) and those who currently smoke more than 1 pack/day had a HR increase to 1.5 (95% CI 1.1–1.9) [42]. A further example was reported in a meta-analysis of 26 prospective studies revealed a significant association between ever, former, and current smoking and CCA, with a HR of 1.7 (95% CI 1.3–2.1) [44]. In our study, we arrived at similar findings as other observational studies, indicating no associations between smoking and suspected CCA.

While studies from non-OV-endemic areas suggest a link between non-communicable diseases (NCDs) like type 2 diabetes (T2DM) and increased CCA risk (e.g., Korean cohort study: HR 1.4, 95% CI 1.2–1.6 [42]; meta-analysis: combined OR 1.9, 95% CI 1.7–2.1 for iCCA in T2DM patients [41]), and even higher risks in OV endemic areas (e.g., Thai study: 2.4-fold increased risk for CCA with both OV and T2DM [45]), our study did not find a significant association between these conditions. Studies in rodents suggest that liver fat deposition, a factor commonly seen in insulin resistance and T2DM patients, may worsen with OV infection and a high-fat, high-fructose diet (as shown by histological examination) [46]. This finding suggests a possible link between OV infection's inflammatory effects and increased liver fat deposition in these animals. However, more evidence is needed to determine how interactions between T2DM and OV infection might influence the severity of liver disease and potentially contribute to CCA. Despite the lack of association between T2DM and CCA in this study, our previous findings suggest interactions between OV infection and certain NCDs. For example, we observed a lower steatotic liver disease in lean individuals infected with the OV liver fluke [47].

5. Conclusion

In summary, this population-based US screening for suspected CCA conducted in two rural area with high OV prevalence in Lao PDR has unveiled a significant burden of suspected CCA. The primary contributor to this burden is the high OV infection prevalence, highlighting the urgent need for intensified interventions to reduce OV infection and reinfection in these communities. Age and gender disparities in suspected CCA prevalence underscore the importance

of targeted interventions. Beyond OV infection, known for its association with CCA, our study identified notable associated factors such as a history of cholecystectomy, providing valuable insights for early warning signs that require attention in preventive strategies. This research enhances our understanding of hepatobiliary morbidity in high-risk communities and can guide public health initiatives aimed at curbing the incidence of CCA in Lao PDR.

6. Limitation

Although mobile US is a very useful screening tool for suspected CCA, the complexity of CCA diagnostics can lead to misdiagnosis of early-stage tumors, as many liver morbidities can mimic similar conditions. Additionally, US cannot differentiate between liver mass types, such as distinguishing HCC from CCA. It is therefore important that patients with suspicious lesions undergo regular follow-up ultrasound examinations in order to monitor the changes of the bile duct dilatation and, in the event of suspected precancerous lesions, to undergo further diagnostics to confirm the diagnosis. Due to the nature of clustered sampling implemented in this study, the prevalence estimate may not be representative of the entire study population. Furthermore, the use of the Kato-Katz technique in detecting OV eggs might have encountered bias, as the egg morphology of OV is similar to that of other minute intestinal flukes (MIF). Moreover, there were suspicions that mixed species of liver fluke might be present in Lao PDR, such as *Clonorchis sinensis*, which is indistinguishable from OV based solely on egg morphology. The cross-sectional nature of the study does not allow interpretation the direction of associations and the causal inference.

Supporting information

S1 Appendix. distributions of participants.
(DOCX)

S2 Appendix. Multivariate analysis of covariates associated with suspected cholangiocarcinoma among 3,400 study participants (full covariates model).
(DOCX)

S3 Appendix. Sensitivity analysis.
(DOCX)

S4 Appendix. individual's clinical symptoms and liver biochemistry results.
(DOCX)

Acknowledgments

We thank the contribution of staffs from the Lao Tropical and Public Heaths Institute, Vientiane, Lao PDR, the National Center of Mariology, Parasitology and Entomology, Ministry of Health, Vientiane, Lao PDR, The University of Health Science, Vientiane, Lao PDR and the Provincial Health Department of Champasack and Savannakhet. We thank the District Health Office of Khong and Champhone districts for helping in the data collection, Kato-Katz examination, questionnaires administrations, and biospecimens collections. We are grateful for the study participants and authorities at all levels for their supports and contribution to our study.

Author Contributions

Conceptualization: Anousin Homsana, Kerstin Kling, Jan Hattendorf, Daniel Henry Paris, Nicole Probst-Hensch, Somphou Sayasone, Peter Odermatt.

Data curation: Anousin Homsana.

Formal analysis: Anousin Homsana, Jan Hattendorf.

Funding acquisition: Peter Odermatt.

Investigation: Kerstin Kling, Daniel Henry Paris, Nicole Probst-Hensch, Somphou Sayasone, Peter Odermatt.

Methodology: Kerstin Kling, Jan Hattendorf, Savina Vorasane, Daniel Henry Paris, Nicole Probst-Hensch, Somphou Sayasone, Peter Odermatt.

Project administration: Anousin Homsana, Phonesavanh Southisavath, Somphou Sayasone, Peter Odermatt.

Supervision: Somphou Sayasone, Peter Odermatt.

Validation: Anousin Homsana, Phonesavanh Southisavath.

Visualization: Anousin Homsana.

Writing – original draft: Anousin Homsana.

Writing – review & editing: Anousin Homsana, Phonesavanh Southisavath, Kerstin Kling, Jan Hattendorf, Savina Vorasane, Daniel Henry Paris, Nicole Probst-Hensch, Somphou Sayasone, Peter Odermatt.

References

1. Khan SA, Tavolari S, Brandi G. Cholangiocarcinoma: Epidemiology and risk factors. Liver International. 2019; 39:19–31. https://doi.org/10.1111/liv.14095 PMID: 30851228
2. Ghouri YA, Mian I, Blechacz B. Cancer review: cholangiocarcinoma. Journal of carcinogenesis. 2015; 14. https://doi.org/10.4103/1477-3163.151940 PMID: 25788866
3. Bertuccio P, Bosetti C, Levi F, Decarli A, Negri E, La Vecchia C. A comparison of trends in mortality from primary liver cancer and intrahepatic cholangiocarcinoma in Europe. Annals of Oncology. 2013; 24 (6):1667–74. https://doi.org/10.1093/annonc/mds652 PMID: 23378539
4. Shaib Y, El-Serag HB. The epidemiology of cholangiocarcinoma. Semin Liver Dis. 2004; 24(2):115–25. https://doi.org/10.1055/s-2004-828889 PMID: 15192785
5. Sriamporn S, Pisani P, Pipitgool V, Suwanrungruang K, Kamsa-ard S, Parkin DM. Prevalence of Opisthorchis viverrini infection and incidence of cholangiocarcinoma in Khon Kaen, Northeast Thailand. Trop Med Int Health. 2004; 9(5):588–94. https://doi.org/10.1111/j.1365-3156.2004.01234.x PMID: 15117303
6. Calvisi DF, Boulter L, Vaquero J, Saborowski A, Fabris L, Rodrigues PM, et al. Criteria for preclinical models of cholangiocarcinoma: scientific and medical relevance. Nature Reviews Gastroenterology & Hepatology. 2023:1–19. https://doi.org/10.1038/s41575-022-00739-y PMID: 36755084
7. Pattanathien P, Khuntikeo N, Promthet S, Kamsa-Ard S. Survival rate of extrahepatic cholangiocarcinoma patients after surgical treatment in Thailand. Asian Pac J Cancer Prev. 2013; 14(1):321–4. https://doi.org/10.7314/apjcp.2013.14.1.321 PMID: 23534746
8. Khuntikeo N, Chamadol N, Yongvanit P, Loilome W, Namwat N, Sithithaworn P, et al. Cohort profile: cholangiocarcinoma screening and care program (CASCAP). BMC Cancer. 2015; 15:459. https://doi.org/10.1186/s12885-015-1475-7 PMID: 26054405
9. Sripa B, Kaewkes S, Sithithaworn P, Mairiang E, Laha T, Smout M, et al. Liver fluke induces cholangiocarcinoma. PLoS Med. 2007; 4(7):e201. https://doi.org/10.1371/journal.pmed.0040201 PMID: 17622191
10. Poomphakwaen K, Promthet S, Kamsa-Ard S, Vatanasapt P, Chaveepojnkamjorn W, Klaewkla J, et al. Risk factors for cholangiocarcinoma in Khon Kaen, Thailand: a nested case-control study. Asian Pac J Cancer Prev. 2009; 10(2):251–8. PMID: 19537893
11. Manwong M, Songserm N, Promthet S, Matsuo K. Risk factors for cholangiocarcinoma in the lower part of Northeast Thailand: a hospital-based case-control study. Asian Pac J Cancer Prev. 2013; 14 (10):5953–6. https://doi.org/10.7314/apjcp.2013.14.10.5953 PMID: 24289607

12. Alsaleh M, Leftley Z, Barbera TA, Sithithaworn P, Khuntikeo N, Loilome W, et al. Cholangiocarcinoma: a guide for the nonspecialist. Int J Gen Med. 2019; 12:13–23. https://doi.org/10.2147/IJGM.S186854 PMID: 30588065

13. Sitbounlang P, Marchio A, Deharo E, Paboriboune P, Pineau P. The Threat of Multiple Liver Carcinogens in the Population of Laos: A Review. Livers. 2021; 1(1):49–59. https://doi.org/10.3390/livers1010005

14. Intajarurnsan S, Khuntikeo N, Chamadol N, Thinkhamrop B, Promthet S. Factors Associated with Periductal Fibrosis Diagnosed by Ultrasonography Screening among a High Risk Population for Cholangiocarcinoma in Northeast Thailand. Asian Pac J Cancer Prev. 2016; 17(8):4131–6.

15. Songserm N, Promthet S, Sithithaworn P, Pientong C, Ekalaksananan T, Chopjitt P, et al. Risk factors for cholangiocarcinoma in high-risk area of Thailand: role of lifestyle, diet and methylenetetrahydrofolate reductase polymorphisms. Cancer Epidemiol. 2012; 36(2):e89–94. https://doi.org/10.1016/j.canep.2011.11.007 PMID: 22189445

16. Mairiang E, Laha T, Bethony JM, Thinkhamrop B, Kaewkes S, Sithithaworn P, et al. Ultrasonography assessment of hepatobiliary abnormalities in 3359 subjects with Opisthorchis viverrini infection in endemic areas of Thailand. Parasitology international. 2012; 61(1):208–11. https://doi.org/10.1016/j.parint.2011.07.009 PMID: 21771664

17. Andrews RH, Sithithaworn P, Petney TN. Opisthorchis viverrini: an underestimated parasite in world health. Trends Parasitol. 2008; 24(11):497–501. https://doi.org/10.1016/j.pt.2008.08.011 PMID: 18930439

18. Khieu V, Sayasone S, Muth S, Kirinoki M, Laymanivong S, Ohmae H, et al. Elimination of schistosomiasis mekongi from endemic areas in Cambodia and the Lao People's Democratic Republic: Current status and plans. Tropical medicine and infectious disease. 2019; 4(1):30. https://doi.org/10.3390/tropicalmed4010030 PMID: 30736431

19. Sripa B, Brindley PJ, Mulvenna J, Laha T, Smout MJ, Mairiang E, et al. The tumorigenic liver fluke Opisthorchis viverrini—multiple pathways to cancer. Trends Parasitol. 2012; 28(10):395–407. https://doi.org/10.1016/j.pt.2012.07.006 PMID: 22947297

20. Chamadol N, Pairojkul C, Khuntikeo N, Laopaiboon V, Loilome W, Sithithaworn P, et al. Histological confirmation of periductal fibrosis from ultrasound diagnosis in cholangiocarcinoma patients. J Hepatobiliary Pancreat Sci. 2014; 21(5):316–22. https://doi.org/10.1002/jhbp.64 PMID: 24420706

21. Hughes T, O'Connor T, Techasen A, Namwat N, Loilome W, Andrews RH, et al. Opisthorchiasis and cholangiocarcinoma in Southeast Asia: an unresolved problem. Int J Gen Med. 2017; 10:227–37. https://doi.org/10.2147/IJGM.S133292 PMID: 28848361

22. Aye Soukhathammavong P, Vonghachack Y, Hatz C, Akkhavong K, Odermatt P. Suspected cases of cholangiocarcinoma seen in reference hospitals in Lao People's Democratic Republic. Parasitol Int. 2017; 66(4):510–4. https://doi.org/10.1016/j.parint.2016.11.011 PMID: 27965165

23. Mairiang E. Ultrasonographic features of hepatobiliary pathology in opisthorchiasis and opisthorchiasis-associated cholangiocarcinoma. Parasitol Int. 2017; 66(4):378–82. https://doi.org/10.1016/j.parint.2016.12.005 PMID: 27956092

24. Ministry of Health Lao PDR. A diagnosis and treatment guideline for the district hospital in Lao PDR. 2nd ed ed. Vientiane Ministry of Health 2004.

25. Aye Soukhathammavong P, Rajpho V, Phongluxa K, Vonghachack Y, Hattendorf J, Hongvanthong B, et al. Subtle to severe hepatobiliary morbidity in Opisthorchis viverrini endemic settings in southern Laos. Acta Trop. 2015; 141(Pt B):303–9. https://doi.org/10.1016/j.actatropica.2014.09.014 PMID: 25275346

26. Park SM, Kim WS, Bae I-H, Kim JH, Ryu DH, Jang L-C, et al. Common bile duct dilatation after cholecystectomy: a one-year prospective study. Journal of the Korean Surgical Society. 2012; 83(2):97. https://doi.org/10.4174/jkss.2012.83.2.97 PMID: 22880184

27. Atkinson CJ, Lisanti CJ, Schwope RB, Ramsey D, Dinh T, Cochet A, et al. Mild asymptomatic intrahepatic biliary dilation after cholecystectomy, a common incidental variant. Abdominal Radiology. 2017; 42:1408–14. https://doi.org/10.1007/s00261-016-1017-z PMID: 28154908

28. Strauss S, Gavish E, Gottlieb P, Katsnelson L. Interobserver and intraobserver variability in the sonographic assessment of fatty liver. American Journal of Roentgenology. 2007; 189(6):W320–W3. https://doi.org/10.2214/AJR.07.2123 PMID: 18029843

29. Katz N, Chaves A, Pellegrino J. A simple, device for quantita tive stool thick-smear technique in schistosomiasis mansoni. Revista do Instituto de medicina tropical de Sao Paulo. 1972; 14(6):397–400. PMID: 4675644

30. Vonghachack Y, Odermatt P, Taisayyavong K, Phounsavath S, Akkhavong K, Sayasone S. Transmission of Opisthorchis viverrini, Schistosoma mekongi and soil-transmitted helminthes on the Mekong

Islands, Southern Lao PDR. Infect Dis Poverty. 2017; 6(1):131. https://doi.org/10.1186/s40249-017-0343-x PMID: 28866984

31. Sayasone S, Meister I, Andrews JR, Odermatt P, Vonghachack Y, Xayavong S, et al. Efficacy and Safety of Praziquantel Against Light Infections of Opisthorchis viverrini: A Randomized Parallel Single-Blind Dose-Ranging Trial. Clin Infect Dis. 2017; 64(4):451–8. https://doi.org/10.1093/cid/ciw785 PMID: 28174906
32. Xu R, Pan J, Zhou W, Ji G, Dang Y. Recent advances in lean NAFLD. Biomed Pharmacother. 2022; 153:113331. https://doi.org/10.1016/j.biopha.2022.113331 PMID: 35779422
33. Sripa B, Kanla P, Sinawat P, Haswell-Elkins MR. Opisthorchiasis-associated biliary stones: light and scanning electron microscopic study. World Journal of Gastroenterology: WJG. 2004; 10(22):3318.
34. Mairiang E, Haswell-Elkins MR, Mairiang P, Sithithaworn P, Elkins DB. Reversal of biliary tract abnormalities associated with Opisthorchis viverrini infection following praziquantel treatment. Transactions of the Royal Society of Tropical Medicine and Hygiene. 1993; 87(2):194–7. https://doi.org/10.1016/0035-9203(93)90489-d PMID: 8337727
35. Kim JY, Yong TS, Rim HJ, Chai JY, Min DY, Eom KS, et al. Ultrasonographic investigation of cholangiocarcinoma in Lao PDR. Acta Trop. 2018; 182:128–34. https://doi.org/10.1016/j.actatropica.2018.02.031 PMID: 29486176
36. Yoon HJ, Ki M, Eom K, Yong TS, Chai JY, Min DY, et al. Risk factors for Opisthorchis viverrini and minute intestinal fluke infections in Lao PDR, 2009–2011. Am J Trop Med Hyg. 2014; 91(2):384–8. https://doi.org/10.4269/ajtmh.13-0596 PMID: 24980495
37. Saengsawang P, Promthet S, Bradshaw P. Reinfection by Opisthorchis Viverrini after Treatment with Praziquantel. Asian Pac J Cancer Prev. 2016; 17(2):857–62. https://doi.org/10.7314/apjcp.2016.17.2.857 PMID: 26925692
38. Sayasone S, Odermatt P, Phoumindr N, Vongsaravane X, Sensombath V, Phetsouvanh R, et al. Epidemiology of Opisthorchis viverrini in a rural district of southern Lao PDR. Trans R Soc Trop Med Hyg. 2007; 101(1):40–7. https://doi.org/10.1016/j.trstmh.2006.02.018 PMID: 16828134
39. Xiong J, Wang Y, Huang H, Bian J, Wang A, Long J, et al. Systematic review and meta-analysis: cholecystectomy and the risk of cholangiocarcinoma. Oncotarget. 2017; 8(35):59648–57. https://doi.org/10.18632/oncotarget.19570 PMID: 28938668
40. McGlynn KA, Tarone RE, El-Serag HB. A comparison of trends in the incidence of hepatocellular carcinoma and intrahepatic cholangiocarcinoma in the United States. Cancer Epidemiol Biomarkers Prev. 2006; 15(6):1198–203. https://doi.org/10.1158/1055-9965.EPI-05-0811 PMID: 16775181
41. Palmer WC, Patel T. Are common factors involved in the pathogenesis of primary liver cancers? A meta-analysis of risk factors for intrahepatic cholangiocarcinoma. J Hepatol. 2012; 57(1):69–76. https://doi.org/10.1016/j.jhep.2012.02.022 PMID: 22420979
42. Cho IR, Yi SW, Choi JS, Yi JJ. Comparison of Risk Factors for Cholangiocarcinoma and Hepatocellular Carcinoma: A Prospective Cohort Study in Korean Adults. Cancers (Basel). 2022; 14(7). https://doi.org/10.3390/cancers14071709 PMID: 35406481
43. Turati F, Galeone C, Rota M, Pelucchi C, Negri E, Bagnardi V, et al. Alcohol and liver cancer: a systematic review and meta-analysis of prospective studies. Ann Oncol. 2014; 25(8):1526–35. https://doi.org/10.1093/annonc/mdu020 PMID: 24631946
44. McGee EE, Jackson SS, Petrick JL, Van Dyke AL, Adami HO, Albanes D, et al. Smoking, Alcohol, and Biliary Tract Cancer Risk: A Pooling Project of 26 Prospective Studies. J Natl Cancer Inst. 2019; 111(12):1263–78. https://doi.org/10.1093/jnci/djz103 PMID: 31127946
45. Thinkhamrop K, Khuntikeo N, Laohasiriwong W, Chupanit P, Kelly M, Suwannatrai AT. Association of comorbidity between Opisthorchis viverrini infection and diabetes mellitus in the development of cholangiocarcinoma among a high-risk population, northeastern Thailand. PLoS Negl Trop Dis. 2021; 15(9):e0009741. https://doi.org/10.1371/journal.pntd.0009741 PMID: 34543283
46. Chaidee A, Onsurathum S, Intuyod K, Haonon O, Pannangpetch P, Pongchaiyakul C, et al. Opisthorchis viverrini Infection Augments the Severity of Nonalcoholic Fatty Liver Disease in High-Fat/High-Fructose Diet-Fed Hamsters. Am J Trop Med Hyg. 2019; 101(5):1161–9. https://doi.org/10.4269/ajtmh.19-0442 PMID: 31482785
47. Homsana A, Southisavath P, Kling K, Hattendorf J, Vorasane S, Paris DH, et al. Steatotic liver disease among lean and non-lean individuals in Southern Lao PDR: a cross-sectional study of risk factors. Annals of Medicine. 2024; 56(1):2329133. https://doi.org/10.1080/07853890.2024.2329133 PMID: 38502916
