# Supplementary material for: Periductal Fibrosis and Cholangiocarcinoma-Related Outcomes in Liver Fluke-Endemic Regions: A Systematic Review and Meta-Analysis
Source: Med Sci (Basel). 2026 Jul 9;14(3):380. doi: 10.3390/medsci14030380 (PMC13414246; doi:10.3390/medsci14030380)
Supplement: Supplementary file 1 [file medsci-14-00380-s001.zip › included papers/Mairiang et al., 2012.pdf]

NIH Public Access
Author Manuscript
Parasitol Int. Author manuscript; available in PMC 2014 July 14.

Published in final edited form as:
Parasitol Int. 2012 March ; 61(1): 208–211. doi:10.1016/j.parint.2011.07.009.

Ultrasonography assessment of hepatobiliary abnormalities in 3,359 subjects with *Opisthorchis viverrini* infection in endemic areas of Thailand

Eimorn Mairiang[a], **Thewarach Laha**[b], **Jeffrey M. Bethony**[c], **Bandit Thinkhamrop**[d], **Sasithorn Kaewkes**[b,g], **Paiboon Sithithaworn**[b,g], **Smarn Tesana**[b], **Alex Loukas**[e], **Paul J. Brindley**[c], and **Banchob Sripa**[f,g,*]

[a]Department of Radiology, Faculty of Medicine, Khon Kaen University, Khon Kaen, 40002, Thailand [b]Department of Parasitology, Faculty of Medicine, Khon Kaen University, Khon Kaen, 40002, Thailand [c]Department of Microbiology, Immunology and Tropical Medicine, George Washington University, Washington, DC 20037, USA [d]Department of Biostatistics and Demography, Faculty of Public Health, Khon Kaen University, Khon Kaen, 40002, Thailand [e]Queensland Tropical Health Alliance, James Cook University, Cairns, QLD 4878, Australia [f]Department of Pathology, Faculty of Medicine, Khon Kaen University, Khon Kaen, 40002, Thailand [g]Liver Fluke and, Cholangiocarcinoma Research Center, Faculty of Medicine, Khon Kaen University, Khon Kaen, 40002, Thailand

Abstract

A cross sectional study on hepatobiliary abnormalities in opisthorchiasis was performed in 8,936 males and females aged from 20 to 60 years from 90 villages of Khon Kaen province, Northeast Thailand. All were stool-examined for *Opisthorchis viverrini* infection by standard quantitative formalin/ethyl acetate concentration technique. Of these, 3,359 participants with stool egg positive were underwent ultrasonography of the upper abdomen. The hepatobiliary abnormalities detected by ultrasound are described here. This study found a significantly higher frequency of advanced periductal fibrosis in persons with chronic opisthorchiasis (23.6%), particularly in males. Risks of the fibrosis included intensity of infection, and age younger than 30 years. Height of left lobe of the liver, cross-section of the gallbladder dimensions post fatty meal, sludge, and, interestingly, intrahepatic duct stones were significantly associated with the advanced periductal fibrosis. Eleven suspected cholangiocarcinoma (CCA) cases were observed. This study emphasizes the current status of high *O. viverrini* infection rate and the existence of hepatobiliary abnormalities including suspected CCA in opisthorchiasis endemic areas of Thailand.

© 2011 Elsevier Ireland Ltd. All rights reserved.

*Correspondence author: Banchob Sripa, Tropical Disease Research Laboratory (TDR), Department of Pathology, Faculty of Medicine, Khon Kaen University, Khon Kaen 40002, Thailand; Tel: +66 43 363113 Fax: +66 43 202 024; banchob@kku.ac.th.

Publisher's Disclaimer: This is a PDF file of an unedited manuscript that has been accepted for publication. As a service to our customers we are providing this early version of the manuscript. The manuscript will undergo copyediting, typesetting, and review of the resulting proof before it is published in its final citable form. Please note that during the production process errors may be discovered which could affect the content, and all legal disclaimers that apply to the journal pertain.

NIH-PA Author Manuscript NIH-PA Author Manuscript NIH-PA Author Manuscript

NIH-PA Author Manuscript NIH-PA Author Manuscript NIH-PA Author Manuscript

Keywords

ultrasonography; opisthorchiasis; hepatobiliary abnormalities; cholangiocarcinoma; *Opisthorchis viverrini*

1. Introduction

The carcinogenic liver fluke, *Opisthorchis viverrini* remains an important public health problem in many parts of Southeast Asia, particulary in Northeast Thailand [1]. Recent reports suggested that 6–8 million individuals infected with *O. viverrini* in Thailand [2,3]. The infection is associated with hepatobiliary diseases including hepatomegaly, cholangitis, periductal fibrosis, cholecystitis, gallstones, and is a major aetiological agent of the bile duct cancer, cholangiocarcinoma (CCA) [4]. The liver fluke endemic area of Khon Kaen, Thailand has reported the highest incidence of the liver cancer in the world [5–7].

Previous community-based ultrasound studies in this opisthorchiasis endemic region of Khon Kaen and neighboring provinces showed a wide range of hepatobiliary abnormalities including periductal fibrosis, cholecystitis, gallstones, and suspected CCA [8–10]. After large scale praziquantel treatment for opisthorchiasis in Khon Kaen province and other parts of northeast Thailand for decades [2], still we frequently encounter the hepatobiliary abnormalities and CCA associated with *O. viverrini* infection. Therefore, large-scale community-based cross-sectional study was performed in this area during 2007 to 2010 to elucidate the current status of *O. viverrini* infection and associated hepatobiliary abnormalities including CCA.

2. Materials and methods

2.1 Subjects

This study is a part of a community-based case control study on the pathogenesis of liver fluke induced cancer in Thailand [11]. The study was approved by Khon Kaen University Ethics Committee (reference number HE4880528) and the Institutional Review Board of the George Washington University, DC (GWUMC IRB#020864).

2.2 Recruitment

Recruitment for the study took place from August 2007 to October 2010. Overall, 8,936 individuals from 90 villages of four districts (Ban Had, Ban Pai, Chonabot, Muncha Khiri) of Khon Kaen province, aged between 20 and 60 years old, were screened for *O. viverrini* infection by a quantitative formalin/ethyl acetate concentration technique [11]. There were 3,640 *O. viverrini* egg positive participants (41.5%), of which 3,512 (41.54%) reported previous praziquantel treatment. Among the egg positive individuals, 3,359 participants agreed to have ultrasonography of the upper abdomen. Details of this investigation were described previously [11]. Age and sex distribution as well as other data of the volunteers who underwent ultrasonography are shown in Table 1.

NIH-PA Author Manuscript

2.3 Ultrasonography

A total of 3,359 subjects were examined by abdominal ultrasonography using a mobile high-resolution ultrasound machine (GE model LOGIQ Book XP). Hepatobiliary abnormalities (HBA) including periportal fibrosis in liver parenchyma, gallbladder wall, gallbladder size, sludge, suspected CCA (dilated intra or extrahepatic bile duct and/or liver mass) were graded and recorded as described [8, 11, 12]. For the study of gallbladder function (contractility), the gallbladder was measured 30 minutes after consumption of a fatty meal [8]. Periductal fibrosis is defined by periportal echoes (more than 3 mm) around second order intrahepatic bile ducts. Grading of the fibrosis included grade 0 = normal, grade I = periportal echoes less than 2 segments of the liver, grade II = 2–3 segments, and grade III = more than 3 segments [11]. Designation of advanced periductal fibrosis (APF) was made if the ultrasound grading was grade II and above. Disease of other organs in the abdomen and pelvis, if detected, were also recorded. Renal stones were recorded separately because renal stone disease also has a high prevalence in this region. A radiologist who performed ultrasonography was unaware of parasitic infection status of the subjects. Representative ultrasound images of different liver diseases are illustrated in Figure 1.

2.4 Referrals

Participants who were *O. viverrini* egg positive (upon stool examination) and had abnormal ultrasound findings were informed and advised to undertake further investigation or treatment according to standard health care of the Thai Ministry of Public Health. Diseases of other organs were also informed and referred for treatment.

2.5 Statistical analysis

Demographic and baseline variables were described. Percent distributions were presented for all categorical variables. For continuous variables, mean (± standard deviation) and median (minimum: maximum) were used. The 95% confidence intervals (95% CI) were estimated for the prevalence of advanced periductal fibrosis. These estimation methods were based on exact binomial distribution. Relationship between selected demographic factors and various ultrasonographic findings on advanced periductal fibrosis was investigated. Multiple logistic regression was used to obtain odds ratios (ORs) and their 95% CIs. Odds ratios were estimated as unadjusted and adjusted for effect of sex, age, and intensity of infection.

All analyses were performed using STATA version 10 (College Station, TX). Significant level was set as 0.05 and all statistical tests were two-sided.

3. Results

Among a total of 3,359 subjects who had *O. viverrini* egg positive in the stool samples and undergone ultrasonography, 50.1% were male. The mean age of all subjects was 46.1± 8.4 years old, and their median intensity of *O. viverrini* infection was 63 eggs per gram (Table 1). Other selected ultrasonographic findings included 64 (1.9%) kidney stone, 182 (5.4%) gallstone, 355 (10.6%) fatty liver, 95 (2.8%) sludge, and 7 (0.2%) intrahepatic duct stone. There were 12 (7 males and 5 females) cases of suspected CCA. Two of these (only) agreed

NIH-PA Author Manuscript

NIH-PA Author Manuscript

NIH-PA Author Manuscript

to a computerized tomography (CT scan) at Khon Kaen University Hospital. Of these CT scanned individuals, one case was confirmed diagnosis of CCA but refused an operation, another was benign hemangioma. Four other suspected CCA cases died during the follow up period.

This report mainly focuses on benign hepatobiliary diseases with particular emphasis on advanced periductal fibrosis, i.e. high grade periportal echoes. Table 2 summarizes the relationship between sex, age, and intensity of infection on advanced periductal fibrosis. Of 3,359 subjects, 793 subjects had positive findings of advanced periductal fibrosis. Thus the prevalence of the fibrosis was 23.6% (95% CI: 22.2 to 25.0). Factors that were statistically significantly associated with advanced periductal fibrosis included sex (P<0.001), age (P <0.001), intensity of infection (P <0.001), height of left lobe of the liver (P <0.001), cross-section of the gallbladder dimensions "pre" minus "post" fatty meal (P <0.001), and sludge (P <0.001). Interestingly, intrahepatic duct stone was also associated with the advanced periductal fibrosis (P =0.009). Females were 40% less likely to have advanced periductal fibrosis than males (P = 0.001). Risk of advanced periductal fibrosis was increased with increasing intensity of *O. viverrini* infection. However, the risk in the older age group was about half that seen in participants younger than 30 years old (Table 2).

4. Discussion

Opisthorchiasis control in Thailand started in 1950. Notably, a large scale Opisthorchiasis Control Program was conducted between 1983 and 2001 in Northeast Thailand [2, 13]. A country wide prevalence of *O. viverrini* infection in Thailand of 63% in the early years after records were begun to be maintained. However, by the year 2001 this had declined to 9.4% for the entire country, and to 15.7 % for Northeast Thailand. Remarkably, despite intensive control targeting *O. viverrini* infection in the Thai population over the past three decades, the present report revealed that the prevalence of *O. viverrini* and the associated hepatobiliary abnormalities remain alarmingly high.

Although there is no standard guideline, several ultrasonographic features of opisthorchiasis including hepatomegaly, periductal fibrosis, gallstones, gallbladder sludge, intrahepatic duct stones and poor functioning gall bladder have been described over the past 20 years or so [8–10]. Periductal fibrosis is a prominent sonographic finding in chronic opisthorchiasis. This is similar to pattern B (starry sky) in schistosomiasis (diffuse echogenic foci around portal and subsegmental branches) [14] and periportal echoes in clonorchiasis [15]. This ultrasonographic characteristic is different from other parenchymal liver diseases. Acute viral hepatitis can cause increased echogenicity of the portal vein due to diffuse decreased parenchymal echogenicity [16]. Sonographic findings in alcoholic liver disease range from diffusely increased echoes (fatty liver) to marked heterogeneous echoes with nodular liver surface (cirrhosis), again quite different from opisthorchiasis [17].

Here advanced periductal fibrosis was noted in 785 subjects (23.6%). The prevalence of APF was significantly higher in male (27.9%) than in female (18.9%). Risk of the fibrosis was increased according to increasing intensity of *O. viverrini* infection. Males are known to exhibit higher intensity of infection than females [18]. However, younger people (less than

NIH-PA Author Manuscript

NIH-PA Author Manuscript

NIH-PA Author Manuscript

30 years) showed higher risk of periductal fibrosis than the older participants in this study. This might relate to active innate immunity inducing more inflammation in the younger as compared to older cases [19].

A total of 182 cases of gallstones were observed among 3,359 examinees (5.4%), 4.5% among males and 5.6% among females. These findings are quite similar to our previous report [10]. The gallbladder sludge and cross section of the gallbladder “pre” minus “post” fatty meal showed a significant relationship with periductal fibrosis. Poor gallbladder function and periductal fibrosis cause functional bile stasis and formation of sludge [11]. Moreover, bile stagnation may increase exposure of the biliary epithelium to hazardous and carcinogenic components of bile, leading to carcinogenesis [20, 21].

There were seven cases of intrahepatic duct stones (0.21%) in the present study. Six of these individuals were male and all had moderate periductal fibrosis; the female case did not have periductal fibrosis. In our previous report [10], prevalence of the intrahepatic duct stones was only 0.06%. Thus the present study reports a prevalence of intrahepatic duct stones at 30 times rate than reported the past. Repeated infections and repeated anti-fluke medication treatments may produce dead worms and debris rendering stone forming, since pigmented stones in opisthorchiasis endemic areas often contain *O. viverrini* eggs in the nidus of the stones [22]. This hypothesis warrants further investigation.

Hospital and community studies have described hepatomegaly as the main pathologic feature of opisthorchiasis [23, 24]. This study confirms such relationship and also found that the liver size, mainly affecting the left lobe of the liver, was associated with periductal fibrosis. The mechanism(s) by which the liver fluke infection induces hepatomegaly is still unclear.

Renal stone disease is prevalent in the northeastern provinces of Thailand with the prevalence raging from 0.4 –8.4% [25, 26]. Our present study reported 1.9% in 90 opisthorchiasis endemic villages and showed no association with advanced periductal fibrosis of the liver. However, there is a report of opisthorchiasis-associated nephropathy in an animal model [27]. The association between *Opisthorchis* infection and renal disease is still to be studied.

In conclusion, this study shows a high prevalence of *O. viverrini* and its associated hepatobiliary abnormalities as detected by ultrasonography in people in endemic areas of Northeast Thailand. The prominent sonographic characteristic of opisthorchiasis is periductal fibrosis and it was significantly related to intensity of infection and in younger (<30 years old) people. The height of left lobe of the liver, the cross-section of the gallbladder dimensions following a fatty meal, sludge, and, interestingly, intrahepatic duct stones were significantly associated with the advanced periductal fibrosis.

Acknowledgments

This work was supported by the National Institute of Allergy and Infectious Disease-NIH (award number UO1A1065871) and the Higher Education Research Promotion and National Research University Project of Thailand, Office of the Higher Education Commission, through the Health Cluster (SHeP-GMS), Khon Kaen University. The content is solely the responsibility of the authors and does not necessarily represent the official

NIH-PA Author Manuscript

NIH-PA Author Manuscript

NIH-PA Author Manuscript

views of the NIAID or the NIH. We thank the volunteers who participated in this study. We also thank our regulatory affairs and data management colleagues including Preeyaporn Plaimee, Sangduan Wannachart, Sombat Thinkhamrop and our dedicated laboratory and field technicians.

References

1. Sripa B, Kaewkes S, Intapan PM, Maleewong W, Brindley PJ. Food-borne trematodiases in Southeast Asia: epidemiology, pathology, clinical manifestation and control. Adv Parasitol. 2010; 72C:305–50. [PubMed: 20624536]
2. Jongsuksuntigul P, Imsomboon T. Opisthorchiasis control in Thailand. Acta Trop. 2003; 88:229–32. [PubMed: 14611877]
3. Andrews RH, Sithithaworn P, Petney TN. *Opisthorchis viverrini*: an underestimated parasite in world health. Trends Parasitol. 2008; 24:497–501. [PubMed: 18930439]
4. Sripa B, Kaewkes S, Sithithaworn P, Mairiang E, Laha T, Smout M, et al. Liver fluke induces cholangiocarcinoma. PLoS Med. 2007; 4:e201. [PubMed: 17622191]
5. Vatanasapt V, Uttaravichien T, Mairiang EO, Pairojkul C, Chartbanchachai W, Haswell-Elkins M. Cholangiocarcinoma in north-east Thailand. Lancet. 1990; 335:116–7. [PubMed: 1967406]
6. Sripa B, Pairojkul C. Cholangiocarcinoma: lessons from Thailand. Curr Opin Gastroenterol. 2008; 24:349–56. [PubMed: 18408464]
7. Shin HR, Oh JK, Masuyer E, Curado MP, Bouvard V, Fang Y, et al. Comparison of incidence of intrahepatic and extrahepatic cholangiocarcinoma - Focus on East and Southeastern Asia. Asian Pac J Cancer Prev. 2010; 11:1159–66. [PubMed: 21198257]
8. Mairiang E, Elkins DB, Mairiang P, Chaiyakum J, Chamadol N, Loapaiboon V, et al. Relationship between intensity of *Opisthorchis viverrini* infection and hepatobiliary disease detected by ultrasonography. J. Gastroenterol. Hepatol. 1992; 7:17–21.
9. Haswell-Elkins MR, Mairiang E, Mairiang P, Chaiyakum J, Chamadol N, Loapaiboon V, et al. Cross-sectional study of *Opisthorchis viverrini* infection and cholangiocarcinoma in communities within a high-risk area in northeast Thailand. Int. J Cancer. 1994; 59:505–9.
10. Elkins DB, Mairiang E, Sithithaworn P, Mairiang P, Chaiyakum J, Chamadol N, et al. Cross-sectional patterns of hepatobiliary abnormalities and possible precursor conditions of cholangiocarcinoma associated with *Opisthorchis viverrini* infection in humans. Am J Trop Med Hyg. 1996; 55:295–301. [PubMed: 8842118]
11. Sripa B, Mairiang E, Thinkhamrop B, Laha T, Kaewkes S, Sithithaworn P, et al. Advanced periductal fibrosis from infection with the carcinogenic human liver fluke *Opisthorchis viverrini* correlates with elevated levels of interleukin-6. Hepatology. 2009; 50:1273–81. [PubMed: 19676135]
12. Mairiang E, Haswell-Elkins MR, Mairiang P, Sithithaworn P, Elkins DB. Reversal of biliary tract abnormalities associated with *Opisthorchis viverrini* infection following praziquantel treatment. Trans R Soc Trop Med Hyg. 1993; 87:194–7. [PubMed: 8337727]
13. Sripa B, Bethony JM, Sithithaworn P, Kaewkes S, Mairiang E, Loukas A, et al. Opisthorchiasis and *Opisthorchis*-associated cholangiocarcinoma in Thailand and Laos. Acta Trop. 2010 Jul 23. Epub ahead of print.
14. Niamey Working Group. Ultrasound in schistosomiasis. A practical guide to the standardized use of ultrasonography for the assessment of schistosomiasis-related morbidity. World Health Organization/TDR/SCH/ULTRASON/document; Geneva, Switzerland: 2000.
15. Lim JH, Mairiang E, Ahn GH. Biliary parasitic diseases including clonorchiasis, opisthorchiasis and fascioliasis. Abdominal Imaging. 2008; 33:157–65. [PubMed: 17934771]
16. Kurtz AB, Rubin CS, Cooper HS, Nisenbaum HL, Cole-Beuglet C, Medoff J, et al. Ultrasound findings in hepatitis. Radiology. 1980; 136:717–23. [PubMed: 7403553]
17. Needleman L, Kurtz AB, Rifkin MD, Cooper HS, Pasto ME, Goldberg BB. Sonography of diffuse benign liver disease: accuracy of pattern recognition and grading. AJR. 1986; 146:1011–5. [PubMed: 3515875]
18. Sithithaworn P, Haswell-Elkins M. Epidemiology of *Opisthorchis viverrini*. Acta Trop. 2003; 88:187–94. [PubMed: 14611873]

NIH-PA Author Manuscript NIH-PA Author Manuscript NIH-PA Author Manuscript

19. Solana R, Pawelec G, Tarazona R. Aging and innate immunity. Immunity. 2006; 24:491–4. [PubMed: 16713963]
20. Ross RK, Hartnett NM, Bernstein L, Henderson BE. Epidemiology of adenocarcinomas of the small intestine: is bile a small bowel carcinogen? Br J Cancer. 1991; 63:143–5. [PubMed: 1989654]
21. Schulze J, Richter E, Binder U, Zwickenpflug W. Biliary excretion of 4-(methylnitrosamino)-1-(3-pyridyl)-1-butanone in the rat. Carcinogenesis. 1992; 13:1961–5. [PubMed: 1423863]
22. Sripa B, Kanla P, Sinawat P, Haswell-Elkins MR. Opisthorchiasis-associated biliary stones: light and scanning electron microscopic study. World J Gastroenterol. 2004; 22:3318–21. [PubMed: 15484308]
23. Koompirochana C, Sonakul D, Chinda K, Stitnimankarn T. Opisthorchiasis: a clinicopathologic study of 154 autopsy cases. Southeast Asian J Trop Med Public Health. 1978; 9:60–4. [PubMed: 212836]
24. Viranuvatti V, Stitnimankarn T. Liver fluke infection and infestation in Southeast Asia. Prog Liver Dis. 1972; 4:537–47. [PubMed: 4265700]
25. Sriboonlue P, Prasongwatana V, Chata K, Tungsanga K. Prevalence of upper urinary tract stone disease in a rural community of north-eastern Thailand. Br J Urol. 1992; 69:240–4. [PubMed: 1568096]
26. Yanagawa M, Kawamura J, Onishi T, Soga N, Kameda K, Sriboonlue P, et al. Incidence of urolithiasis in northeast Thailand. Int J Urol. 1997; 4:537–40. [PubMed: 9477179]
27. Boonpucknavig S, Boonpucknavig V, Tanvanich S, Doungchawee G, Thamavit W. Development of immune-complex glomerulonephritis and amyloidosis in Syrian golden hamsters infected with *Opisthorchis viverrini*. J Med Assoc Thai. 1992; 75(Suppl 1):7–19. [PubMed: 1402485]

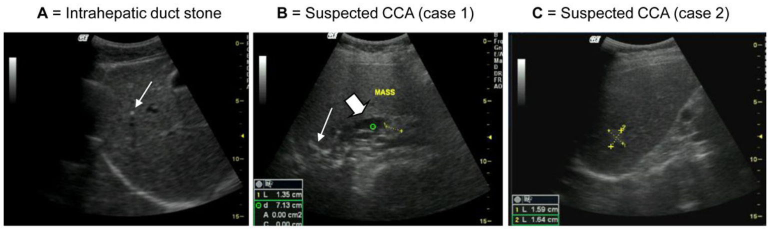

Fig. 1.

Representative abdominal ultrasonography of opisthorchiasis and suspected CCA patients in endemic areas. A= Intrahepatic duct stone (arrow); B = Suspected CCA case 1, ultrasound shows a mass (cursor), calcification (arrow) and dilated intrahepatic duct (large arrow); C = Suspected CCA case 2, ultrasound shows liver mass in segment 7 (cursors)

NIH-PA Author Manuscript NIH-PA Author Manuscript NIH-PA Author Manuscript

NIH-PA Author Manuscript

NIH-PA Author Manuscript

NIH-PA Author Manuscript

Table 1

Characteristics of study participants from Khon Kaen province, Thailand who were *Opisthorchis viverrini* positive and underwent ultrasonographic examination, presented as number and percent (unless specified otherwise).

Characteristics		Total subjects Percent
Sex		
Male		1,683 (50.1)
Female		1,676 (49.9)
Age (years)		
20–29		153 (4.6)
30–39		686 (20.4)
40–49		1,331 (39.6)
50+		1,189 (35.4)
Mean (SD)		46.1 (8.4)
Median (Minimum : Maximum)		47.0 (20.0 : 60.0)
Intensity of infection		
Light	<500 epg	2,955 (88.0)
Medium	501–999 epg	195 (5.8)
Heavy	1000–1999 epg	132 (3.9)
Very Heavy	>2000	77 (2.3)
Mean (SD)		283.3 (950.5)
Median (Minimum : Maximum)		63.0 (2.0 : 23320.0)
Height of left lobe of the liver (cm)		
Mean (SD)		5.5 (1.0)
Median (Minimum : Maximum)		5.5 (2.7 : 10.0)
Gall bladder dimensions “Pre” minus “Post” fatty meal (cm)		
a) Length, mean (SD)		1.55 (1.13)
b) Width, mean (SD)		0.68 (0.53)
c) Cross-section, mean (SD)		0.59 (0.48)
Liver parenchyma		
a) Grade 0		2,566 (76.4)
b) Grade I		0 (0)
c) Grade II		742 (22.1)
c) Grade III		49 (1.5)
d) Dilated bile duct		1 (0.03)
e) Suspected CCA		12 (0.35)
Kidney		
a) Normal		3,239 (96.4)
b) Cortical cyst		21 (0.6)
c) Stone		64 (1.9)
Other ultrasonography findings		
a) Gall stone		182 (5.4)

NIH-PA Author Manuscript

NIH-PA Author Manuscript

NIH-PA Author Manuscript

Characteristics	Total subjects Percent
b) Fatty liver	355 (10.6)
c) Cirrhosis	10 (0.3)
d) Sludge	95 (2.8)
e) Intra hepatic duct stone	7 (0.2)

Epg = eggs per gram (*Opisthorchis viverrini* eggs per gram of feces); SD = standard deviation; cm = centimeters

NIH-PA Author Manuscript
NIH-PA Author Manuscript
NIH-PA Author Manuscript

Table 2

Relationship between sex, age, and intensity of *Opisthorchis viverrini* infection on advanced periductal fibrosis status as determined by ultrasonography.

Characteristics		Number	APF (%)	OR	OR*		
				Unadjusted	Adjusted	95%CI	*p*-value
Sex							
Male		1,683	28.3	1			
Female		1,676	19.0	0.60	0.61	0.52 to 0.72	0.001
Age (years)							
20–29		153	37.9	1			
30–39		686	24.2	0.52	0.54	0.37 to 0.72	<0.001
40–49		1,331	22.5	0.47	0.47	0.33 to 0.67	<0.001
50+		1,189	22.7	0.48	0.47	0.33 to 0.68	<0.001
Intensity of infection (epg)							
Light	<500	2,955	22.2	1			
Medium	501–999	195	29.7	1.15	1.50	1.08 to 2.06	0.014
Heavy	1000–1999	132	35.6	1.94	1.81	1.25 to 2.63	0.002
Very Heavy	>2000	77	42.9	2.63	2.54	1.60 to 4.04	<0.001
Height of left lobe of the liver (every 1 cm)		NA	NA	0.57	0.48	0.52 to 0.62	<0.001
Gall bladder dimensions “Pre” minus “Post” fatty meal							
a) Length (every 1 cm)		NA	NA	0.94	0.93	0.86 to 1.01	0.067
b) Width (every 1 cm)		NA	NA	0.94	0.90	0.77 to 1.07	0.234
c) Cross-section (every 1 cm)		NA	NA	0.74	0.71	0.59 to 0.85	<0.001
Kidney							
a) Cortical cyst		21	0.6	0.76	0.65	0.21 to 1.96	0.443
b) Stone		64	1.9	1.17	1.08	0.61 to 1.91	0.787
Other ultrasonography findings							
a) Gallstone		182	5.4	1.13	1.23	0.87 to 1.74	0.241
b) Sludge		95	2.8	112.1	97.7	30.8 to 310.5	<0.001
c) Intrahepatic duct stone		7	0.2	19.5	17.4	2.1 to 147.1	0.009

epg = eggs per gram; APF = advanced periductal fibrosis; OR = odds ratio; CI = confidence interval; NA = not applicable;

*Odds ratio adjusted for age, sex, and intensity of *Opisthorchis viverrini* infection statistical analysis
