# Supplementary material for: Periductal Fibrosis and Cholangiocarcinoma-Related Outcomes in Liver Fluke-Endemic Regions: A Systematic Review and Meta-Analysis
Source: Med Sci (Basel). 2026 Jul 9;14(3):380. doi: 10.3390/medsci14030380 (PMC13414246; doi:10.3390/medsci14030380)
Supplement: Supplementary file 1 [file medsci-14-00380-s001.zip › included papers/Moungthard et al., 2023.pdf]

RESEARCH ARTICLE

Editorial Process: Submission:06/28/2023 Acceptance:12/17/20

Association of Cholangiocarcinoma among People with Periductal Fibrosis

Hathaiwan Moungthard[1], Kavin Thinkhamrop[2,3,4,5]*, Nittaya Chamadol[2,3,6], Vallop Laopaiboon[2,3,4], Bandit Thinkhamrop[1,2,3,5], Matthew Kelly[7]

Abstract

Background: Cholangiocarcinoma (CCA) is a highly fatal tumor, and the most favorable chance for long-term survival lies in curative resection. Periductal fibrosis (PDF), a precancerous condition associated with chronic inflammation of the bile ducts, can serve as a screening marker for CCA using hepatobiliary ultrasonography (US). However, limited studies have explored the relationship between PDF and CCA. This study aimed to investigate the association between PDF and CCA in a population at risk in Northeast Thailand. **Methods:** The study included participants enrolled in the Cholangiocarcinoma Screening and Care Program (CASCAP) between 2013 and 2021 who underwent US. Histological evaluations were conducted following the standard protocol of the tertiary hospital at Khon Kaen University, Thailand. PDF was defined as the presence of fibrosis in the peripheral (PDF1), segmental (PDF2), or main bile duct (PDF3), diagnosed by well-trained general practitioners or radiologists. The association between PDF and CCA was assessed using multiple logistic regression, calculating adjusted odds ratios (AORs) and 95% confidence intervals (CIs). **Results:** Out of 751,061 participants, the overall prevalence of PDF was 115,267 (15.35%), with an overall CCA rate of 0.11%. The rates of CCA were 0.1%, 0.15%, and 0.27% in participants with PDF1, PDF2, and PDF3, respectively. After adjusting for gender, age at enrollment, education levels, history of O. viverrini infection, smoking, and alcohol consumption, the AORs for CCA were 0.94 (95% CI: 0.74 - 1.20), 1.4 (95% CI: 1.03 - 1.91), and 2.52 (95% CI: 1.38 - 4.58) for participants with PDF1, PDF2, and PDF3, respectively. **Conclusion:** Our findings demonstrate a significant association between fibrosis of the segmental and main bile ducts (PDF2 and PDF3) and CCA, with the strongest association observed in participants with PDF3. Hepatobiliary US screening could serve as a valuable tool for early detection of CCA, enabling timely curative treatment.

Keywords: periductal fibrosis- cholangiocarcinoma- ultrasonography- CASCAP- Thailand

Asian Pac J Cancer Prev, **24 (12)**, 4147-4154

Introduction

The global incidence of Cholangiocarcinoma (CCA) has increased in recent decades. The age-standardized incidence of CCA shows considerable geographical variation, with the highest incidence in Asia. Incidence is particularly high in the northeastern part of Thailand where the incidence rate was 85 per100,000, while in the incident rate in Canada was 0.4 per 100,000. The mortality rate of CCA shows variation by region as well. The highest annual mortality rate of CCA in the world was more than 6 per 100,000 in eastern regions such as Thailand, China, Taiwan and South Korea, while in Western region the rate was less than 4 per 100,000 (Banales et al., 2020). The high incidence levels in Thailand may be explained by highly prevalent Opisthorchis viverrini infection, which has been associated with increased risk of CCA (Sripa et al., 2011; Tyson and El-Serag, 2011; Thinkhamrop et al., 2021). Other factors such as hepatitis B, hepatitis C, liver disease, and bile duct diseases such as periductal fibrosis (PDF) also pose potential risk for CCA (Tyson and El-Serag, 2011; Khan et al., 2019; Clements et al., 2020; Thinkhamrop et al., 2022)(Chamadol et al., 2014).

The pathogenesis of CCA involves chronic inflammation that affects various morphological transformations from small to large bile duct sites. Chronic inflammation can

*[1]Epidemiology and Biostatistics Program, Faculty of Public Health, Khon Kaen University, Khon Kaen, 40002, Thailand. [2]Cholangiocarcinoma Screening and Care Program (CASCAP), Faculty of Medicine, Khon Kaen University, Khon Kaen, 40002, Thailand. [3]Cholangiocarcinoma Research Institute (CARI), Khon Kaen, 40002, Thailand. [4]Health and Epidemiology Geoinformatics Research (HEGER), Faculty of Public Health, Khon Kaen University, Khon Kaen, 40002, Thailand. [5]Department of Radiology, Faculty of Medicine, Khon Kaen University, Khon Kaen, 40002, Thailand. [6]Data Management and Statistical Analysis Center (DAMASAC), Faculty of Public Health, Khon Kaen University, Khon Kaen, 40002, Thailand. [7]Department of Global Health, Research School of Population Health, Australian National University, Canberra, ACT, 2601, Australia. *For Correspondence: kavith@kku.ac.th*

be detected using radiological imaging techniques such as ultrasound, computed tomography (CT), and magnetic resonance imaging (MRI). Ultrasound (US) is a widely used screening tool for liver and biliary disease due to its accessibility and cost-effectiveness (Thinkhamrop et al., 2022). US is particularly useful in investigating bile duct abnormalities and detecting early cancer lesions (Mairiang et al., 2012). PDF, identified by US, serves as a precursor lesion that requires further investigation with CT or MRI for precise lesion localization. PDF can be identified using US and does not necessarily require the expertise of a radiologist, thereby increasing access to screening. Moreover, PDF can be detected earlier than bile duct dilatation (BDD), another CCA precursor, allowing for more effective intervention (Chamadol et al., 2017; Chamadol et al., 2019). Given that the majority of CCA patients present in late stages, especially in the endemic area of northeastern Thailand (Khuntikeo et al., 2020), the use of US screening to identify PDF may prove to be an effective tool for detecting early-stage CCA. This approach aims to increase the number of patients diagnosed at an early stage and reduce CCA-related mortality, as PDF represent a pre-pathologic disease that may predict CCA (Chamadol et al., 2014). This study aims to investigate the association between PDF and CCA among the population at risk in northeastern Thailand.

Materials and Methods

Design overview

This cross-sectional study involved the inclusion of all participants who underwent US examination and received a pathological diagnosis of CCA from the Cholangiocarcinoma Screening and Care Program (CASCAP), which is a notable CCA screening project conducted in northeastern Thailand (Khuntikeo et al., 2015). The program specifically targeted a high-risk population, and recruited patients at a range of tertiary hospitals across Northeast Thailand, as well as mobile screening clinics at District Health centers.

The data used for this study spanned the period between 2013 and 2021. Histological findings were determined in accordance with the standard protocol employed at the tertiary hospital located at Khon Kaen University, Thailand. The classification of the participants was based on the anatomical location of the intrahepatic and extrahepatic bile duct. Skilled general practitioners or radiologists, who were part of the CASCAP team, conducted the diagnosis. They classified the participants into three categories, namely PDF1, PDF2, and PDF3. PDF1 was defined as the presence of high echogenicity in the wall of small bile ducts scattered throughout the liver, exhibiting a starry sky pattern. PDF2 referred to high echogenicity observed along the segmental bile duct wall, running parallel to the portal vein. Finally, PDF3 indicated high echogenicity along the main bile duct wall, also running parallel to the portal vein, but within the periportal space. The demographic data collected included gender, age at enrollment, education levels, main occupation, history of *O. viverrini* infection, history of praziquantel (PZQ) treatment, history of smoking, and alcohol consumption.

Primary outcomes and study factors

The primary outcome of this study was the categorization of CCA status based on histological findings obtained through biopsies. CCA status was categorized into two groups: “no” and “yes”. The data pertaining to the histological findings were collected following the standard protocol implemented at the tertiary hospital located at Khon Kaen University, Thailand.

The study also investigated several independent variables, with particular focus on the PDFs. The PDFs were categorized as no-PDF, PDF1, PDF2, and PDF3, and this variable served as the factor of interest in the analysis. Additionally, other factors such as gender (female/male), age at enrollment in years, education levels (none to secondary, certificate and higher), main occupation (farmer, non-farmer), history of *O. viverrini* infection, history PZQ treatment, history of smoking, and alcohol consumption were collected using an enrolment questionnaire.

Statistical analysis

The baseline characteristics of the sample population and PDF diagnosis were summarized using frequency numbers and percentages for categorical data. These included variables such as gender, age groups, occupations, education levels, history of *O. viverrini* infection, history of PZQ treatment, history of smoking, and history of alcohol consumption. For continuous data, such as age at enrollment in year, the summary statistics provided were the mean with standard deviation (SD), median, minimum and maximum range.

The rate of CCA was estimated both overall and separately for each category of the aforementioned factors. To investigate the association between PDF and CCA, potential candidate variables were explored for inclusion in a multivariable model. This step aimed to control for the effects of these variables on the relationship between PDF and CCA. Crude odds ratios (OR) and their corresponding 95% confidence intervals (CI) were calculated to measure the association between each factor and CCA. Simple logistic regression was employed, examining one factor at a time. Factors with a p-value less than 0.25 or factors reported to be associated with CCA in previous studies were included in the multivariable model. Multiple logistic regression was then used to estimate the adjusted OR and their 95% CI, quantifying the relationship between PDF and CCA while accounting for all other included factors.

For the analysis, STATA version 15 (Stata Corp, College Station, TX, United States) was utilized. The statistical significance level was set at a p-value of less than 0.05.

Results

Characteristics of study participants

Out of a total of 751,061 participants, the mean age was 54.85 years (SD = 9.47). Approximately two-thirds of the participants were female (63.21%). Among these participants, the overall rate of PDF diagnosis was

Table 1. Demographic Characteristics of Study Participants

Characteristics	Overall		PDF1		PDF2		PDF3	
	Number	%	Number	%	Number	%	Number	%
Gender								
Female	474,748	63.21	47,812	58.88	17,455	58.09	2,202	54.97
Male	276,258	36.79	33,396	41.12	12,591	41.91	1,804	45.03
Age group (years)								
<50	233,535	31.17	27,246	33.64	9,732	32.49	1,068	26.69
50-59	310,479	41.45	31,475	38.87	11,247	37.54	1,558	38.94
≥60	205,096	27.38	22,263	27.49	8,978	29.97	1,375	34.37
Mean (Standard deviation)	54.85 (9.47)		54.65 (9.76)		55.26 (10.12)		56.52 (10.13)	
Educational level								
None to secondary	571,427	86.27	62,655	87.24	23,119	87.67	3,116	87.95
Certificate and higher	90,924	13.73	9,164	12.76	3,250	12.33	427	12.05
Occupation								
Non farmer	164,546	21.94	14,619	18.03	5,519	18.41	853	21.32
Farmer	585,585	78.06	66,451	81.97	24,456	81.59	3,147	78.67
History of O. viverini infection								
No	651,550	86.75	68,708	84.6	24,703	82.21	3,424	85.47
Yes	99,511	13.25	12,505	15.4	5,345	17.79	582	14.53
History of Praziquantel treatment								
None	574,855	76.54	60,103	74.01	22,039	73.35	3,045	76.01
Yes	176,206	23.46	21,110	25.99	8,009	26.65	961	23.99
Smoking history								
No	604,216	80.64	62,442	77.18	23,164	77.51	3,072	76.84
Yes	145,062	19.36	18,461	22.82	6,722	22.49	926	23.16
Alcohol consumption history								
No	429,687	57.3	44,173	54.52	16,693	55.74	2,230	55.75
Yes, current or previous	320,186	42.7	36,845	45.48	13,255	44.26	1,770	44.25

15.35%. Among those diagnosed with PDF, the majority were diagnosed with PDF1 (10.81%), followed by PDF2 (4%), and PDF3 (0.53%). A significant proportion of the participants (68.83%) were aged 50 years and above. The occupation with the highest representation among the participants was farming, accounting for 78.06% of the sample. About 13.25% of the participants had a history of *O. viverrini* infection, while 23.46% had received

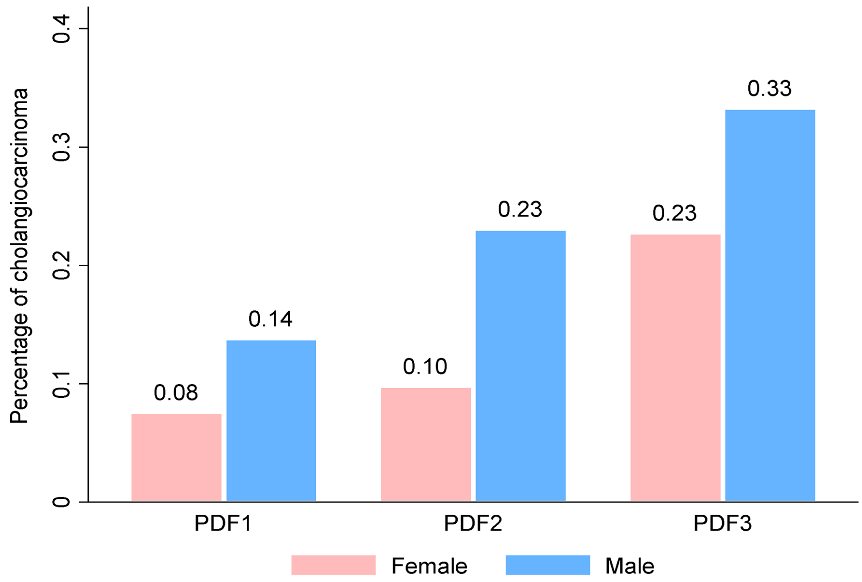

Figure 1. Percentage of Cholangiocarcinoma by Sex According to Periductal Fibrosis (PDF) Status.

Table 2. Association between PDF and CCA for each Factor Using Simple Logistic Regression

Factors	Number of participants	CCA positive		Crude OR	95% CI
		n	%		
Overall	751,061	798	0.11	NA	NA
Periductal fibrosis (PDF)					
Non	635,794	659	0.1	1	
PDF1	81,213	82	0.1	0.97	0.77 - 1.23
PDF2	30,048	46	0.15	1.48	1.10 - 1.99
PDF3	4,006	11	0.27	2.65	1.46 - 4.82
Gender					
Female	474,748	336	0.07	1	
Male	276,258	462	0.17	2.37	2.05 - 2.72
Age group (years)					
<50	233,535	96	0.04	1	
50-59	310,479	313	0.1	2.45	1.95 - 3.08
≥60	205,096	386	0.19	4.59	3.67 - 5.73
Educational level					
None to secondary	571,427	655	0.11	1	
Certificate and higher	90,924	70	0.08	0.67	0.52 - 0.86
Occupation					
Non farmer	164,546	167	0.1	1	
Farmer	585,585	631	0.11	1.06	0.89 - 1.26
History of O. viverini infection					
No	651,550	597	0.09	1	
Yes	99,511	201	0.2	2.21	1.88 - 2.59
History of Praziquantel treatment					
None	574,855	528	0.09	1	
Yes	176,206	270	0.15	1.67	1.44 - 1.93
Smoking history					
No	604,216	474	0.08	1	
Yes	145,062	324	0.22	2.85	2.48 - 3.28
Alcohol consumption history					
No	429,687	336	0.08	1	
Yes, current or previous	320,186	462	0.14	1.85	1.60 - 2.13

CCA, Cholangiocarcinoma; n, Number of CCA cases; NA, Not applicable; OR, odds ratio from simple logistic regression; 95% CI, 95% confidence interval of crude OR

treatment for liver fluke infection with PZQ (Table 1).

Association between PDFs and CCA
Bivariate analysis

Among 751,061 participants who underwent hepatobiliary US and received a pathological diagnosis, the overall rate of CCA was found to be 0.11% (798/751,061). When considering the classification of PDF, participants diagnosed with PDF1 had a CCA rate of 0.1%, while those with PDF2 had a rate of 0.15%, and PDF3 had the highest rate at 0.27% (Table 2). Further examining the association by gender, it was observed that male participants exhibited higher PDF abnormalities. Specifically, the CCA rates for males with PDF1, PDF2, and PDF3 were 0.14%, 0.23%, and 0.33% respectively (Figure 1).

Performing bivariate analysis using simple logistic regression, it was found that compared to participants without PDF, those diagnosed with PDF2 had an increased crude OR of 1.48 (95% CI: 1.10 - 1.99), and PDF3 had and even higher OR of 2.65 (95% CI: 1.46 - 4.82). These results were statistically significant, with a p-value of 0.004. Additionally, several other factors were found to be significantly associated with CCA. These factors included male gender, age at enrollment of 50 years and over, education level of certificate or higher, *O. viverrini* infection, use of PZQ treatment, smoking, and alcohol consumption (Table 2 and Figure 1).

Multivariable analysis

Results from multivariable analysis using multiple logistic regression revealed statistically significant adjusted OR for associations between PDFs and CCA. Controlling for the effects of gender, age at enrollment, education levels, history of *O. viverrini* infection,

Table 3. Association between PDF and CCA for Each Factor Controlling for All Others Effect Using Multiple Logistic Regression.

Factors	Number of participants	CCA positive n	CCA positive %	Crude OR	Adjusted OR	95% CI
Periductal fibrosis (PDF)						
Non	635,794	659	0.1	1	1	
PDF1	81,213	82	0.1	0.97	0.94	0.74 - 1.20
PDF2	30,048	46	0.15	1.48	1.4	1.03 - 1.91
PDF3	4,006	11	0.27	2.65	2.52	1.38 - 4.58
Gender						
Female	474,748	336	0.07	1	1	
Male	276,258	462	0.17	2.37	1.34	1.11 - 1.62
Age group (years)						
<50	233,535	96	0.04	1	1	
50-59	310,479	313	0.1	2.45	2.18	1.70 - 2.79
≥60	205,096	386	0.19	4.59	3.84	3.00 - 4.92
Educational level						
None to secondary	571,427	655	0.11	1	1	
Certificate and higher	90,924	70	0.08	0.67	0.9	0.69 - 1.16
History of O. viverini infection						
No	651,550	597	0.09	1	1	
Yes	99,511	201	0.2	2.21	1.87	1.58 - 2.21
Smoking history						
No	604,216	474	0.08	1	1	
Yes	145,062	324	0.22	2.85	1.79	1.46 - 2.18
Alcohol consumption history						
No	429,687	336	0.08	1	1	
Yes, current or previous	320,186	462	0.14	1.85	1.3	1.08 - 1.55

CCA, Cholangiocarcinoma; n, Number of CCA cases; NA, Not applicable; OR, odds ratio from multiple logistic regression; 95% CI, 95% confidence interval of adjusted OR

smoking, and alcohol consumption, the adjusted ORs for PDF1, PDF2, and PDF3 were calculated.

After adjusting for the aforementioned factors, the adjusted OR for PDF1 was found to be 0.94 (95% CI: 0.74 - 1.20), indicating no statistically significant association with CCA. However, for PDF2, the adjusted

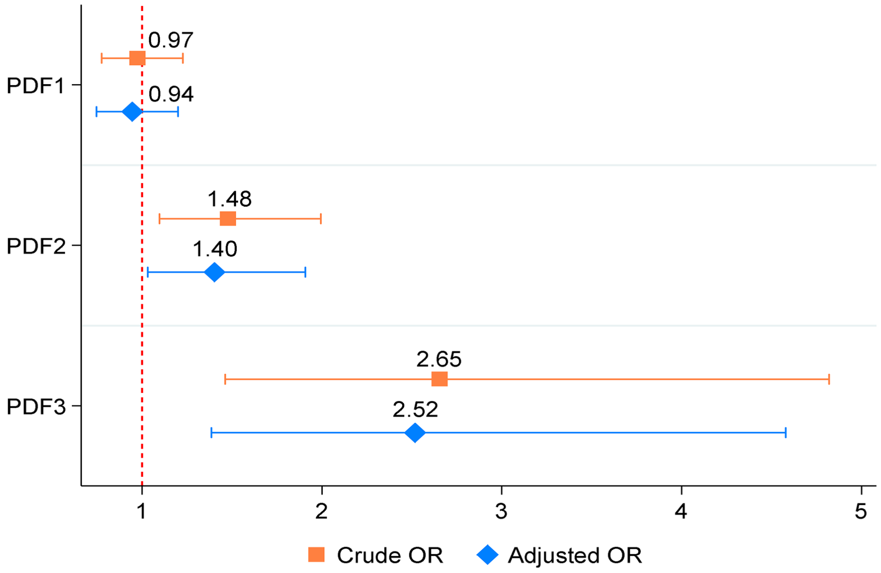

Figure 2. Crude and Adjusted Odds Ratio (OR) of Association between Periductal Fibrosis (PDF) and Cholangiocarcinoma.

OR was 1.40 (95% CI: 1.03 - 1.91), indicating a significant positive association with CCA. Similarly, PDF3 had an adjusted OR of 2.52 (95% CI: 1.38 - 4.58), indicating a significant positive association with CCA. These results were obtained in comparison to the reference category of non-PDF (Table 3). The p-value for this analysis was 0.003. Furthermore, several other factors were found to be significantly associated with CCA, even after adjusting for potential confounders. These factors included male gender, age at enrollment of 50 years and over, history of *O. viverrini* infection, smoking, and alcohol consumption. For a visual comparison, Figure 2 displays the crude OR obtained from the bivariate analyses alongside the adjusted ORs calculated from the multivariable model.

Discussion

Our study aimed to investigate the association between PDF (biliary abnormalities) and CCA. The overall rate of CCA in our study was 0.11% (798/751,061). Although this rate may not appear exceptionally high when compared to previous studies reporting high incident rates of CCA in the northeastern part of Thailand (Green et al., 1991; Khan et al., 2019), it is still significant compared to other countries. Unfortunately, CCA often presents at a late stage, leading to poor survival outcomes despite the declining incidence in recent years (Banales et al., 2016; Luvira et al., 2016; Khan et al., 2019). To improve survival rates, the use of US as a safe and accessible tool for detecting abnormalities, including precancerous lesions, is crucial (Chamadol et al., 2019; Khuntikeo et al., 2020). Our study demonstrates the significant role of US-detected PDF in association with CCA, making it the first report highlighting the importance of PDF in CCA detection. This PDF identification can play a key role in identifying early stage CCA. In a recent publication, Khuntikeo et al. demonstrated that PDF findings could detect early-stage CCA in a screening group (Khuntikeo et al., 2020), but the association between PDF findings and CCA remained unclear.

Our study provides evidence that PDF abnormalities indicate the presence of bile duct abnormalities, ranging from small bile duct to the main bile duct, which may potentially develop into bile duct cancer in the future. The prevalence of CCA increased progressively from 0.1% in PDF1, 0.15% in PDF2, and 0.27% in PDF3. In the multivariable model, after controlling for factors such as gender, age at enrollment, education levels, history of *O. viverrini* infection, smoking, and alcohol consumption, our study found that both PDF2 and PDF3 categories were significantly associated with a higher likelihood of CCA compared to the non-PDF group. These findings align with a previous study conducted in 2019, which demonstrated a highly significant association between PDF3 and bile duct dilatation (BDD), a key indicator for CCA development (adjusted OR = 5.74; 95% CI 4.57 - 7.21) (Chamadol et al., 2019). On the other hand, the PDF1 group was found to be less likely to have CCA (6%) compared to the non-PDF group, although this result did not reach statistically significance.

Interestingly, our study observed a higher proportion of female participants than male participants. However, CCA was more common in males across all PDF abnormality groups: PDF1 (0.14% vs. 0.08%), PDF2 (0.23% vs. 0.10%), and PDF3 (0.33% vs. 0.23%). This discrepancy may be attributed to male consumption habits, such as a preference for alcohol and raw fish, which are major risk factors for biliary tract disorders, including PDF (Chamadol et al., 2019; Thinkhamrop et al., 2020), as well as potential genetic and molecular differences (Kim et al., 2018). Additionally, the majority of participants in all PDF groups in our study were aged 60 years or older, which is consistent with the findings of Chamadol et al. in 2019, suggesting that age may influence bile duct diameter (Chamadol et al., 2019).

In addition to PDF; our study identified several other factors that were significantly associated with CCA, consistent with previous studies. These factors included male gender compared to female, age at enrollment of 50 years and older compared to younger age, history of *O. viverrini* infection, smoking, and alcohol consumption (Sripa et al., 2011; Tyson and El-Serag, 2011; Suwannatrai et al., 2019; Thinkhamrop et al., 2020; Thinkhamrop et al., 2021). However, in terms of education levels, our study found a statistically significant association in the crude analysis between higher education and CCA (without considering other factors), but this association did not remain significant in the multivariable analysis when adjusted for all other factors. This discrepancy suggests that other factors may influence the association between education levels and CCA. Nevertheless, we retained this factor in the multivariable model because previous evidence has shown an association between education levels and CCA (Sripa et al., 2011; Tyson and El-Serag, 2011; Thinkhamrop et al., 2021), indicating that education level acts as a confounding factor that needs to be controlled for.

Our study possessed several strengths that enhance its validity and significance. Firstly, we had a substantial sample size, comprising a large number of participants from a region with a high incidence of CCA. This characteristic enhances the generalizability of our findings and allows for potential representation of populations worldwide at high risk of CCA. Secondly, our study made a notable contribution by being the first to report an association between PDF and CCA. This novel finding highlights the potential of PDF as a valuable screening tool for the early detection of CCA. By identifying this link, our study provides important insights that could aid in improving diagnostic and screening strategies for CCA.

Our study has several limitations that should be considered when interpreting the results. Firstly, the sample size may not be representative of the entire Thai population as we focused on participants from the northeastern part of Thailand. Therefore, caution should be exercised when generalizing the findings to other regions or populations. Secondly, some of the study factors were collected through self- interviews, which could introduce recall bias. Variables such as the history of *O. viverrini* infection, PZQ treatments, smoking, and alcohol consumption may be subject to inaccuracies or incomplete recollection by participants. Another limitation

is the lack of validation regarding the liver performance status of the patients, which could potentially influence the interpretation of pathological changes and their association with the disease. Additionally, it is important to acknowledge that US as a diagnostic tool is operator dependent, meaning that different operators may have varying levels of expertise and interpretations. This variability could introduce inconsistencies or differences in the assessment of PDF and its association with CCA. Considering these limitations, further studies with larger and more diverse populations, as well as validation of clinical parameters, are warranted to strengthen the findings and enhance the understanding of the association between PDF and CCA.

In conclusion, our study has highlighted an important finding regarding the association between segmental and main ductal fibrosis (PDF2 and PDF3) and CCA. We observed a significant relationship, with the highest association found in participants with PDF3 compared to those without PDF. These results emphasize the potential of hepatobiliary US screening as a valuable tool for the early detection of abnormalities, prompting further evaluation through imaging techniques such as CT or MRI. Early detection is crucial for facilitating curative care in the early stages of cancer. Further research is needed to validate these findings and explore the feasibility and effectiveness of incorporating hepatobiliary US screening into routine clinical practice for the early detection of CCA.

Author Contribution Statement

HM, KT, and BT initiated the idea, and provided constructive criticism and edited of the drafts of the manuscripts. NC and VL performed the ultrasonography and edit the drafts of the manuscripts. HM, KT, and BT performed data management and data quality assurance, data analysis, and wrote all statistical methods and the results sections of the manuscript. HM, KT, NC, VL, BT, and MK initiated the idea, provided feedback and edited the drafts of the manuscript. All authors have seen and approved the final version of the manuscript. .

Acknowledgements

The authors are truly thankful for all members of CASCAP, particularly the cohort members and staff from all participating institutions including the Ministry of Public Health, Ministry of Interior, and Ministry of Education of Thailand. This research was supported by NSRF under the Basic Research Fund of Khon Kaen University through Cholangiocarcinoma Research Institute.

Approval

This paper is a part of the dissertation submitted in fulfillment of the requirements for the degree of Epidemiology and Biostatistics Program, Faculty of Public Health, Khon Kaen University, Thailand.

Ethics considerations

The Khon Kaen University Ethics Committee for Human Research approved the research protocol, reference number HE651112 which requested the data from Cholangiocarcinoma Screening and Care Program (CASCAP). The CASCAP data collection was conducted according to the principles of Good Clinical Practice, the Declaration of Helsinki, and national laws and regulations about clinical studies. It was approved by the Khon Kaen University Ethics Committee for Human Research under the reference number HE551404. All subjects gave written, informed consent to participate in the study and for their anonymized data to be used for statistical analysis and dissemination.

Availability of data and materials

The datasets used and/or analyzed during the current study are available from the corresponding author on reasonable request.

Competing interest

The authors declare that they have no competing interests.

References

Banales JM, Cardinale V, Carpino G, et al (2016). Expert consensus document: Cholangiocarcinoma: current knowledge and future perspectives consensus statement from the European Network for the Study of Cholangiocarcinoma (ENS-CCA). *Nat Rev Gastroenterol Hepatol*, **13**, 261-80.

Banales JM, Marin JJG, Lamarca A, et al (2020). Cholangiocarcinoma 2020: the next horizon in mechanisms and management. *Nat Rev Gastroenterol Hepatol*, **17**, 557-88.

Chamadol N, Khuntikeo N, Thinkhamrop B, et al (2019). Association between periductal fibrosis and bile duct dilatation among a population at high risk of cholangiocarcinoma: a cross-sectional study of cholangiocarcinoma screening in Northeast Thailand. *BMJ Open*, **9**, e023217.

Chamadol N, Laopaiboon V, Srinakarin J, et al (2017). Teleconsultation ultrasonography: a new weapon to combat cholangiocarcinoma. *ESMO Open*, **2**, e000231.

Chamadol N, Pairojkul C, Khuntikeo N, et al (2014). Histological confirmation of periductal fibrosis from ultrasound diagnosis in cholangiocarcinoma patients. *J Hepatobiliary Pancreat Sci*, **21**, 316-22.

Clements O, Eliahoo J, Kim JU, et al (2020). Risk factors for intrahepatic and extrahepatic cholangiocarcinoma: A systematic review and meta-analysis. *J Hepatol*, **72**, 95-103.

Green A, Uttaravichien T, Bhudhisawasdi V, et al (1991). Cholangiocarcinoma in north east Thailand. A hospital-based study. *Trop Geogr Med*, **43**, 193-8.

Khan SA, Tavolari S, Brandi G (2019). Cholangiocarcinoma: Epidemiology and risk factors. *Liver Int*, **39**, 19-31.

Khuntikeo N, Chamadol N, Yongvanit P, et al (2015). Cohort profile: cholangiocarcinoma screening and care program (CASCAP). *BMC Cancer*, **15**, 459.

Khuntikeo N, Koonmee S, Sa-Ngiamwibool P, et al (2020). A comparison of the proportion of early stage cholangiocarcinoma found in an ultrasound-screening program compared to walk-in patients. *HPB (Oxford)*, **22**, 874-83.

Kim HI, Lim H, Moon A (2018). Sex Differences in Cancer:

Epidemiology, Genetics and Therapy. *Biomol Ther (Seoul)*, **26**, 335-42.

Luvira V, Nilprapha K, Bhudhisawasdi V, et al (2016). Cholangiocarcinoma Patient Outcome in Northeastern Thailand: Single-Center Prospective Study. *Asian Pac J Cancer Prev*, **17**, 401-6.

Mairiang E, Laha T, Bethony JM, et al (2012). Ultrasonography assessment of hepatobiliary abnormalities in 3359 subjects with Opisthorchis viverrini infection in endemic areas of Thailand. *Parasitol Int*, **61**, 208-11.

Sripa B, Bethony JM, Sithithaworn P, et al (2011). Opisthorchiasis and Opisthorchis-associated cholangiocarcinoma in Thailand and Laos. *Acta Trop*, **120**, 158-68.

Suwannatrai AT, Thinkhamrop K, Clements ACA, et al (2019). Bayesian spatial analysis of cholangiocarcinoma in Northeast Thailand. *Sci Rep*, **9**, 14263.

Thinkhamrop K, Khuntikeo N, Chamadol N, et al (2022). Associations between ultrasound screening findings and cholangiocarcinoma diagnosis in an at-risk population. *Sci Rep*, **12**, 13513.

Thinkhamrop K, Khuntikeo N, Laohasiriwong W, et al (2021). Association of comorbidity between Opisthorchis viverrini infection and diabetes mellitus in the development of cholangiocarcinoma among a high-risk population, northeastern Thailand. *PLoS Negl Trop Dis*, **15**, e0009741.

Thinkhamrop K, Suwannatrai AT, Chamadol N, et al (2020). Spatial analysis of hepatobiliary abnormalities in a population at high-risk of cholangiocarcinoma in Thailand. *Sci Rep*, **10**, 16855.

Tyson GL, El-Serag HB (2011). Risk factors for cholangiocarcinoma. *Hepatology*, **54**, 173-84.

This work is licensed under a Creative Commons Attribution-Non Commercial 4.0 International License.
