# Supplementary material for: Periductal Fibrosis and Cholangiocarcinoma-Related Outcomes in Liver Fluke-Endemic Regions: A Systematic Review and Meta-Analysis
Source: Med Sci (Basel). 2026 Jul 9;14(3):380. doi: 10.3390/medsci14030380 (PMC13414246; doi:10.3390/medsci14030380)
Supplement: Supplementary file 1 [file medsci-14-00380-s001.zip › included papers/Soukhathammavong et al., 2015.pdf]

Acta Tropica 141 (2015) 303–309

Contents lists available at ScienceDirect

Acta Tropica

journal homepage: www.elsevier.com/locate/actatropica

Subtle to severe hepatobiliary morbidity in *Opisthorchis viverrini* endemic settings in Southern Laos

Phonepasong Ayé Soukhathammavong[a,b,c], Virasack Rajpho[d], Khampheng Phongluxa[a,b,c], Youthanavanh Vonghachack[b,c,d], Jan Hattendorf[b,c], Bouasy Hongvanthong[e], Oroth Rasaphon[f], Banchob Sripa[g,h], Kongsap Akkhavong[a], Christoph Hatz[b,c,i], Peter Odermatt[b,c,*]

[a] *National Institute of Public Health, Ministry of Health, Vientiane Capital, Lao People's Democratic Republic*
[b] *Swiss Tropical and Public Health Institute, P.O. Box, CH-4002 Basel, Switzerland*
[c] *University of Basel, P.O. Box, CH-4003 Basel, Switzerland*
[d] *Faculty of Basic Science, University of Health Science, Vientiane Capital, Lao People's Democratic Republic*
[e] *National Center of Malariology, Parasitology and Entomology (CMPE), Ministry of Health, Vientiane, Lao People's Democratic Republic*
[f] *Mahosot Hospital, Ministry of Health, Vientiane, Lao People's Democratic Republic*
[g] *Department of Pathology, Faculty of Medicine, Khon Kaen University, Khon Kean, Thailand*
[h] *Liver Fluke and Cholangiocarcinoma Research Centre, Faculty of Medicine, Khon Kaen University, Khon Kean, Thailand*
[i] *Institute of Social and Preventive Medicine, University of Zürich, Zürich, Switzerland*

ARTICLE INFO

Article history:
Available online 29 September 2014

Keywords:
Opisthorchis viverrini
Hepatobiliary morbidity
Cholangiocarcinoma
Ultrasonography
Laos

ABSTRACT

Evidence of severe hepatobiliary morbidity associated with *Opisthorchis viverrini* liver fluke infection including cholangiocarcinoma (CCA) is scarce in Laos although *O. viverrini* infection is highly prevalent. We assessed hepatobiliary morbidity using abdominal ultrasonography (US) in *O. viverrini* adult patients in Saravan province, Southern Laos. A random sample of 431 *O. viverrini* patients from 10 villages underwent abdominal US. Mild, moderate and markedly advanced periductal fibrosis was diagnosed in 7.0%, 66.5%, and 17.0% of patients, respectively. Normal liver parenchyma was seen in only 9.5% of patients. Presence of gall stones (13.2%), sludge (1.4%), gall wall thickening (1.2%), bile duct dilatation (1.6%), fatty liver (12.0%), kidney stones (8.6%) and cysts (7.9%) were diagnosed in considerable frequencies. In five patients (1.2%) hepatobiliary lesions suggesting CCA were diagnosed. Tumour markers, i.e. Interleukin-6, plasminogen activator inhibitor and carbohydrate antigen 19-9 were within normal range. The number of CCA suspected liver masses and hepatobiliary morbidity diagnosed among clinically asymptomatic adult patients in *O. viverrini* endemic area presents a major public health concern in Laos. However, definitive diagnosis of *Opisthorchis*-related severe sequelae including CCA is urgently needed to gauge the burden of this deadly disease in Laos.

© 2014 Elsevier B.V. All rights reserved.

1. Introduction

In Lao People's Democratic Republic (Lao RDP, Laos), information on morbidity due to *Opisthorchis viverrini* infection is scarce and absent for cholangiocarcinoma (CCA), a bile duct cancer associated with chronic *O. viverrini* infection. A recent study documented morbidity associated with liver flukes *O. viverrini* and *Schistosoma mekongi* infections in southern Laos (Sayasone et al., 2012). However, CCA cases and precursor lesions were not assessed. Infection prevalences of *O. viverrini* in southern Laos (Forrer et al., 2012; Phongluxa et al., 2013; Rim et al., 2003; Sayasone et al., 2007, 2011) suggest that hepatobiliary morbidity and CCA incidence in Laos are at least as high as or higher than in northeast Thailand, where similar *O. viverrini* infection prevalences are present.

CCA is a rare bile-duct cancer with a poor prognosis. Chronic *O. viverrini* liver fluke infection is a major risk factor for CCA (Sripa et al., 2012; Sithithaworn et al., 2014). *O. viverrini* has been classified as a carcinogenic agent (Bouvard et al., 2009). The highest CCA incidence worldwide is recorded in *O. viverrini* endemic areas in northeast Thailand (Sripa et al., 2011) where on average 119 CCA cases per 100,000 persons occur each year among adults aged

* Corresponding author at: Department of Epidemiology and Public Health, Swiss Tropical and Public Health Institute, P.O. Box, CH-4002 Basel, Switzerland. Tel.: +41 61 284 8214; fax: +41 61 284 8105.
E-mail address: peter.odermatt@unibas.ch (P. Odermatt).

http://dx.doi.org/10.1016/j.actatropica.2014.09.014
0001-706X/© 2014 Elsevier B.V. All rights reserved.

35–64 (Blechacz and Gores, 2008) and approximately 5000 cases are diagnosed annually (Sripa et al., 2007). Medical and wage costs associated with CCA and *O. viverrini* fluke infection amount to an estimated USD 120 million in Thailand alone (Sripa, 2008).

Opisthorchiasis is a fish-borne trematode infection belonging to the group of tropical infections known as neglected tropical diseases (NTDs) (Keiser and Utzinger, 2009). Almost 67 million people are at risk of infection. An estimated 10 million infected persons live in Northeast Thailand and Laos. One third of the 5.5 million inhabitants of Laos are infected (Sithithaworn et al., 2012). In Laos, *O. viverrini* infections occur in all provinces but the highest prevalences are seen in the Central and Southern provinces, with rates reaching up to 90% of the population (Forrer et al., 2012; Sayasone et al., 2009, 2011). Although infection rates are very high, few studies have examined morbidity associated with the infection. Public health control activities are largely lacking.

We assessed hepatobiliary morbidity including lesions suggestive of CCA in infected adults in rural *O. viverrini* endemic communities of Southern Laos.

2. Population, materials and methods

2.1. Ethical considerations

Ethical approval was granted by the Lao National Ethics Committee for Health Research (NECHR, no. 278/NECHR) and by the Ethical Review Group of the World Health Organization-Western Pacific Region in Manila, Philippines to investigate hepatobiliary morbidity.

The study's objectives, procedures and potential risks and benefits were explained to village authorities and to all participating villagers in Lao and ethnic group languages. Informed written consent was obtained prior to enrollment. Patients were informed of any diagnosed infection and of ultrasonography (US) results and referred for treatment according to standard health care procedures of the Lao Ministry of Health (MOH, 2004). All patients with lesions suggestive of CCA were given additional counselling. A free follow-up investigation was proposed. All persons found to be stool positive for *O. viverrini* were treated with praziquantel (40 mg/kg, single oral dose).

2.2. Study area and population

From January to April 2011, a cross-sectional study was carried out in adults (aged $\geq$ 20 years) in 10 *O. viverrini* endemic villages in Saravan district, Saravan province, southern Laos. Approximately 350,000 inhabitants live in the province (9 districts, 168 villages). Parasitological studies in the Saravan district documented a high *O. viverrini* infection prevalence (>50%) in the general population (Sayasone et al., 2007). In addition, in-depth parasitological investigations, including demonstration of adult food-borne trematodes in human stool showed that almost all *O. viverrini* infected individuals were co-infected with minute intestinal flukes (MIF), in particular *Haplorchis taichui* (Sayasone et al., 2009).

For this study, 840 participants from randomly selected households were screened for *O. viverrini* infection (Fig. 1). Of these 85.0% had an *O. viverrini* infection. Abdominal US examination was performed in adults aged 20 years and older (431, 51.3%).

2.3. Laboratory analysis of stool and blood

Stool examinations followed a standard procedure. In brief, each participant provided two fresh stool samples on consecutive days. Stool containers were transferred to the laboratory the same morning. From each sample, two Kato-Katz thick smears were prepared using standard 41.7 mg templates and examined under a light

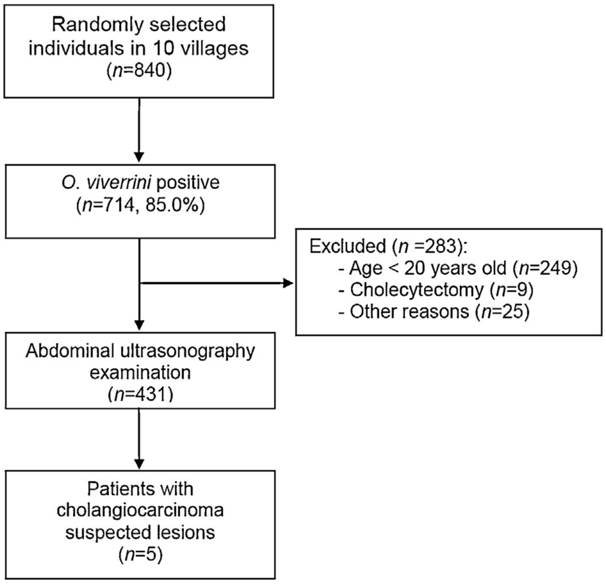

Fig. 1. Study flowchart: study participants in 10 villages, Saravan district, Saravan province, Laos.

microscope (100× magnification) (Katz et al., 1972). The number of *O. viverrini* eggs per slide was recorded. Slides were read within 30–45 min after preparation. Ten per cent of all smears were re-examined for quality control (Soukhathammavong et al., 2011). An *O. viverrini*-positive patient was defined as one with at least one *O. viverrini* egg present in at least one of the four Kato-Katz faecal thick smears.

From patients with CCA suspected lesions, a 30 ml venous blood sample was drawn. Tumour markers, i.e. carbohydrate antigen 19-9 (CA 19-9), Interleukin-6 (IL-6) and plasminogen activator inhibitor (PAI) were assessed in Khon Kaen University's reference laboratory (Sripa et al., 2009).

For each patient, demographic data were recorded (i.e. sex, age, place of residence, contact details and occupation, number of years of school attendance) and information on abdominal symptoms, raw fish consumption and the presence and utilization of latrine was obtained.

2.4. Assessment of hepatobiliary morbidity

Abdominal US examinations were performed in the study village using a portable US machine (SSD-500, Aloka, Tokyo, Japan) with a 3.5 MHz convex abdominal transducer. Patients were asked to fast 8 h before US examination. Liver parenchyma fibrosis was assessed using an adapted examination protocol from Niamey (Niamey Working Group, 2000) in combination with the standard protocols used earlier in community-based studies in Khon Kaen, Thailand (Mairiang et al., 2012). Liver parenchyma patterns were graded as normal or no echoes (=0), starry sky (=1+), rings and pipe stems (=2+), or highly echogenic 'patches' extending to peripheral areas (=3+). Patients were grouped into those with "none or mild advanced periportal fibrosis" and "advanced fibrosis" according to the US grade (equal $\leq$1 *versus* $\geq$2). Gallbladder was examined before and 30 min after consumption of a fatty meal (a sterilised milk of 250 mL and two boiled eggs).

US examiners (PAS, VR) were blinded to the laboratory results. Images were recorded on a DVD (Sony DVD recorder RDR-HX780) by senior radiologists (Oroth Rasphon, Mahosot Hospital; Dr. Eimorn Mairian, Khon Kaen University, Khon Kaen) for quality control.

Table 1
General characteristics of study participants ($n = 431$).

Characteristics	n (%)
Age (years)	
20–29	75 (17.4)
30–39	130 (30.2)
40–49	92 (21.3)
≥50+	134 (31.1)
Sex	
Male	182 (42.2)
Female	249 (57.8)
Ethnic group	
Lao Loum	294 (68.2)
Lao Theung	137 (31.8)
Profession	
Farmer	427 (99.5)
Other	4 (0.9)
Education	
Illiterate	413 (95.8)
Primary	8 (1.9)
Secondary, and above	10 (2.3)
O. viverrini infection intensity	
GM egg counts (EPG)	3961
Min–max of egg counts (EPG)	24–69,648
Intensity groups[a]	
Light (≤500 EPG)	173 (40.1)
Moderate (501–999 EPG)	63 (14.6)
Heavy (1000–1999 EPG)	61 (14.2)
Very heavy (≥2000 EPG)	134 (31.1)

Data are numbers and (%) of subjects.
[a] According to Sripa et al. (2009), based on Kato-Katz thick smear examinations; GM – geometric mean, EPG eggs per gram stool.

Table 2
Hepatobiliary morbidity in *Opisthorchis viverrini* infected adult patients ($n = 431$).

Morbidity		n (%)
Height of left liver lobe (cm)	Mean (SD)	5.4 (1.0)
Normal parenchyma	Grade 0	41 (9.5)
Mild periductal fibrosis	Grade 1+	30 (7.0)
Moderate periductal fibrosis	Grade 2+	287 (66.6)
Advanced periductal fibrosis	Grade 3+	73 (16.9)
Suspected lesion of CCA		5 (1.2)
Cirrhosis		3 (0.7)
Fatty liver		52 (12.1)
Length pre-fatty meal (cm)	Mean (95% CI)	6.9 (6.8–7.6)
Length, post-fatty meal (cm)	Mean (95% CI)	5.4 (5.3–5.5)
"Pre" minus "post" fatty meal (cm)		1.4
Gallbladder pathologies		
Wall thickness		5 (1.2)
Wall irregularity		2 (0.5)
Sludge present		6 (1.4)
Stone present		57 (13.2)
Bile duct dilated		10 (2.3)
Intra hepatic duct stone		1 (0.2)

Data are numbers; (%) of subjects, unless otherwise indicated.

2.5. Data management and analysis

Data were double-entered and validated in EpiData, version 3.1 (Epidata Association; Odense, Denmark). STATA software, version 10.1 (Stata Corp., College Station, TX, USA) was employed for analysis. *O. viverrini* infections were grouped according to light (1–500 eggs per gram stool [EPG]), moderate (501–999 EPG), heavy (1000–1999 EPG) and very heavy (≥2000 EPG) infection intensity (Sripa et al., 2009). Descriptive statistics were used (counts, percentages, and means and standard deviations [SD]). A Kruskal Wallis rank test was used to compare egg counts of *O. viverrini* among patients with liver lobe enlargement to patients without this condition. In addition, ANOVA, logistic regression and multinomial logistic regression were used. A *P-value* below 5% was considered significant.

3. Results

3.1. Patient characteristics

US examination was performed on 431 *O. viverrini* infected adults (Fig. 1). Enrolled patients had a mean age of 43 years (range 20–86 years (Table 1). There were more women (57.8%) than men (42.2%). Most patients were farmers (99.1%) and illiterate (95.8%, i.e. did not attend primary school). Patients' mean weight was 50 kg. Overall, *O. viverrini* geometric mean egg counts were 3961 EPG (range 24–69,648 EPG). Almost one third (31.1%) of the study participants had a very heavy *O. viverrini* infection intensity; 40.1%, 14.7% and 14.2% had a light, moderate, and heavy *O. viverrini* infection intensity, respectively.

Many patients reported abdominal discomfort (75.6%), pain in the right upper quadrant (RUQ, 60.5%), and experiencing "hot sensations" around the RQU (25.0%). Two patients had jaundice and reporting skin itching. Almost all patients (99.5%) had consumed *Pa dek* (raw fermented fish sauce) within the last week; three quarters (75.8) reported regular consumption of raw *Koy-pa* and *Lap-pa* (meals containing raw fish). Twenty-five per cent reported having a latrine at home but fewer than 5% use it regularly. The others defecate outside.

3.2. Hepatobiliary morbidity

Only 41 patients (9.5%) had a normal liver parenchyma (Table 2). In 66.6% and 16.9% of the patients moderate and advanced periductal fibrosis was diagnosed (Fig. 2), respectively. Gallbladder abnormalities were observed, such as wall thickening (1.2%), gallbladder wall irregularities (0.5%), and sludge (1.4%). Gallbladder stones were observed frequently (13.2%). Other pathologies were diagnosed in considerable frequencies: fatty liver (12.1%), kidney stones (8.5%), kidney cysts (7.8%), and cirrhosis-like condition (0.7%).

We did not identify any statistically significant association between the *O. viverrini* infection intensity and the left liver lobe enlargement ($P = 0.27$), gallbladder retraction capacity after meal (length difference pre- and post-fatty meal, $P = 0.46$), grade of periductal fibrosis ($P = 0.38$), presence of gall stones ($P = 0.44$) and other pathogenesis (i.e. kidney stone(s) and kidney cyst(s), $P > 0.05$).

3.3. Prevalence of suspected CCA

US assessment revealed five patients with liver masses suggestive of CCA, representing 1.2% of the random sample of *O. viverrini* infected patients (Tables 2 and 3). These patients (two men and three women) had a mean age of 52 years (range 36–67 years). They were referred to the regional hospital in Pakse (Champasack province) for further examinations.

Two patients had a heavy and 3 had a very heavy *O. viverrini* infection intensity (Table 3). All cases had normal liver function tests (AST, ALT, ALP, bilirubine), except for one male and one female patient with markedly increased levels of AST, ALT, ALP (248.16 mg/dl) and bilirubine (13.3 g/l), indicating that pathology may develop to prevent the biliary circulation (Figs. 3 and 4). Hepatitis B or C was not diagnosed in any of these patients. Tumour markers from all five patients, namely IL-6, PAI and CA19-9, were in a normal range.

Table 3
Follow-up examination of five patients suggestive for cholangiocarcinoma.

	Case 1	Case 2	Case 3	Case 4	Case 5
Age (years), sex	65, m	48, m	36, f	45, f	67, f
Liver function tests					
AST(IU/l)	127	39	49	30	23
ALT (IU/l)	47.0	35.9	45.9	37.9	16.7
Total bilirubine	13.3	8.6	9.5	8.5	10.3
Albumin (g/l)	6.8	4.9	5.3	4.3	3.3
ALP	248.2	75.2	55.4	65.3	74.8
GGT (IU/l)	147.9	10.3	6.8	9.9	8.8
AFP (IU/l)	0.8	0.3	0.2	0.04	0.1
Creatinine (mg/dl)	1109	173	1335	1128	1278
Tumour markers					
IL-6 (pg/ml)	<10	<10	<10	11.5	<10
PAI (pg/ml)	5700	14,250	9891	19,841	16,827
CA 19-9	25.0	20.0	12.9	12.8	14.6
Infections					
O. viverrini (EPG) (infection intensity)	1968 (heavy)	2878 (very heavy)	3734 (very heavy)	1752 (heavy)	4680 (very heavy)
HBsAg-HBV	Neg.	Neg.	Neg.	Neg.	Neg.
HCsAg-HCV	Neg.	Neg.	Neg.	Neg.	Neg.
Reported ill health	Intense jaundice	Absence	Absence	Absence	Moderate jaundice, mass palpable at RUQ
Ultrasonography findings	Liver categorised as grade 2+, liver mass in segment 7, markedly dilated bile duct, intrahepatic stones	Liver categorised as grade 2+, liver mass in segment 5, no dilated bile duct detected	Liver categorised as grade 3+, liver mass in segment 5, no dilated bile duct detected	Liver categorised as grade 2+, fatty liver mass presence in segment 5 of liver, no dilated bile duct detected, left hydronephosis	Liver categorised as grade 3+, fatty liver mass presence in segment 5 of liver, no dilated bile duct detected

Data are no; (%) of subject; EPG, eggs per gram of faeces, ALT alanine aminotransferase; AFP Alpha fetoprotein; ALP alkaline phosphatase; AST aspartate aminotransferase; CA 19-9 Carbohydrate antigen 19-9; f female; GGT Gamma-glutamyl transferase (GGT); HBsAg hepatitis B surface antigen; HCsAg hepatitis C surface antigen; IL-6 Interleukin 6, m male; neg. negative; PAI plasminogen activator inhibitor; RUQ right upper quadrant.

4. Discussion

We assessed the degree of hepatobiliary pathology including lesions suggestive of CCA in rural communities in Southern Laos where *O. viverrini* infection is highly endemic. Among a random sample of infected adults in 10 villages, we identified very high prevalence rates of clinical hepatobiliary pathology – 83.5% had moderate or advanced periportal fibrosis, while only 9.5% of the participants had a normal liver parenchyma. Fatty liver was very frequent (12.5%). In five patients representing 1.2% of the sample lesions suggestive for CCA were observed. This is the first study reporting on suspected CCA lesions in communities in Laos.

O. viverrini infection leads to a variety of hepatobiliary diseases (Mairiang et al., 2006, 2012; Sayasone et al., 2012) ranging from non-specific or asymptomatic cases, such as upper right quadrant pain or abdominal irritations to severe manifestations, namely cholangitis, obstructive jaundice, cholelithiasis, gall stones and liver periductal fibrosis, and the most severe outcome of a chronic infection, a fatal CCA. The clinical manifestations in our study were similar to previously reported manifestations. However, they were diagnosed with a much higher prevalence. In our study, advanced periportal fibrosis was seen in 83.5% of participants, which is three times higher compared with reports from neighbouring Thailand (Mairiang and Mairiang, 2003; Sripa et al., 2009). Also, gallbladder and intrahepatic stones, bile duct dilatation and fatty livers were diagnosed in high frequencies. Of note, nine of the identified study participants could not be enrolled due to their precedent cholecystectomy. This fact further underlines the excess hepatobiliary morbidity burden in our study setting. The heavy *O. viverrini* infection intensity in our setting is the most likely explanation for the high level of morbidity. The mean infection intensity of 3961 EPG was observed and 45% of the patients had heavy or very heavy infection intensity. The *O. viverrini* infection intensity found in Saravan was twice as high as that found in Khon Kean (Mairiang et al., 2006; Sripa et al., 2009), where the incidence of CCA has been recorded as the highest in the world (Sripa et al., 2009; Vatanasapt et al., 1990).

In our study the infection status of the patients was assessed by the Kato-Katz technique. It is known that this technique is not able to distinguish between *O. viverrini* and MIF infection such as *H. taichui*. Therefore, it is possible that some of infections classified as *O. viverrini* are MIF. However, in a previous study we confirmed the presence of *O. viverrini* in our study area by demonstrating adult *O. viverrini* flukes in human stools and by formalin-ethyl acetate concentration technique (FECT) (Sayasone et al., 2009). In latter technique the *O. viverrini* eggs can be distinguished from eggs of MIF (Elkins et al., 1991).

O. viverrini infection has been classified as a Group 1 carcinogen agent, triggering CCA development (Bouvard et al., 2009; IARC, 1994). In Northeast Thailand an estimated 60% of CCA cases are due to *O. viverrini* infection (Mairiang et al., 2006; Sripa et al., 2007). Mechanisms include direct irritations from the fluke in the bile ducts and immunopathological pathways of the parasite toxins. In addition, recent studies suggest an influence of *Opisthorchis* infection on the host intestinal and biliar microbiome (Plieskatt et al., 2013). Nonetheless, CCA has multi-factorial origins (Mairiang et al., 2006; Shin et al., 2010). Various additional stressors like *N*-nitrosamide from foodstuff such as fermented fish sauce “Pa dek” (Sithithaworn et al., 2014; Sripa et al., 2012) have been identified as important determinants. All the stressors reported in Thailand are also highly prevalent in our study setting in Southern Laos.

The world’s highest CCA prevalence and incidence has been recorded in Northeast Thailand where 71% of primary cancers are

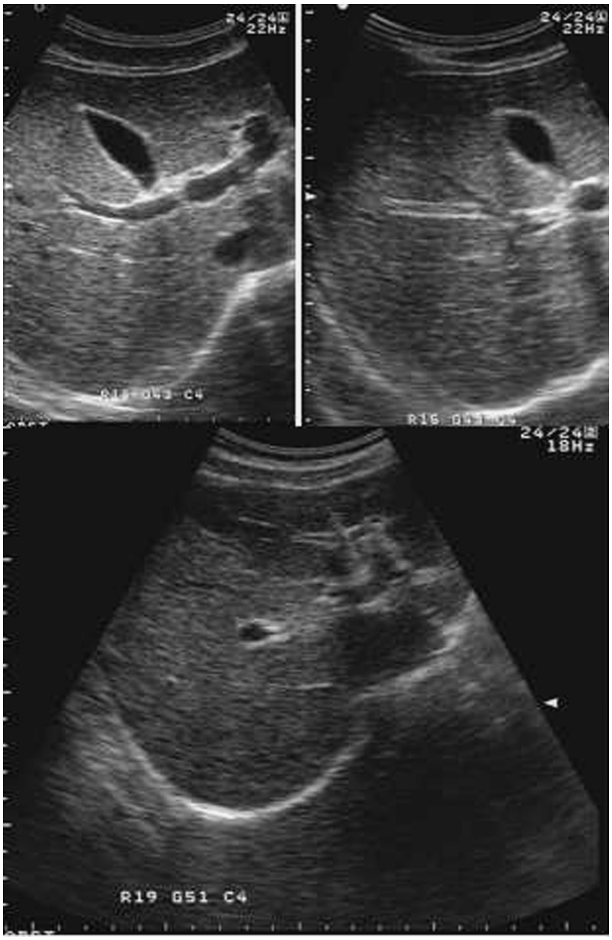

Fig. 2. Liver parenchyma fibrosis observed: (1+) starry sky (top, left); (2+) rings and pipe stems (top, right); (3+) highly echogenic 'patches' extending to peripheral (bottom) areas.

CCA (Sripa et al., 2007, 2011). In our study, of 431 subjects five patients had lesions suggestive of CCA. The suspected diagnosis was based on invasively growing liver masses. They were typical of those seen in confirmed CCA patients. Furthermore, all patients had heavy or very heavy *O. viverrini* infection intensity. All five cases reported other ill health problems such as high blood creatinine level, and had abnormal liver function tests. Latter are contraindications for performing further diagnostic procedures such as e.g. endoscopic retrograde cholangiopancreatography (ERCP). Hence, no further targeted diagnosis could be performed. All had advanced periductal fibrosis (case 1, 5 = Grade 3+: case 2, 3, 4 = Grade 2+). These observations are typical signs in CCA patients in Thailand (Mairiang and Mairiang, 2003; Mairiang et al., 2006).

Liver function tests performed on suspected CCA patients were within the normal range. In CCA patients, liver function is preserved over a long period in illness (Feldman et al., 2013). The proposed tumour markers for CCA, i.e. IL-6, CA 19-9 and PAI were also in the normal range. Biochemical test are not specific for CCA, except for CEA and CA19-9 (Bloom et al., 1999; Sharma and Ahuja, 1999). A recent case control study focused on the parasite-specific interleukin-6 (IL-6), a promising marker in detecting the pathogenesis of advanced periductal fibrosis in individuals infected with chronic *O. viverrini* (Sripa et al., 2009). Given that these markers measure advanced CCA, their values indicate that our cases might still be in the early stages of development.

In the absence of further diagnostic result, we cannot definitively conclude on a CCA diagnosis. Hence, these suspected CCA lesions are currently the best available information on CCA in communities in Laos. In a recent ultrasonographical hepatobiliary morbidity survey in Champasack province, a similar prevalence of 1% (8 among 800 examined) suspected CCA lesions were recorded (pers. communication, Dr. Bouasy Hongvanthong). These findings warrant further investigations to confirm CCA and to assess its frequency in the different provinces of the country, particularly in Southern Laos, where infection with *O. viverrini* have been documented (Chai et al., 2005, 2007, 2013; Sayasone et al., 2012).

We noted nephropathy, such as kidney stones (8.6%), kidney cyst(s) (7.9%), and hydronephosis (1.6%), in our study population. Only kidney cysts showed an association with APF. This finding is consistent with recent reports on kidney pathogenesis observed in hamsters infected with *O. viverrini* (Boonpucknavig et al., 1992; Saichua et al., 2013).

CCA diagnosis is not possible in Laos due to the lack of diagnostic (i.e. imaging techniques, liver biopsy) and adequate case management facilities. Suspected cases diagnosed in hospitals in Vientiane and elsewhere in Laos are referred to hospitals in Thailand, particularly to Khon Kaen University hospital. In our study, a relatively small number of participants were included. As *O. viverrini* infections occur in clusters, larger studies in settings with varying prevalence rates are required to assess the region-specific morbidity and cancer risks, and hence to assess the Lao morbidity burden. However, mass lesions could have resulted from other common malignant hepatobiliary, namely hepatocarcinoma (hepatitis B and C viruses) (Shin et al., 1996). Furthermore, there is evidence that other predisposing factors for CCA, like malformations and life style, may play a role (Gatto et al., 2010; Shin et al., 2010). Differential diagnosis of morbidity must thus be investigated in more detail in future studies.

This study is a first attempt to quantify mild to severe morbidity including the presence of malignant lesions in rural communities in Laos, where the prevalence of *O. viverrini* infection is high. The

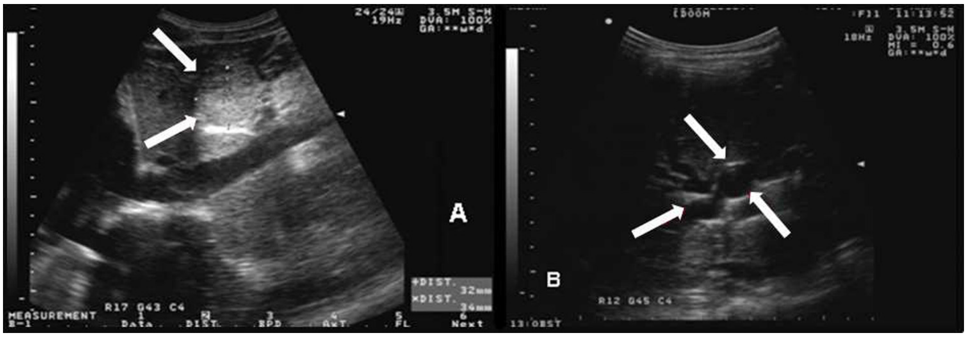

Fig. 3. Patient (male, age 65 year): solid mass lesion with well-defined contour in right liver lobe (left); mechanical bile duct dilatation due to the mass (right).

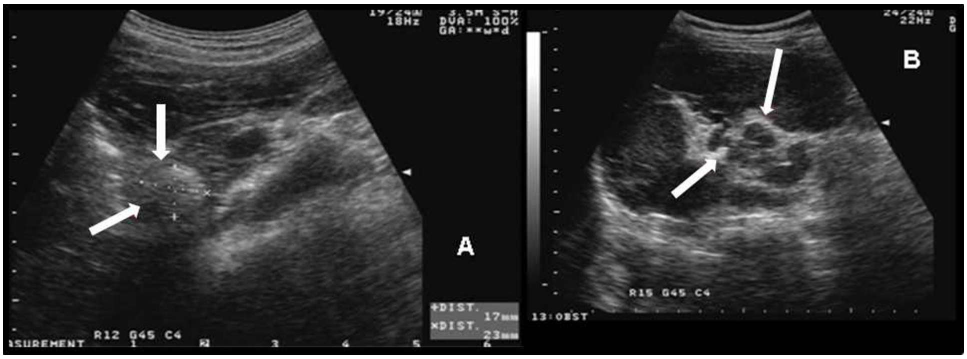

Fig. 4. Patient (female, age 67 year): mass lesion in liver with solid mass lesion with well-defined contour in right liver lobe (left); bile duct dilatation (right).

absence of a conclusive diagnosis for the severe sequelae hampers the estimation of the CCA burden in Laos. Furthermore, the facilities for advanced and less invasive CCA diagnostic procedures, such as ERCP, magnetic resonance imaging (MRI) and magnetic resonance cholangiopancreatography (MRCP), are needed to fully assess the burden of hepatobiliary morbidities in *O. viverrini*-endemic settings in Laos.

Acknowledgements

The authors would like to thank the study participants for their active participation. We thank Dr. Eimorn Mairiang from Department of Radiology, Faculty of Medicine, University of Khon Kaen, Khon Kean, Thailand for her assistance with ultrasound images. We also thank the Saravan provincial Health Departments and District Health Office for excellent assistance. We are grateful to Dr. Remigio Olveda from the Research Institute for Tropical Medicine, Philippines (RTM-DOH) and Dr. Somphou Sayasone from the National Institute of Public Health for comments and advice on an initial draft of manuscript and proposal. This study was funded by the UNDP/WB/WHO Special Grants Programme for Operational Research in Communicable Diseases, Manila, Philippines (#2010/92660-0). PAS received additional support by the Rudolf Geigy Foundation and the City of Basel. We thank Mrs Amena Briet for her efficient English editing of the manuscript.

References

Blechacz, B., Gores, G.J., 2008. Cholangiocarcinoma: advances in pathogenesis, diagnosis, and treatment. Hepatology 48, 308–321.

Bloom, C.M., Langer, B., Wilson, S.R., 1999. Role of US in the detection, characterization, and staging of cholangiocarcinoma. Radiographics 19, 1199–1218.

Boonpucknavig, S., Boonpucknavig, V., Tanvanich, S., Doungchawee, G., Thamavit, W., 1992. Development of immune-complex glomerulonephritis and amyloidosis in Syrian golden hamsters infected with *Opisthorchis viverrini*. J. Med. Assoc. Thail. 75 (Suppl. 1), 7–19.

Bouvard, V., Baan, R., Straif, K., Grosse, Y., Secretan, B., El, G.F., Benbrahim-Tallaa, L., Guha, N., Freeman, C., Galichet, L., Cogliano, V., Sripa, B., 2009. A review of human carcinogens—Part B: Biological agents Concerted action is needed to tackle liver fluke infections in Asia. Lancet Oncol. 10, 321–322.

Chai, J.Y., Han, E.T., Guk, S.M., Shin, E.H., Sohn, W.M., Yong, T.S., Eom, K.S., Lee, K.H., Jeong, H.G., Ryang, Y.S., Hoang, E.H., Phommasack, B., Insisiengmay, B., Lee, S.H., Rim, H.J., 2007. High prevalence of liver and intestinal fluke infections among residents of Savannakhet province in Laos. Korean J. Parasitol. 45, 213–218.

Chai, J.Y., Park, J.H., Han, E.T., Guk, S.M., Shin, E.H., Lin, A., Kim, J.L., Sohn, W.M., Yong, T.S., Eom, K.S., Min, D.Y., Hwang, E.H., Phommmasack, B., Insisiengmay, B., Rim, H.J., 2005. Mixed infections with *Opisthorchis viverrini* and intestinal flukes in residents of Vientiane municipality and Saravane province in Laos. J. Helminthol. 79, 283–289.

Chai, J.Y., Yong, T.S., Eom, K.S., Min, D.Y., Jeon, H.K., Kim, T.Y., Jung, B.K., Sisabath, L., Insisiengmay, B., Phommasack, B., Rim, H.J., 2013. Hyperendemicity of *Haplorchis taichui* infection among riparian people in Saravane and Champasak province, Lao PDR. Korean J. Parasitol. 51, 305–311.

Elkins, D.B., Sithithaworn, P., Haswell-Elkins, M., Kaewkes, S., Awacharagan, P., Wongratanacheewin, S., 1991. *Opisthorchis viverrini*: relationships between egg counts, worms recovered and antibody levels within an endemic community in northeast Thailand. Parasitology 102, 283–288.

Feldman, M., Friedman, L.S., Lawrence, J., 2013. Sleisenger and Fordtran's Gastrointestinal and Liver Disease, 8th ed., pp. 1493–1496.

Forrer, A., Sayasone, S., Vounatsou, P., Vonghachack, Y., Bouakhasith, D., Vogt, S., Glaser, R., Utzinger, J., Akkhavong, K., Odermatt, P., 2012. Spatial distribution of, and risk factors for, *Opisthorchis viverrini* infection in southern Lao PDR. PLoS Negl. Trop. Dis. 6, e1481.

Gatto, M., Bragazzi, M.C., Semeraro, R., Napoli, C., Gentile, R., Torrice, A., Gaudio, E., Alvaro, D., 2010. Cholangiocarcinoma: update and future perspectives. Dig. Liver Dis. 42, 253–260.

IARC, 1994. Schistosomiasis, liver fluke and helicobacter pylori. In: IARC Working Group on the evaluation of carcinogenic risks to humans, Lyon, 7-4 June 1994. 61 ed, pp. 1–241.

Katz, N., Chaves, A., Pellegrino, J., 1972. A simple device for quantitative stool thick-smear technique in schistosomiasis mansoni. Rev. Inst. Med. Trop. São Paulo 14, 397–400.

Keiser, J., Utzinger, J., 2009. Food-borne trematodiases. Clin. Microbiol. Rev. 22, 466–483.

Mairiang, E., Chaiyakum, J., Chamadol, N., Laopaiboon, V., Srinakarin, J., Kunpitaya, J., Sriamporn, S., Suwanrungruang, K., Vatanasapt, V., 2006. Ultrasound screening for *Opisthorchis viverrini*-associated cholangiocarcinomas: experience in an endemic area. Asian Pac. J. Cancer Prev. 7, 431–433.

Mairiang, E., Laha, T., Bethony, J.M., Thinkhamrop, B., Kaewkes, S., Sithithaworn, P., Tesana, S., Loukas, A., Brindley, P.J., Sripa, B., 2012. Ultrasonography assessment of hepatobiliary abnormalities in 3359 subjects with *Opisthorchis viverrini* infection in endemic areas of Thailand. Parasitol. Int. 61, 208–211.

Mairiang, E., Mairiang, P., 2003. Clinical manifestation of opisthorchiasis and treatment. Acta Trop. 88, 221–227.

MOH, 2004. Diagnosis and Treatment at the District. A Diagnosis and Treatment Guideline for the District Hospital in Lao PDR. Ministry of Health, Vientiane.

Niamey Working Group, 2000. Ultrasound in Schistosomiasis, a Practical Guide to the Standardized Use of Ultrasonography for the Assessment of Schistosomiasis Related Morbidity, World Health Organization/TDR/SCH/ULTRASON/document, Geneva.

Phongluxa, K., Xayaseng, V., Vonghachack, Y., Akkhavong, K., van, E.P., Odermatt, P., 2013. Helminth infection in Southern Laos: high prevalence and low awareness. Parasit. Vectors 6, 328.

Plieskatt, J.L., Deenonpoe, R., Mulvenna, J.P., Krause, L., Sripa, B., Bethony, J.M., Brindley, P.J., 2013. Infection with the carcinogenic liver fluke *Opisthorchis viverrini* modifies intestinal and biliary microbiome. FASEB J. 27, 4572–4584.

Rim, H.J., Chai, J.Y., Min, D.Y., Cho, S.Y., Eom, K.S., Hong, S.J., Sohn, W.M., Yong, T.S., Deodato, G., Standgaard, H., Phommasack, B., Yun, C.H., Hoang, E.H., 2003. Prevalence of intestinal parasite infections on a national scale among primary schoolchildren in Laos. Parasitol. Res. 91, 267–272.

Saichua, P., Sithithaworn, P., Jariwala, A.R., Diemert, D.J., Sithithaworn, J., Sripa, B., Laha, T., Mairiang, E., Pairojkul, C., Periago, M.V., Khuntikeo, N., Mulvenna, J., Bethony, J.M., 2013. Microproteinuria during *Opisthorchis viverrini* infection: a biomarker for advanced renal and hepatobiliary pathologies from chronic opisthorchiasis. PLoS Negl. Trop. Dis. 7, e2228.

Sayasone, S., Mak, T.K., Vanmany, M., Rasphone, O., Vounatsou, P., Utzinger, J., Akkhavong, K., Odermatt, P., 2011. Helminth and intestinal protozoa infections, multiparasitism and risk factors in Champasack province, Lao People's Democratic Republic. PLoS Negl. Trop. Dis. 5, e1037.

Sayasone, S., Odermatt, P., Phoumindr, N., Vongsaravane, X., Sensombath, V., Phetsouvanh, R., Choulamany, X., Strobel, M., 2007. Epidemiology of *Opisthorchis viverrini* in a rural district of Southern Lao PDR. Trans. R. Soc. Trop. Med. Hyg. 101, 40–47.

Sayasone, S., Rasphone, O., Vanmany, M., Vounatsou, P., Utzinger, J., Tanner, M., Akkhavong, K., Hatz, C., Odermatt, P., 2012. Severe morbidity due to *Opisthorchis viverrini* and *Schistosoma mekongi* infection in Lao People's Democratic Republic. Clin. Infect. Dis. 55, e54–e57.

Sayasone, S., Vonghajack, Y., Vanmany, M., Rasphone, O., Tesana, S., Utzinger, J., Akkhavong, K., Odermatt, P., 2009. Diversity of human intestinal helminthiasis in Lao PDR. Trans. R. Soc. Trop. Med. Hyg. 103, 247–254.

Sharma, M.P., Ahuja, V., 1999. Aetiological spectrum of obstructive jaundice and diagnostic ability of ultrasonography: a clinician's perspective. Trop. Gastroenterol. 20, 167–169.

Shin, H.R., Lee, C.U., Park, H.J., Seol, S.Y., Chung, J.M., Choi, H.C., Ahn, Y.O., Shigemastu, T., 1996. Hepatitis B and C virus, *Clonorchis sinensis* for the risk of liver cancer: a case-control study in Pusan, Korea. Int. J. Epidemiol. 25, 933–940.

Shin, H.R., Oh, J.K., Masuyer, E., Curado, M.P., Bouvard, V., Fang, Y.Y., Wiangnon, S., Sripa, B., Hong, S.T., 2010. Epidemiology of cholangiocarcinoma: an update focusing on risk factors. Cancer Sci. 101, 579–585.

Sithithaworn, P., Andrews, R.H., Nguyen, V.D., Wongsaroj, T., Sinuon, M., Odermatt, P., Nawa, Y., Liang, S., Brindley, P.J., Sripa, B., 2012. The current status of opisthorchiasis and clonorchiasis in the Mekong Basin. Parasitol. Int. 61, 10–16.

Sithithaworn, P., Yongvanit, P., Duenngai, K., Kiatsopit, N., Pairojkul, C., 2014. Roles of liver fluke infection as risk factor for cholangiocarcinoma. J. Hepatobiliary Pancreat. Sci. 21, 301–308.

Soukhathammavong, P., Odermatt, P., Sayasone, S., Vonghachack, Y., Vounatsou, P., Hatz, C., Akkhavong, K., Keiser, J., 2011. Efficacy and safety of mefloquine, artesunate, mefloquine-artesunate, tribendimidine, and praziquantel in patients with *Opisthorchis viverrini*: a randomised, exploratory, open-label, phase 2 trial. Lancet Infect. Dis. 11, 110–118.

Sripa, B., 2008. Concerted action is needed to tackle liver fluke infections in Asia. PLoS Negl. Trop. Dis. 2, e232.

Sripa, B., Bethony, J.M., Sithithaworn, P., Kaewkes, S., Mairiang, E., Loukas, A., Mulvenna, J., Laha, T., Hotez, P.J., Brindley, P.J., 2011. Opisthorchiasis and *Opisthorchis*-associated cholangiocarcinoma in Thailand and Laos. Acta Trop. 120 (Suppl. 1), S158–S168.

Sripa, B., Brindley, P.J., Mulvenna, J., Laha, T., Smout, M.J., Mairiang, E., Bethony, J.M., Loukas, A., 2012. The tumorigenic liver fluke *Opisthorchis viverrini* – multiple pathways to cancer. Trends Parasitol. 28, 395–407.

Sripa, B., Kaewkes, S., Sithithaworn, P., Mairiang, E., Laha, T., Smout, M., Pairojkul, C., Bhudhisawasdi, V., Tesana, S., Thinkamrop, B., Bethony, J.M., Loukas, A., Brindley, P.J., 2007. Liver fluke induces cholangiocarcinoma. PLoS Med. 4, e201.

Sripa, B., Mairiang, E., Thinkhamrop, B., Laha, T., Kaewkes, S., Sithithaworn, P., Tessana, S., Loukas, A., Brindley, P.J., Bethony, J.M., 2009. Advanced periductal fibrosis from infection with the carcinogenic human liver fluke *Opisthorchis viverrini* correlates with elevated levels of interleukin-6. Hepatology 50, 1273–1281.

Vatanasapt, V., Tangvoraphonkchai, V., Titapant, V., Pipitgool, V., Viriyapap, D., Sriamporn, S., 1990. A high incidence of liver cancer in Khon Kaen Province, Thailand. Southeast Asian J. Trop. Med. Public Health 21, 489–494.
