# Supplementary material for: Periductal Fibrosis and Cholangiocarcinoma-Related Outcomes in Liver Fluke-Endemic Regions: A Systematic Review and Meta-Analysis
Source: Med Sci (Basel). 2026 Jul 9;14(3):380. doi: 10.3390/medsci14030380 (PMC13414246; doi:10.3390/medsci14030380)
Supplement: Supplementary file 1 [file medsci-14-00380-s001.zip › included papers/Thanakijsombat et al., 2024.pdf]

RESEARCH ARTICLE

Editorial Process: Submission:07/08/2024 Acceptance:12/18/2024

Sonographic Predictors for Developing Cholangiocarcinoma: A Cohort Study from an Endemic Area

Natcha Thanakijsombat[1,2], Kamonwan Soonklang[3], Pantajaree Hiranrat[1], Poemporn Limpisook[1], Surachate Siripongsakun[1]*

Abstract

Background and Aim: Cholangiocarcinoma (CCA) is an aggressive malignancy with a poor prognosis. Bile duct and peribiliary changes related to CCA may present on ultrasound (US) findings. This study aims to evaluate US findings that could be used as predictors for developing CCA through our surveillance program in an endemic area of Thailand. **Methods:** The study population was 4,337 villagers in Northern Thailand with a 5-year abdominal US surveillance. Patient demographics data and ultrasound findings of calcifications/granulomas, periductal fibrosis, and diffuse bile duct dilatation were included. A logistic regression model was used to determine significant predictors. **Results:** There were 4,225 people included with an average age of 45.49±7.66 years. Prevalence of calcifications/granulomas, periductal fibrosis, and diffuse bile duct dilatation detected on baseline sonographic surveillance was 11.7%, 20.5%, and 11.3%, respectively. The univariate analysis for significant predictors for CCA include age (Relative Risk; RR = 1.12), family history of CCA (RR = 2.29), periductal fibrosis (RR=2.38), and diffuse bile duct dilatation (RR = 7.59). The multivariate analysis the independent predictors were age (RR = 1.12), family history of CCA (RR = 1.92), and diffuse bile duct dilatation (RR = 5.94), respectively. **Conclusions:** The sonographic predictor for CCA surveillance in endemic areas is diffuse bile duct dilatation. Age and family history of CCA are also helpful clinical markers.

Keywords: Cholangiocarcinoma- predictor- sonography- surveillance

Asian Pac J Cancer Prev, **25 (12)**, 4229-4236

Introduction

Cholangiocarcinoma (CCA) stands as the most prevalent primary malignancy of the biliary tract, originating from the biliary epithelial cells. Its etiology is closely linked to chronic inflammation and cholestasis within the biliary system. In the context of mortality statistics in the Thai population, CCA has persistently held sway for decades. CCA emerges as a particularly significant contributor, accounting for approximately 14,000 deaths annually nationwide, with over 50% of cases concentrated in the North and Northeastern regions of Thailand. Numerous studies in the past have explored the association between liver flukes and CCA etiology, leading to confirmation by the World Health Organization (WHO) of the pivotal role played by Liver Flukes such as Opisthorchis Viverrini (OV) and Clonorchis sinensis (CS) in the development of CCA [1]. The natural course of CCA is characterized by aggressiveness, with a median survival of fewer than 24 months post-diagnosis and a dismal 5-year survival rate of less than 5% [2]. Consequently, CCA presents a formidable challenge, particularly in its advanced stages, with early detection, diagnosis, and treatment remaining elusive even for experts in the field.

CCA incidence rates exhibit significant variation worldwide, likely due to differences in local risk factors and genetic predispositions. Southeast Asia, particularly Thailand, bears the highest burden of the disease, with prevalence notably concentrated in the northeastern and northern regions [3]. In response to the severity of the disease, Thailand initiated the "No raw fish consumption" campaign in endemic areas in 1987, aiming to mitigate risk associated with liver fluke ingestion from uncooked fish. Despite the campaign's success, the incidence rate has only seen a minimal decline, attributed in part to patients presenting at advanced stages of the disease when tumors are clinically silent in early stages [4]. To address this, Thailand's Ministry of Health implemented the Cholangiocarcinoma Screening and Care Program (CASCAP), a targeted endemic screening initiative using abdominal sonography. Results from 2019 affirm its effectiveness in early detection of operable CCA in high-risk areas [5].

Surveillance for cholangiocarcinoma (CCA) still

*[1]Sonographer School, Faculty of Health Science Technology, Chulabhorn Royal Academy, Bangkok, Thailand. [2]Department of Emergency Medicine, Police General Hospital, Bangkok, Thailand. [3]Data Management Unit, HRH Princess Chulabhorn College of Medical Science, Chulabhorn Royal Academy, Bangkok, Thailand. *For Correspondence: surachate.sir@cra.ac.th*

primarily relies on ultrasound due to the limited sensitivity of tumor markers and liver function tests, such as CA 19-9, CEA, bilirubin, and alkaline phosphatase, which often detect only advanced stages of cancer [6]. In contrast, ultrasound is an affordable and widely accessible tool capable of identifying pre-cancerous conditions and early-stage cholangiocarcinoma [6], thereby potentially improving survival outcomes through timely complete margin negative surgical resection after detection [7]. Another aspect of concern is the economic aspect of considering large-scale surveillance campaign of the country. A study indicates that ultrasound-based surveillance for cholangiocarcinoma in an endemic area of Thailand is cost-effective. The cost-effectiveness of such surveillance is influenced by disease-specific factors, primarily the prevalence of cholangiocarcinoma, and economic factors, notably the cost of ultrasound examinations [8].

At present, there is no consensus on specific ultrasonographic findings that can reliably predict the onset of CCA [9, 10]. However, recent imaging studies have shown promise in identifying early premalignant CCA lesions, such as Biliary Intraepithelial Neoplasia (Bil-IN) and Intraductal Papillary Neoplasm of the Biliary Tract (IPNB), potentially in a curative resectable stage [11]. In the premalignant stage, particularly with IPNB, dysplastic changes may lead to mucin production, resulting in biliary system dilatation without detectable mass obstruction [12]. The observation of biliary dilatation on ultrasound imaging suggests a potential association with premalignant CCA changes, a notion supported by a large cross-sectional CCA screening study that found a significant correlation between diffuse bile duct dilatation and CCA [13]. However, limited research data exists on imaging predictors that could serve as independent biomarkers for predicting CCA development, which could prove valuable for CCA surveillance. Thus, this study aims to evaluate ultrasound findings that could serve as predictors for CCA development within our surveillance program in an endemic area, Nan province, Thailand.

Materials and Methods

Study design and population

This historical cohort study enrolled residents of Ban Luang District, Nan Province, as the study population. Participants were aged between 30 and 60 years and were free from any cancer diagnosis or pregnancy at the time of recruitment. Village healthcare volunteers facilitated subject registration in collaboration with Banluang District Hospital, targeting a population of 6,327 individuals based on district census registration. Approval for this study was obtained from the Institutional Review Board and Ethics Committee for Human Research (Certification No. 0052/2661), and the requirement for individual consent was waived. The patient recruitment process is illustrated in Figure 1.

Study Procedures

1. Identification of study population and sample groups.

2. Construction of a case record form.

3. Collection of patient data from the project "Abdominal Ultrasound Surveillance for Cholangiocarcinoma Conducted in Banluang District, Nan Province, Thailand," conducted by the Banluang Hospital. Participants

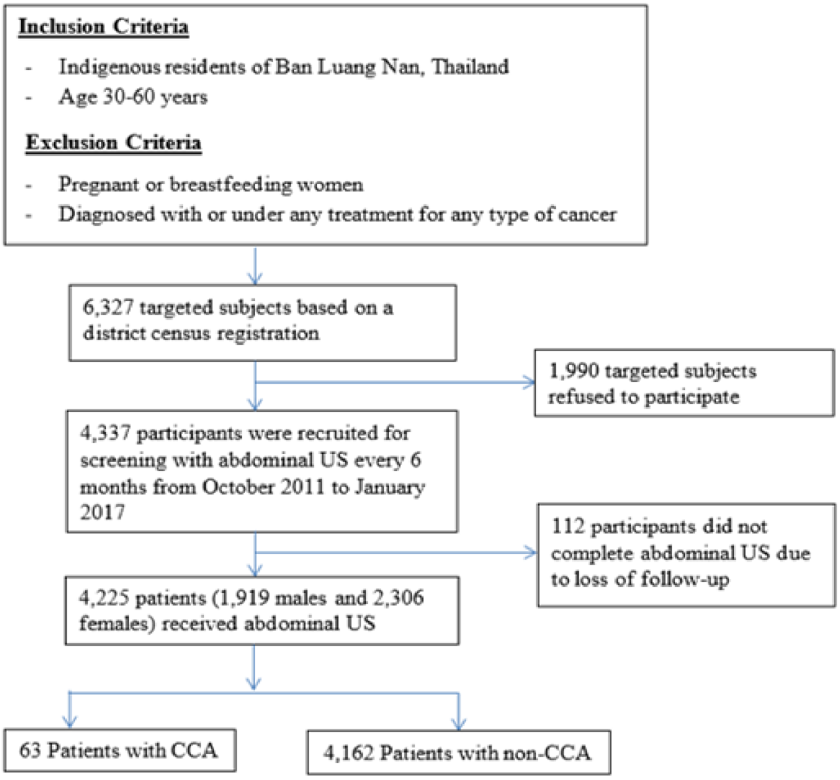

Figure 1. Schematic Diagram Illustrating Patient Inclusion and Exclusion in the Study.

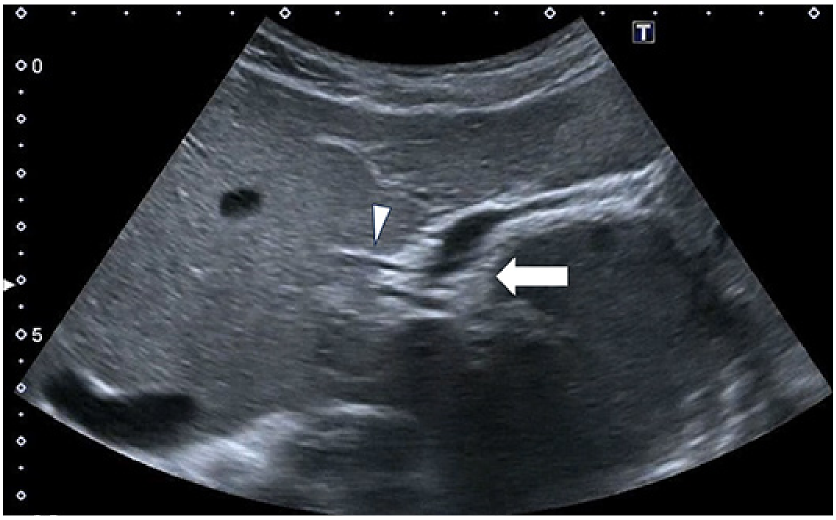

Figure 2. Sonographic Image Displaying Periductal Fibrosis Characterized by Increased Periportal Echogenicity (arrow), which often Accompanied by Borderline Bile Duct Dilatation (arrowhead).

consented to undergo abdominal ultrasound every 6 months for 5 years from October 2011 to January 2017. Medical records of all 4,337 participants were reviewed for detailed information, and all relevant ultrasound images, including those showing periductal fibrosis, diffuse bile duct dilatation, and liver calcifications/granulomas, were retrieved. Additionally, demographic data, including age, gender, and family history of first-degree relatives with CCA, were collected.

4. Statistical data analysis to identify ultrasonographic predictors.

Imaging techniques and definition of imaging findings

Imaging techniques

Ultrasound images utilized in this study were generated using the Aplio 300 ultrasonography unit from Toshiba Medical System Corporation, Tokyo, Japan. The imaging was conducted with a curvilinear probe operating at a frequency of 2-5 MHz, sourced from the Chulabhorn Medical Institute, and performed by board-certified radiologists.

Definition of imaging finding

1. Periductal fibrosis is characterized by an increase in periportal echo on sonography, resulting from thickening of the bile duct wall, which runs parallel to the portal vein [6] as shown in Figure 2.

2. Diffuse bile duct dilatation is defined as the dilatation of the intrahepatic bile duct when the diameter exceeds 3 mm or comprises more than 40% of the accompanying portal vein, without other associated abnormalities [14] as shown in Figure 3.

3. Calcifications/granulomas are characterized by hyperechoic nodules with a posterior acoustic shadow within the liver parenchyma as shown in Figure 4.

Statistical analysis

Demographic and anthropometric data of the patients,

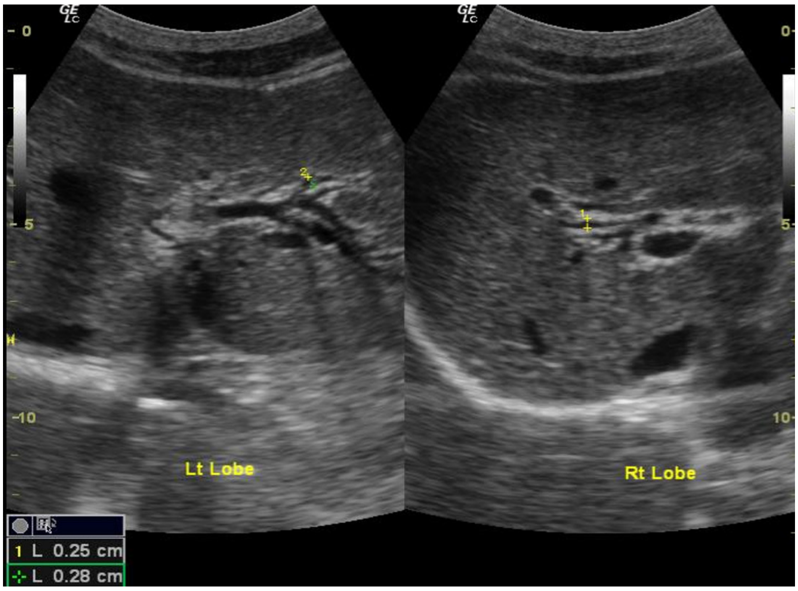

Figure 3. Sonographic Image Depicting Diffuse Bile duct Dilatation, Revealing Visible Tubular Structures adjacent to the Portal Vein of the Left and Right Hepatic Lobes, respectively.

Table 1. Demographic Data and Presence of key US Findings of the Patients

	CCA (63 cases)	Non- CCA (4,162 case)	Total (4225 case)	p value
Age (years)	51.41±5.85	45.40±7.65	45.49±7.66	<0.001
Gender				0.263
Male	33 (52.4%)	1,886 (45.31%)	1,919 (45.42%)	
Female	30 (47.6%)	2,276 (54.69%)	2,306 (54.58%)	
Family history of Cholangiocarcinoma*				0.002
No	44 (69.84%)	3,511 (84.36%)	3,555 (84.14%)	
Yes	19 (30.16%)	651 (15.64%)	670 (15.86%)	
Calcifications/granuloma				0.578
No	57 (90.48%)	3,671 (88.20%)	3,728 (88.24%)	
Yes	6 (9.52%)	491 (11.80%)	497 (11.76%)	
Diffuse bile duct dilatation				<0.001
No	32 (50.79%)	3,715 (89.26%)	3,747(88.69%)	
Yes	31 (49.21%)	447 (10.74%)	478 (11.31%)	
Periductal fibrosis				<0.001
No	39 (61.90%)	3,318 (79.72%)	3,357 (79.46%)	
Yes	24 (38.10%)	844 (20.28%)	868 (20.54%)	
Number of unresectable CCA	11 (17.4%)	NA		
Number of resectable CCA	41 (65.0%)	NA		
Number of premalignant CCA**	21 (33.3%)	NA		

including age, gender, and body mass index, as well as family history of CCA (first-degree relative), and sonographic findings including hepatic calcifications/granuloma, diffuse bile duct dilatation, and periductal fibrosis, were analyzed using chi-square and Student's t-test. Logistic regression analysis was employed to determine significant predictors for developing CCA, incorporating gender, age, family history of CCA, hepatic calcifications/granuloma, diffuse bile duct dilatation, and periductal fibrosis. Multivariate analysis was performed with forward stepwise selection to identify independent predictive variables with a significance level of $p < 0.05$. The analysis was conducted using Stata version 12, from Stata Corporation, College Station, and significance was defined as a two-sided p-value < 0.05.

Results

A total of 4,225 individuals were included in the study, comprising 1,919 males and 2,306 females, with an average age of 45.49 ± 7.66 years. The prevalence of calcifications, periductal fibrosis, and diffuse bile duct dilatation detected on baseline sonographic surveillance was 497 (11.76%), 868 (20.54%), and 478 (11.31%), respectively. Demographic data are presented in Table 1.

The univariate analysis for predictors of CCA

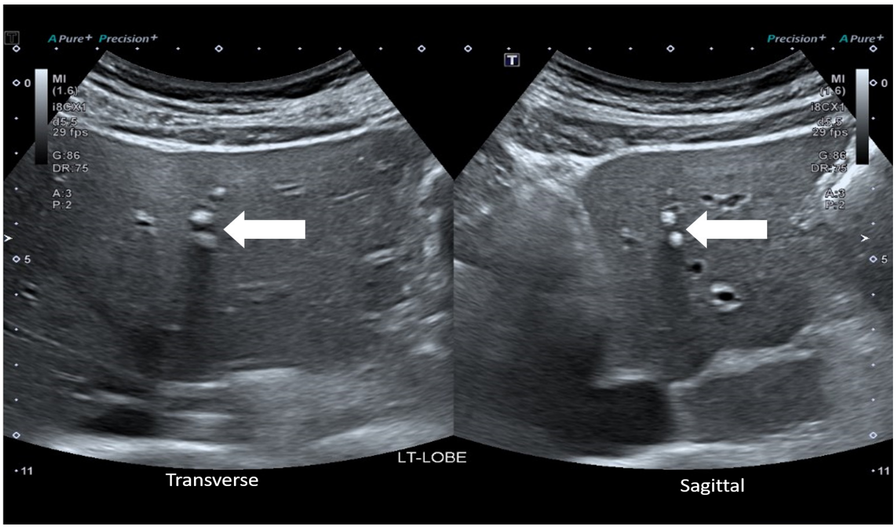

Figure 4. Sonographic Image Displaying a Calcified Granuloma Characterized by Hyperechoic Nodules with Posterior Acoustic Shadows (arrow).

Table 2. Univariate and Multivariate Analysis of CCA's Predictors

	Univariate Analysis			Multivariate Analysis		
	RR	95% CI	p-value	RR	95% CI	p-value
Age (years)	1.12	1.08-1.16	<0.001	1.12	1.08-1.16	<0.001
Gender (male vs female)	1.32	0.81-2.16	0.265			
Family history of CCA* (Yes vs No)	2.29	1.35-3.90	0.002	1.92	1.14-3.26	0.015
Calcifications/granuloma (Yes vs No)	0.79	0.34-1.32	0.58			
Diffuse bile duct dilatation (Yes vs No)	7.59	4.68-12.33	<0.001	5.94	3.65-9.66	<0.001
Periductal fibrosis (Yes vs No)	2.38	1.44-3.94	0.001			

* Family history of first-degree relative for CCA

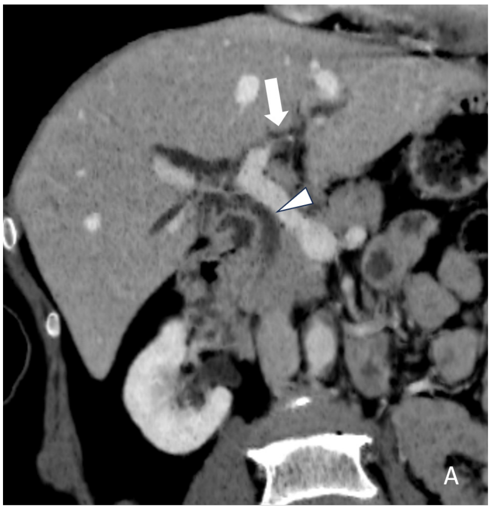

Figure 5a. Coronal Minimal Intensity Projection CT Image Depicting the Initial Confirmation of Diffuse Bile Duct Dilatation, Encompassing the Right and Left Intrahepatic Bile Duct (arrow) and Common Hepatic Bile Duct (arrowhead).

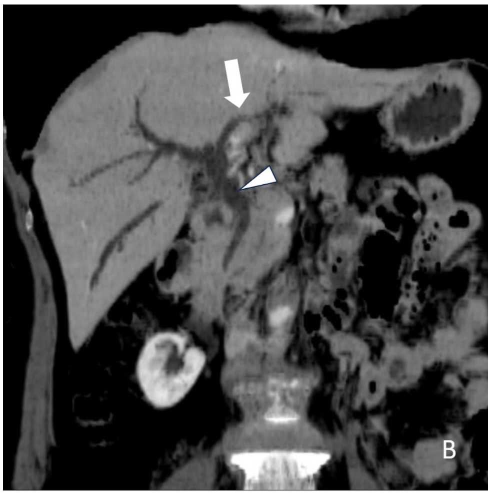

Figure 5b. Coronal Minimal Intensity Projection CT Image was Taken 1 Year after the Initial CT Scan, Illustrating Persistent Dilatation of the Biliary System.

development includes age (RR = 1.12, p < 0.001), family history of CCA (RR = 2.29, p = 0.002), periductal fibrosis (RR = 2.38, p = 0.001), and diffuse bile duct dilatation (RR = 7.59, p < 0.001). Multivariate analysis of independent predictors for CCA development revealed age (RR = 1.12,

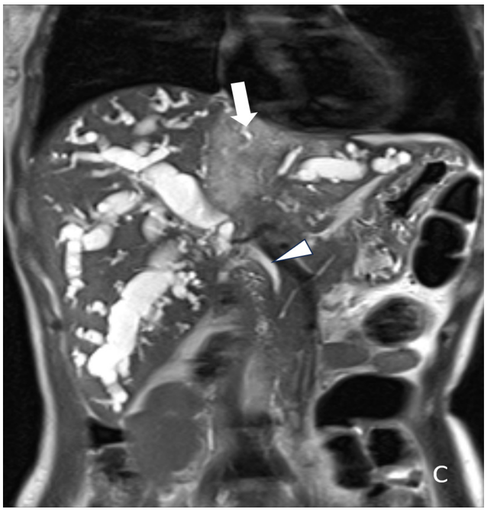

Figure 5c. Coronal T2WI MR Image Captured 4 Years after the Initial CT Scan, Revealing the Emergence of a New Mass (arrow) in the Left Hepatic Lobe Obstructing the Left Intrahepatic Bile Duct. Additionally, there is severe dilatation of the right intrahepatic bile duct and a relatively non-dilated CBD (arrowhead), suggesting mass obstruction to the hilar region.

p < 0.001), family history of CCA (RR = 1.92, p = 0.015), and diffuse bile duct dilatation (RR = 5.94, p < 0.001). There is multicollinearity between diffuse bile duct dilatation and periductal fibrosis [15], leading to a need to exclude the less significant factor of periductal fibrosis as an independent factor from the multivariate analysis. The results of both univariate and multivariate analyses are presented in Table 2.

In this cohort, one case presented with diffuse dilation of the biliary system detected during the initial screening (Figure 5a), without any identifiable cause of obstruction. Subsequent surveillance at one year continued to show the abnormality (Figure 5b). Four years after the initial detection, the patient eventually developed CCA (Figure 5c)."

Discussion

In our study conducted within a population residing in areas with high incidence rates of cholangiocarcinoma (CCA), we investigated predictive factors for premalignant CCA lesions. Our analysis revealed that ultrasonographic findings of bile duct dilatation, along with age and family history of first-degree relatives with CCA, emerged as

statistically significant predictors of CCA development. Notably, bile duct dilatation detected without a cause of obstruction emerged as a particularly accurate predictor compared to other factors examined.

While recent studies have not extensively explored bile duct dilatation as a predictor for cholangiocarcinoma (CCA), findings from a study by Shimonoshi et al. shed light on the histologic changes in the biliary tract infected by Opisthorchis Viverrini (OV). Their research revealed adenomatous hyperplasia of the biliary tract and goblet cell (peribiliary gland) metaplasia, resulting in increased mucin production and subsequent bile duct dilatation [12]. These findings align with those of Siripongsakun et al., who identified statistically significant ultrasonographic bile duct dilatation in patients with intraductal papillary neoplasm of the bile duct (IPNB)-type premalignant lesions of CCA [11]. Additionally, bile duct dilatation has been associated with biliary intraepithelial neoplasia (Bil-IN), indicating its potential as a marker for early premalignant changes in the biliary epithelium. Together, these studies support our findings that ultrasonographic bile duct dilatation can serve as a predictor for CCA.

In our study, periductal fibrosis emerged as another significant parameter. Catalano et al. highlighted the association between periductal inflammation and chronic Opisthorchis Viverrini (OV) infection [16], while Sripa et al. elucidated the pathophysiology, attributing it to chronic inflammation caused by OV infection leading to "granulomatous inflammation." This process involves the release of inflammatory cytokines such as interleukin-8 (IL-8) and Platelet-derived Growth Factor (PDGF), which stimulate the repair of the cells lining the biliary tract. The constant damage and repair increase the amount of epithelial cells and fibroblasts, ultimately resulting in periductal fibrosis [17]. Periductal fibrosis potentially serves as a finding of inflammation related to OV infection and may not be directly related to the last step of the cancerous change. This may explain the collinearity with the finding of diffuse bile duct dilatation.

Interestingly, our data support that both diffuse bile duct dilatation and periductal fibrosis are significant predictors for developing CCA. These findings are indicative of the inflammation process, which plays a crucial role in the pathogenesis of CCA. While a large cross-sectional CCA cohort study reported an association between diffuse bile duct dilatation and CCA [11]. our long-term follow-up study found that only diffuse bile duct dilatation emerged as a strong independent predictor for CCA development. We postulate that this discrepancy may be attributed to the specific nature of diffuse bile duct dilatation, which reflects bile duct changes related to inflammation, including mucin production [12] and potential premalignant changes such as intraductal papillary neoplasm of the bile duct (IPNB). Therefore, diffuse bile duct dilatation appears to be a more specific risk factor in the development of CCA [11].

Among demographic predictors, age and family history of CCA stand out as significant independent predictors for developing the disease. The worldwide incidence of CCA shows an increasing trend with age, reflecting its multifaceted evolution involving multiple carcinogenesis steps—from chronic bile duct inflammation to genetic alterations, premalignant bile duct changes, and eventual malignant transformation [18]. Age is consistently recognized as a risk factor for CCA globally, with the disease typically peaking in incidence around 50 years and above, and being uncommon before the age of 40 [9]; This suggests that the process of cholangiocarcinogenesis may require a considerable amount of time to progress to invasive carcinoma. In Thailand, the incidence of CCA begins to rise around the age of 40, reaching its peak around age 60 [19]. In our cohort study, most subjects detected with CCA through abdominal ultrasound surveillance were in their late 40s and early 50s, with none being younger than 40 years old [7].

Family factors influencing CCA development are believed to stem from both genetic inheritance and exposure to carcinogens [20]. In the context of liver fluke-related CCA development, household lifestyle practices may significantly contribute to risk exposure [20], characterized by poor sanitation practices, inadequate sewage infrastructure, and consumption of uncooked cyprinoid fish—a known class I carcinogen [21]. Consequently, individuals living in such environments face an elevated risk of liver-fluke infection. Therefore, a positive first-degree family history of CCA emerges as a robust predictor for CCA development, consistent with previous studies [22-24] underscoring the importance of incorporating this factor into surveillance schemes. Moreover, research on genetic and environmental causes has identified cell repair genes as playing a statistically significant role in CCA development [25, 20].

Studies from Khan et al. and Taylor et al. [26, 27] have consistently reported a higher incidence of CCA in men compared to women, with some studies suggesting a 1.2-1.5 times higher likelihood in men [22]. In alignment with these findings, our raw data also indicated a higher likelihood of CCA in men. However, upon statistical analysis, we found that sex was not a statistically significant predictor of CCA development, consistent with a study by Kamsa-ard et al, which reported only a mild association between sex and CCA [24].

Thailand bears the highest prevalence of CCA, particularly in the Northeastern region, imposing a significant healthcare burden [28]. Despite numerous studies investigating the characteristics of liver and biliary tract imaging for predicting CCA development [11, 13], conclusive evidence on their utility as predictors for CCA to guide surveillance and follow-up remains elusive. However, our previous report on CCA surveillance in endemic areas has demonstrated a survival benefit by detecting CCA in the resectable stage, potentially enabling curative R0 resection [7]. Unfortunately, specific serum biomarkers for CCA screening are lacking due to insufficient sensitivity and specificity [29]. Abdominal ultrasound (US) emerges as an effective primary method for detecting CCA and premalignant lesions, underscoring the importance of incorporating high-risk US predictors to triage the high-risk population for surveillance [11].

Incorporating ultrasound (US) predictors to prioritize patients for cholangiocarcinoma following initial

US screening could substantially reduce the need for continuous surveillance, with only around 20-30% of patients requiring follow-up when using criteria such as diffuse bile duct dilation and/or periductal fibrosis. However, approximately 8.7% of the country population in Thailand are infected with Opisthorchis viverrini, placing around 6 million individuals at increased risk [17]. Screening and monitoring for cholangiocarcinoma in this high-risk population pose substantial challenges, particularly in workforce requirements to ensure coverage across these regions. In Thailand, ultrasound examinations are primarily conducted by radiologists; however, due to radiologist shortages, comprehensive screening and surveillance are limited.

To address this workforce constraint, developing non-physician healthcare professionals, such as medical sonographers, to perform ultrasound screenings under radiologist supervision—using tele-imaging technology for oversight—may offer a viable solution. This approach could mitigate manpower issues and enable broader coverage for cholangiocarcinoma screening, thereby enhancing early detection and monitoring capabilities across at-risk regions.

This study has several limitations inherent to its retrospective design and the relatively small number of CCA cases. Additionally, our research was conducted in an endemic area of CCA where the primary risk factor was OV infestation, potentially limiting the generalizability of our findings to regions with different risk factors. Furthermore, the operator dependency of ultrasound (US) presents challenges, particularly in evaluating extrahepatic bile ducts, which may be obscured by bowel gas, leading to potential under-detection of extrahepatic CCA. However, it's worth noting that the incidence of extrahepatic CCA in our CCA surveillance cohort from Nan province was relatively low at 15.6% [6].

In summary, age and family history of CCA serve as potential clinically independent factors for targeting the at-risk population for extensive CCA screening in OV endemic areas. Moreover, diffuse bile duct dilatation emerges as an independent sonographic predictor for CCA development in patients at risk within OV-infested endemic areas. This imaging finding holds promise in identifying individuals requiring close CCA surveillance following initial ultrasound screening.

Author Contribution Statement

Natcha Thanakijsombat (1,2,3,4,5); Kamonwan Soonklang (1,3); Pantajaree Hiranrat (2,4,5); Poemporn Limpisook1,4; Surachate Siripongsakun (1,2,3,4,5).

Note: 1. Study concept and design 2. Acquisition of data 3. Analysis and interpretation of data 4. Drafting the manuscript 5. Critical revision of the manuscript

Acknowledgements

We sincerely thank Ban Luang District Hospital for their facilitation and support in data collection, which contributed significantly to the successful completion of this study. We also acknowledge the support provided by the Sonographer School, Faculty of Health Science Technology, Chulabhorn Royal Academy.

Funding Statement

Funding of this project is from HRH Chulabhorn Charity Project.

Ethical Declaration

The project was approved by the Ethics Committee for Human Research of Chulabhorn Research Institute (certificate no.0052/2661).

Patient consent statement:

All individual participants' consents have been waived.

Data availability statement

The data that support the findings of this study are available on request from the corresponding author (SS). The data are not publicly available due to ethical restrictions. The data are not publicly available due to privacy or ethical restrictions.

Conflict of Interest

The authors have no conflicts of interest corresponding to this article.

References

1. Schistosomes, liver flukes and helicobacter pylori. IARC working group on the evaluation of carcinogenic risks to humans. Lyon, 7-14 june 1994. IARC monographs on the evaluation of carcinogenic risks to humans. 1994;61:1-241.
2. Mosconi S, Beretta GD, Labianca R, Zampino MG, Gatta G, Heinemann V. Cholangiocarcinoma. Crit Rev Oncol Hematol. 2009;69(3):259-70. https://doi.org/10.1016/j.critrevonc.2008.09.008.
3. Sripa B, Pairojkul C. Cholangiocarcinoma: Lessons from thailand. Curr Opin Gastroenterol. 2008;24(3):349-56. https://doi.org/10.1097/MOG.0b013e3282fbf9b3.
4. Thunyaharn N, Promthet S, Wiangnon S, Suwanrungruang K, Kamsa-ard S. Survival of cholangiocarcinoma patients in northeastern thailand after supportive treatment. Asian Pac J Cancer Prev. 2013;14(11):7029-32. https://doi.org/10.7314/apjcp.2012.14.11.7029.
5. Khuntikeo N, Koonmee S, Sa-Ngiamwibool P, Chamadol N, Laopaiboon V, Titapun A, et al. A comparison of the proportion of early stage cholangiocarcinoma found in an ultrasound-screening program compared to walk-in patients. HPB (Oxford). 2020;22(6):874-83. https://doi.org/10.1016/j.hpb.2019.10.010.
6. Sungkasubun P, Siripongsakun S, Akkarachinorate K, Vidhyarkorn S, Worakitsitisatorn A, Sricharunrat T, et al. Ultrasound screening for cholangiocarcinoma could detect premalignant lesions and early-stage diseases with survival benefits: A population-based prospective study of 4,225 subjects in an endemic area. BMC Cancer. 2016;16:346. https://doi.org/10.1186/s12885-016-2390-2.
7. Siripongsakun S, Vidhyarkorn S, Charuswattanakul S, Mekraksakit P, Sungkasubun P, Yodkhunnathum N, et al. Ultrasound surveillance for cholangiocarcinoma in an endemic area: A prove of survival benefits. J Gastroenterol Hepatol. 2018;33(7):1383-8. https://doi.org/10.1111/jgh.14074.
8. Laopachee P, Siripongsakun S, Sangmala P, Chanree P,

Hiranrat P, Srisittimongkon S. Cost-effectiveness analysis of ultrasound surveillance for cholangiocarcinoma in an endemic area of thailand. Asian Pac J Cancer Prev. 2023;24(12):4117-25. https://doi.org/10.31557/APJCP.2023.24.12.4117.
9. Blechacz B. Cholangiocarcinoma: Current knowledge and new developments. Gut Liver. 2017;11(1):13-26. https://doi.org/10.5009/gnl15568.
10. Kamsa-Ard S, Luvira V, Suwanrungruang K, Kamsa-Ard S, Luvira V, Santong C, et al. Cholangiocarcinoma trends, incidence, and relative survival in khon kaen, thailand from 1989 through 2013: A population-based cancer registry study. J Epidemiol. 2019;29(5):197-204. https://doi.org/10.2188/jea.je20180007.
11. Siripongsakun S, Sapthanakorn W, Mekraksakit P, Vichitpunt S, Chonyuen S, Seetasarn J, et al. Premalignant lesions of cholangiocarcinoma: Characteristics on ultrasonography and mri. Abdom Radiol. 2019;44(6):2133-46. https://doi.org/10.1007/s00261-019-01951-2.
12. Shimonishi T, Sasaki M, Nakanuma Y. Precancerous lesions of intrahepatic cholangiocarcinoma. J Hepatobiliary Pancreat Surg. 2000;7(6):542-50. https://doi.org/10.1007/s005340070002.
13. Chamadol N, Khuntikeo N, Thinkhamrop B, Thinkhamrop K, Suwannatrai AT, Kelly M, et al. Association between periductal fibrosis and bile duct dilatation among a population at high risk of cholangiocarcinoma: A cross-sectional study of cholangiocarcinoma screening in northeast thailand. BMJ open. 2019;9(3):e023217. https://doi.org/10.1136/bmjopen-2018-023217.
14. Benedetti NJ, Desser TS, Jeffrey RB. Imaging of hepatic infections. Ultrasound Q. 2008;24(4):267-78. https://doi.org/10.1097/RUQ.0b013e31818e5981.
15. Stoltzfus JC. Logistic regression: A brief primer. Acad Emerg Med. 2011;18(10):1099-104. https://doi.org/10.1111/j.1553-2712.2011.01185.x.
16. Catalano OA, Sahani DV, Forcione DG, Czermak B, Liu CH, Soricelli A, et al. Biliary infections: Spectrum of imaging findings and management. Radiographics. 2009;29(7):2059-80. https://doi.org/10.1148/rg.297095051.
17. Sripa B, Brindley PJ, Mulvenna J, Laha T, Smout MJ, Mairiang E, et al. The tumorigenic liver fluke opisthorchis viverrini – multiple pathways to cancer. Trends Parasitol. 2012;28(10):395-407. https://doi.org/10.1016/j.pt.2012.07.006.
18. Nakanuma Y, Sasaki M, Sato Y, Ren X, Ikeda H, Harada K. Multistep carcinogenesis of perihilar cholangiocarcinoma arising in the intrahepatic large bile ducts. World J Hepatol. 2009;1(1):35-42. https://doi.org/10.4254/wjh.v1.i1.35.
19. Shaib Y, El-Serag HB. The epidemiology of cholangiocarcinoma. Seminars in liver disease. 2004;24(2):115-25. https://doi.org/10.1055/s-2004-828889.
20. Songserm N, Promthet S, Pientong C, Ekalaksananan T, Chopjitt P, Wiangnon S. Gene-environment interaction involved in cholangiocarcinoma in the thai population: Polymorphisms of DNA repair genes, smoking and use of alcohol. BMJ open. 2014;4(10):e005447. https://doi.org/10.1136/bmjopen-2014-005447.
21. Pengput A, Schwartz DG. Risk factors for opisthorchis viverrini infection: A systematic review. J Infect Public Health. 2020. https://doi.org/10.1016/j.jiph.2020.05.028.
22. Manwong M, Songserm N, Promthet S, Matsuo K. Risk factors for cholangiocarcinoma in the lower part of northeast thailand: A hospital-based case-control study. Asian Pac J Cancer Prev. 2013;14(10):5953-6. https://doi.org/10.7314/apjcp.2013.14.10.5953.
23. Tyson GL, El-Serag HB. Risk factors for cholangiocarcinoma. Hepatology. 2011;54(1):173-84. https://doi.org/10.1002/hep.24351.
24. Kamsa-ard S, Kamsa-ard S, Luvira V, Suwanrungruang K, Vatanasapt P, Wiangnon S. Risk factors for cholangiocarcinoma in thailand: A systematic review and meta-analysis. Asian Pac J Cancer Prev. 2018;19(3):605-14. https://doi.org/10.22034/apjcp.2018.19.3.605.
25. Honjo S, Srivatanakul P, Sriplung H, Kikukawa H, Hanai S, Uchida K, et al. Genetic and environmental determinants of risk for cholangiocarcinoma via opisthorchis viverrini in a densely infested area in nakhon phanom, northeast thailand. Int J Cancer. 2005;117(5):854-60. https://doi.org/10.1002/ijc.21146
26. Taylor-Robinson SD, Toledano MB, Arora S, Keegan TJ, Hargreaves S, Beck A, et al. Increase in mortality rates from intrahepatic cholangiocarcinoma in england and wales 1968-1998. Gut. 2001;48(6):816-20. https://doi.org/10.1136/gut.48.6.816.
27. Khan SA, Toledano MB, Taylor-Robinson SD. Epidemiology, risk factors, and pathogenesis of cholangiocarcinoma. HPB (Oxford). 2008;10(2):77-82. https://doi.org/10.1080/13651820801992641.
28. Sithithaworn P, Yongvanit P, Duenngai K, Kiatsopit N, Pairojkul C. Roles of liver fluke infection as risk factor for cholangiocarcinoma. J Hepatobiliary Pancreat Sci. 2014;21(5):301-8. https://doi.org/10.1002/jhbp.62.
29. Alvaro D. Serum and bile biomarkers for cholangiocarcinoma. Curr Opin Gastroenterol. 2009;25(3):279-84. https://doi.org/10.1097/mog.0b013e328325a894.

This work is licensed under a Creative Commons Attribution-Non Commercial 4.0 International License.
