# Supplementary material for: Periductal Fibrosis and Cholangiocarcinoma-Related Outcomes in Liver Fluke-Endemic Regions: A Systematic Review and Meta-Analysis
Source: Med Sci (Basel). 2026 Jul 9;14(3):380. doi: 10.3390/medsci14030380 (PMC13414246; doi:10.3390/medsci14030380)
Supplement: Supplementary file 1 [file medsci-14-00380-s001.zip › Table S1. Search strategy.pdf]

Supplementary Table S1. Detailed Search Strategy Used for Each Database

**Database**	**Search Strategy**	**Results**
PubMed (1 April 2026)	("Periductal Fibrosis"[Title/Abstract] OR "periportal fibrosis"[Title/Abstract] OR "biliary fibrosis"[Title/Abstract] OR PDF[Title/Abstract]) AND ("Cholangiocarcinoma"[MeSH Terms] OR "cholangiocarcinoma"[Title/Abstract] OR "bile duct cancer"[Title/Abstract] OR CCA[Title/Abstract]) AND ("Opisthorchis viverrini"[MeSH Terms] OR "opisthorchis viverrini"[Title/Abstract] OR "liver fluke"[Title/Abstract] OR opisthorchiasis[Title/Abstract]) AND (humans[MeSH Terms])	49
Scopus (1 April 2026)	TITLE-ABS-KEY ("periductal fibrosis" OR "periportal fibrosis" OR "biliary fibrosis" OR PDF) AND TITLE-ABS-KEY ("cholangiocarcinoma" OR "bile duct cancer" OR CCA) AND TITLE-ABS-KEY ("Opisthorchis viverrini" OR "liver fluke" OR opisthorchiasis)	81
Embase (1 April 2026)	('periductal fibrosis' OR 'periportal fibrosis' OR 'biliary fibrosis' OR PDF) AND ('cholangiocarcinoma'/exp OR 'cholangiocarcinoma' OR 'bile duct cancer' OR CCA) AND ('opisthorchis viverrini'/exp OR 'liver fluke' OR opisthorchiasis) AND [humans]/lim	189

Abbreviations: PDF, periductal fibrosis; CCA, cholangiocarcinoma. The search strategy combined controlled vocabulary terms and free-text keywords and was adapted for each database.
