# Supplementary material for: Periductal Fibrosis and Cholangiocarcinoma-Related Outcomes in Liver Fluke-Endemic Regions: A Systematic Review and Meta-Analysis
Source: Med Sci (Basel). 2026 Jul 9;14(3):380. doi: 10.3390/medsci14030380 (PMC13414246; doi:10.3390/medsci14030380)
Supplement: Supplementary file 1 [file medsci-14-00380-s001.zip › Table S2. Methodological quality assessment.pdf]

Table S2. Methodological quality assessment of included studies.

1. JBI critical appraisal checklist for prevalence studies

	Study	1. Was the sample frame appropriate to address the target population?	2.Were study participants sampled in an appropriate way?	3.Was the sample size adequate?	4.Were the study subjects and the setting described in detail?	5.Was the data analysis conducted with sufficient coverage of the identified sample?	6.Were valid methods used for the identification of the condition?	7.Was the condition measured in a standard, reliable way for all participants?	8.Was there appropriate statistical analysis?	9.Was the response rate adequate, and if not, was the low response rate managed appropriately?	% Yes	Risk*
1	Moungthard et al., 2023	Yes	Unclear	Yes	Yes	Yes	Yes	Yes	Yes	Unclear	77.78	Low
2	Soukhathammavong et al., 2015	Yes	Yes	Unclear	Yes	Yes	Yes	Yes	Yes	Yes	88.89	Low
3	Thanakijsombat et al., 2024	Yes	Yes	Yes	Yes	Yes	Yes	Yes	Yes	Yes	100	Low
4	Mairiang et al., 2012	Yes	Unclear	Yes	Yes	Yes	Yes	Yes	Yes	Yes	89.89	Low
5	Homsana et al., 2024	Yes	Unclear	Yes	Yes	Yes	Yes	Yes	Yes	Unclear	77.78	Low

* High risk of bias = score up to 49% of "yes" scores; Moderate risk of bias = score from 50 to 69% of "yes" scores; Low risk of bias = score more than 70% of "yes" scores.

2. Newcastle–Ottawa Scale (NOS) Quality Assessment

	Study	Study Design	Selection (★)	omparability (★	Outcome / Exposure (★)	Total Score (★)	Quality
1	Moungthard et al., 2023	Cross-sectional study	★★★	★★	★★	★★★★★★★☆☆ (7)	High
2	Thanakijsombat et al., 2024	Cohort study	★★★★	★★	★★★	★★★★★★★★★ (9)	High
3	Homsana et al., 2024	Cross-sectional study	★★★	★★	★★	★★★★★★★☆☆ (7)	High

Study quality was assessed using a modified Newcastle–Ottawa Scale (NOS) for observational studies. The modified NOS evaluates three domains: Selection (maximum 4 stars), Comparability (maximum 2 stars), and Outcome/Exposure (maximum 3 stars), with a maximum total score of 9 stars for all study designs, including cohort and cross-sectional studies. Selection assesses representativeness of the study population, sample selection, sample size adequacy, non-response, and ascertainment of exposure. Comparability assesses adjustment for key confounders (at minimum age and sex, with additional adjustment where reported). Outcome/Exposure assesses outcome ascertainment, statistical analysis, adequacy of follow-up (for cohort studies), and reliability of exposure or outcome assessment, depending on study design. Studies scoring 7–9 stars were considered high quality, 4–6 stars moderate quality, and 0–3 stars low quality.
