# Supplementary figures and images for: Periductal Fibrosis and Cholangiocarcinoma-Related Outcomes in Liver Fluke-Endemic Regions: A Systematic Review and Meta-Analysis
Source: Med Sci (Basel). 2026 Jul 9;14(3):380. doi: 10.3390/medsci14030380 (PMC13414246; doi:10.3390/medsci14030380)

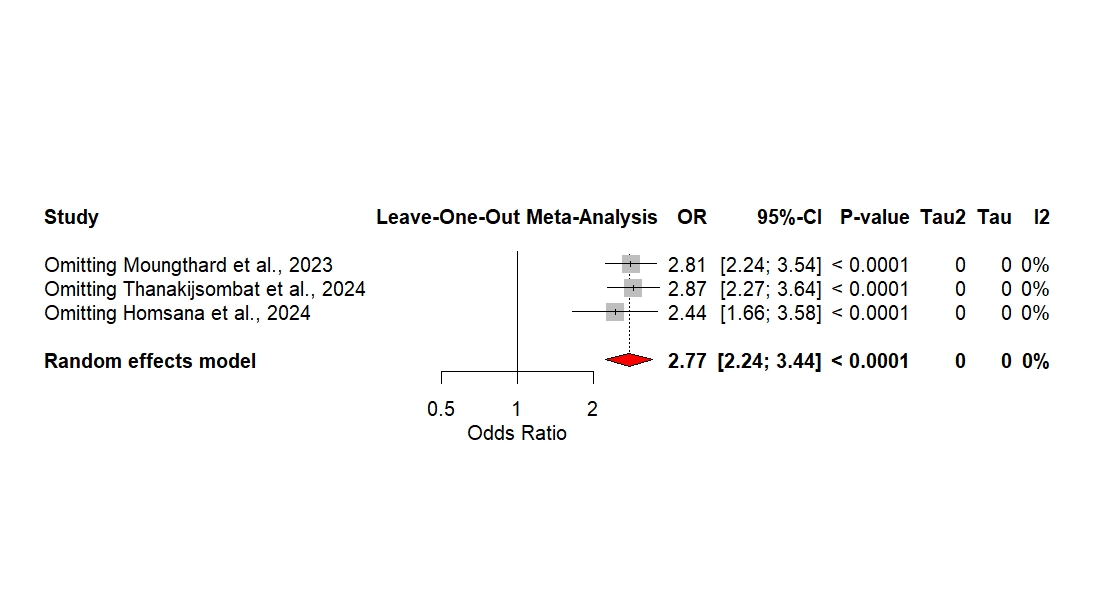

Supplement: Supplementary file 1 [file medsci-14-00380-s001.zip › Supplementary Figure S1..jpeg]

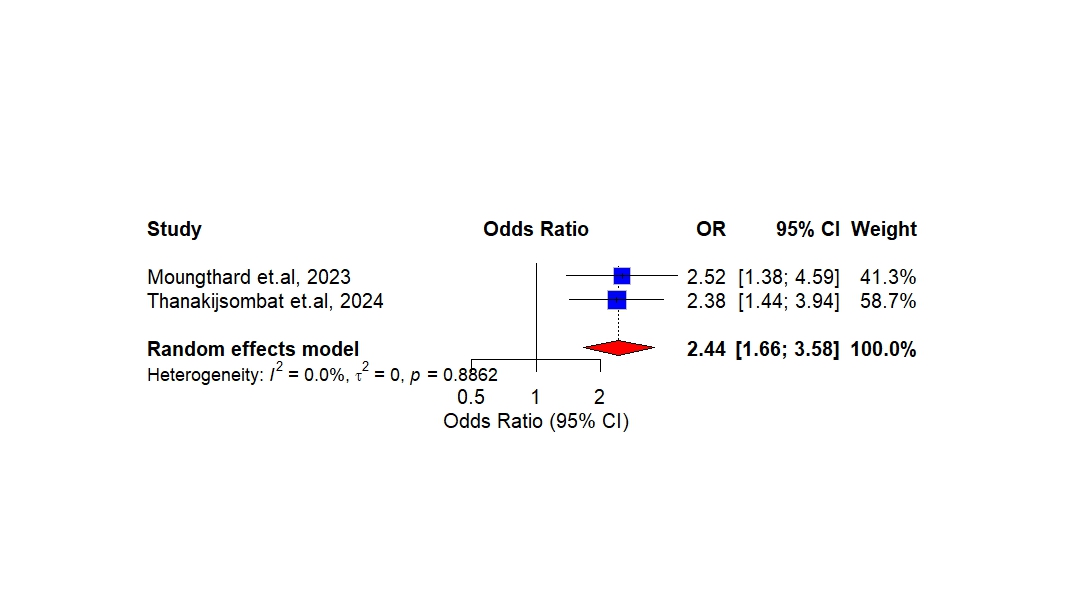

Supplement: Supplementary file 1 [file medsci-14-00380-s001.zip › Supplementary Figure S2..jpeg]

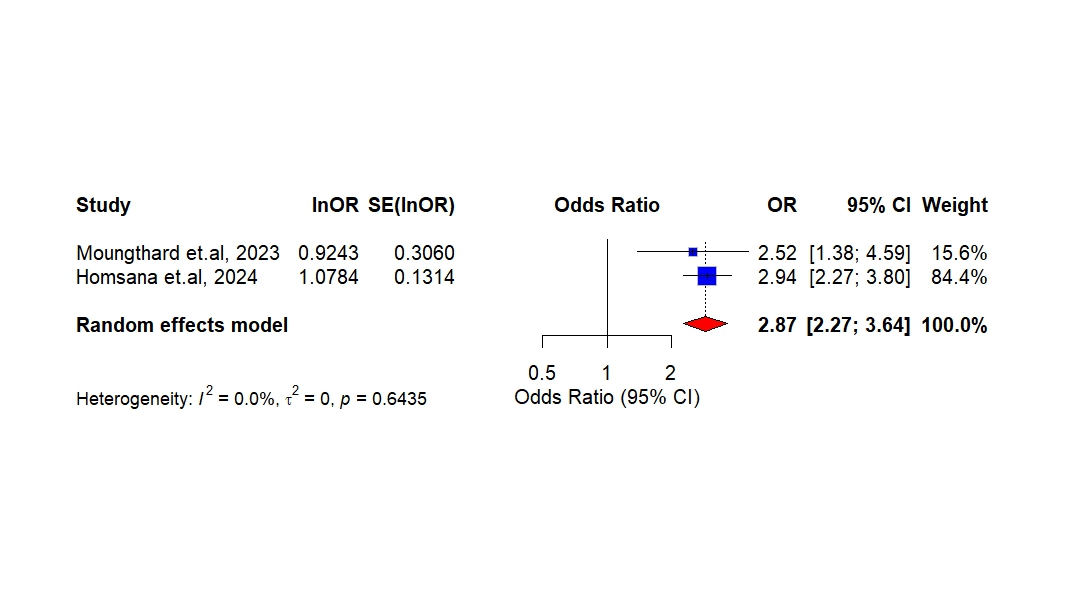

Supplement: Supplementary file 1 [file medsci-14-00380-s001.zip › Supplementary Figure S3..jpeg]

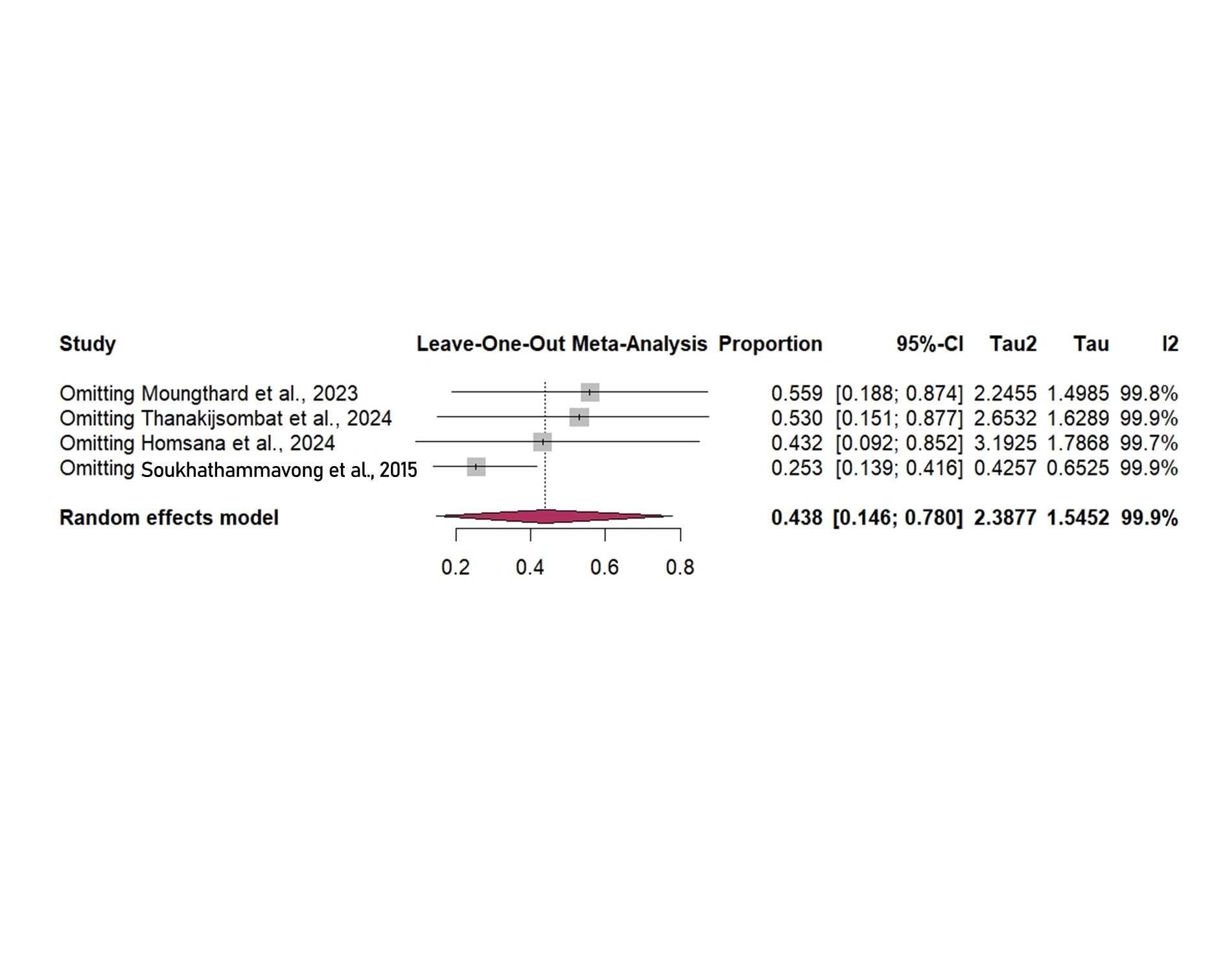

Supplement: Supplementary file 1 [file medsci-14-00380-s001.zip › Supplementary Figure S4..jpg]

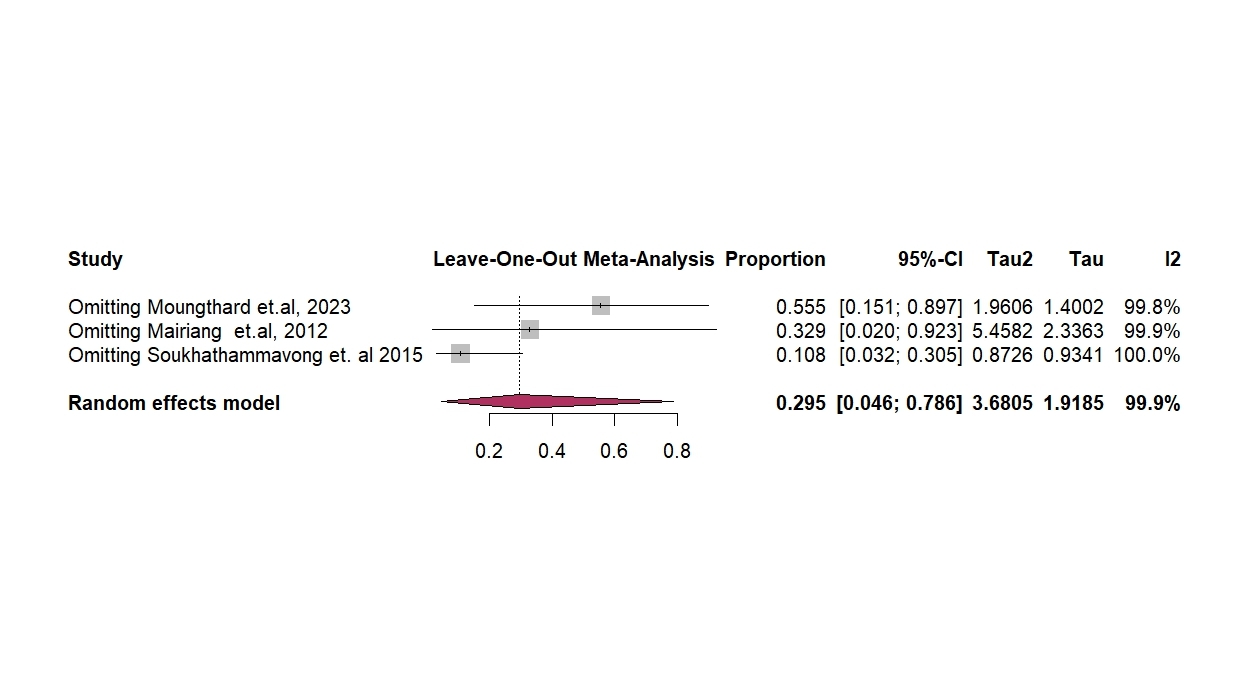

Supplement: Supplementary file 1 [file medsci-14-00380-s001.zip › Supplementary Figure S5..jpg]
